# Fortification of Staple Foods for Household Use with Vitamin D: An Overview of Systematic Reviews

**DOI:** 10.3390/nu15173742

**Published:** 2023-08-26

**Authors:** Patrick Nyamemba Nyakundi, Zsuzsanna Némethné Kontár, Attila Kovács, Luca Járomi, Afshin Zand, Szimonetta Lohner

**Affiliations:** 1 Department of Public Health Medicine, Medical School, University of Pécs, 7624 Pécs, Hungary; 2 Doctoral School of Health Sciences, Faculty of Health Sciences, University of Pécs, 7621 Pécs, Hungary; 3 Cochrane Hungary, Clinical Center of the University of Pécs, Medical School, University of Pécs, 7623 Pécs, Hungary

**Keywords:** food fortification, health outcomes, vitamin D, health impact, vitamin D deficiency, serum 25(OH)D, parathormone, prevention, overview of reviews

## Abstract

Vitamin D deficiency is a global public health concern with significant implications for bone health and chronic disease prevention. Our aim was to summarize the evidence from Cochrane and other systematic reviews evaluating the benefits or harms of vitamin D fortification of staple foods for household use. In April 2023, we systematically searched Ovid MEDLINE, Embase, Epistemonikos and the Cochrane Database of Systematic Reviews for systematic reviews investigating the effects of vitamin D fortification of food in general populations of any age. We used Cochrane methodology and assessed the methodological quality of included studies using AMSTAR (A MeaSurement Tool to Assess Systematic Reviews). We assessed the degree of overlap among reviews. All outcomes included in systematic reviews were assessed. The protocol is registered in PROSPERO (registration number: CRD42023420991). We included 27 systematic reviews out of 5028 records for analysis. Overall, 11 out of 12 systematic reviews calculating pooled estimates reported a significant increase in serum 25(OH)D concentrations. The mean change in serum 25(OH)D concentrations per additional 100 units of vitamin D ranged from 0.7 to 10.8 nmol/L. Fortification of food with vitamin D showed a reduction in the prevalence of vitamin D deficiency based on high-certainty evidence. Parathormone (PTH) levels were described to decrease, bone mineral density to increase, while the effects on other bone turnover markers were inconsistent. Fortification did not significantly impact most anthropometric parameters, but it seemed to positively influence lipid profiles. In summary, fortification of food with vitamin D results in a reduction of vitamin D deficiency and might increase serum 25(OH)D concentrations, to varying extents depending on the fortified vehicle and population characteristics. Additionally, fortification may have a positive impact on bone turnover and lipid metabolism but may only have a limited effect on anthropometric parameters.

## 1. Introduction

Vitamin D is an essential micronutrient that plays a critical role in maintaining plasma levels of calcium and phosphorus and facilitates proper bone mineralization [1,2,3]. Vitamin D exists in two important forms: ergocalciferol (vitamin D2) and cholecalciferol (vitamin D3). These compounds are derived from precursors found in plants (ergosterol) and in the skin (dehydrocholesterol) [4]. When exposed to ultraviolet rays from the sun, these precursors are converted into provitamins D2 and D3. Further transformations occur in the liver and kidneys, resulting in the active form of vitamin D (calcitriol) [5]. While the body can synthesize vitamin D through exposure to sunlight, dietary intake also serves as a significant source. Foods rich in vitamin D include fatty fish (such as salmon and mackerel) [2,6], fortified dairy products (such as milk and yogurt), eggs, and certain mushrooms [7].

Vitamin D deficiency is a widespread concern affecting populations worldwide [1,7,8,9]. Various studies and guidelines have proposed different cutoff levels to define vitamin D deficiency. Most authors consider a range below 75 nmol/L (or 30 ng/mL) of serum or plasma 25(OH)D concentration as vitamin D deficiency [10,11,12]. However, a more critical threshold of <25 or <30 nmol/L (or 10/12 ng/mL) is associated with a significantly higher risk of osteomalacia and nutritional rickets, leading to the classification of severe vitamin D deficiency [13,14]. The Endocrine Society Task Force on Vitamin D’s clinical practice guidelines define a cutoff level of 50 nmol/L as vitamin D deficiency [10]. In infants and young children, a serum concentration of 25-OH-D below approximately 27.5 nmol/L (11 ng/mL) indicates a deficiency in vitamin D [15]. In the context of public health, preventing vitamin D levels below 30 nmol/L (or 12 ng/mL) is essential, and public health approaches should be employed to address this issue effectively [16]. Recent reports have provided estimates of the prevalence of vitamin D deficiency in representative population samples in Europe, Canada, and the US, based on standardized serum 25(OH)D levels below 30 nmol/L. The reported prevalence rates for these regions are 13%, 7.4%, and 5.9%, respectively [7,17,18]. In the Mediterranean region, without a food fortification policy, vitamin D deficiency has been reported as high as 36% [19]. Vitamin D levels below 30 nmol/L (or 12 ng/mL) are prevalent in more than 20% of the population in countries like India, Tunisia, Pakistan, and Afghanistan [20]. Based on these estimates, approximately 490 million individuals in India alone are estimated to suffer from vitamin D deficiency [7,16].

Factors such as limited sunlight exposure due to geographical location, seasonal variations, cultural practices, or lifestyle choices contribute to inadequate vitamin D synthesis in the body [6]. Additionally, dietary patterns that exclude or limit consumption of vitamin D-rich foods can exacerbate the problem. In regions with limited access to sunlight, individuals may struggle to achieve adequate vitamin D levels solely through sun exposure [6,21]. This is particularly true in areas with long winters, high levels of air pollution, or lifestyles that involve indoor work or limited outdoor activities. Moreover, cultural practices like wearing concealing clothing or using sunscreen with a high sun protection factor (SPF) can further hinder the synthesis of vitamin D in the skin [22]. Dietary habits also play a crucial role in vitamin D status [6,23]. Many natural food sources of vitamin D are limited in the average diet, therefore, reaching the optimal vitamin D intake through dietary diversification might be difficult. Specific food patterns, like high consumption of processed food and low consumption of vegetables, fruits, nuts, whole grains, and fish may also predispose people to an inadequate vitamin D status [24].

As a result, the prevalence of vitamin D deficiency has become a significant public health concern [25]. Insufficient vitamin D levels have been associated with a range of health issues, including weakened bones, increased risk of fractures, osteoporosis, immune dysfunction, and an elevated susceptibility to certain chronic diseases such as cardiovascular disease, diabetes, and certain cancers [1].

As a potential strategy, fortifying staple foods provides an accessible and convenient way to improve the population’s vitamin D intake [16,26,27]. Fortification programs have been implemented in numerous countries, such as Finland, the US, Denmark, and Canada, as a means of addressing vitamin D insufficiency and its associated health risks [15,16]. Compared to supplementation, which only reaches a limited proportion of the population, food fortification offers significantly broader coverage, ensuring equitable access to vitamin D and its associated health benefits across diverse population groups.

Fortification can either be mandatory, required and enforced by government policies, or voluntary, where the manufacturers have the liberty to decide whether to fortify the food products or not [15]. The fortified foods vary by country and may include items like margarine, milk, dairy drinks, cereals, biscuits, fruit juices, and more [28].

To date, several systematic reviews have been conducted to examine the effects of vitamin D fortification on various outcomes. However, there is a need for a comprehensive overview that systematically synthesizes the available information. This overview aims to bridge the existing knowledge gap by integrating and analyzing the results of previous systematic reviews and providing a clearer picture of the overall impact of fortifying staple foods for household use with vitamin D. This synthesis of information will offer a more holistic and robust understanding of the benefits or potential harms associated with vitamin D fortification initiatives, serving as a valuable resource for policymakers, researchers, and public health professionals.

## 2. Materials and Methods

### 2.1. Search Strategy and Selection Criteria

The methods of this overview of reviews are based on the Cochrane Handbook for Systematic Reviews of Interventions [29]. A priori protocol was registered in PROSPERO with registration number: CRD42023420991. The report of this overview of reviews follows the PRIOR (preferred reporting items for overviews of review) recommendations [30]. The PRIOR checklist can be found in Supplementary File S1. The selection process is reported in the PRISMA (The Preferred Reporting Items for Systematic Reviews and Meta-Analysis) flow chart [31].

Ovid MEDLINE (ovidsp.ovid.com), Embase (www.embase.com), Epistemonikos (www.epistemonikos.org), and the Cochrane Database of Systematic Reviews (www.cochranelibrary.com) were searched from inception to 19 April 2023 for systematic reviews on vitamin D fortification of staple foods. The search strategy combined indexing terms and text words related to the concepts of vitamin D and fortification, and incorporated validated search filters for systematic reviews and meta-analyses [32,33]. For the search strategy used, see Supplementary File S2.

To be included, the review needed to meet all the following criteria: (a) a systematic review with or without meta-analyses; (b) systematic reviews including studies conducted in the general population (including also pregnant women) of any age; (c) eligibility criteria of the systematic review including fortification of vitamin D as an eligible intervention (either as a standalone fortification or in combination with other vitamins and minerals); (d) eligibility criteria of the systematic review including unfortified food as an eligible comparison. Systematic reviews which included only uncontrolled studies, or exclusively examined populations with specific diseases, were excluded. Any outcome investigated in the included systematic reviews was of interest.

Two authors screened independently titles and abstracts for each study (PNN, ZNK, SL, LJ, AZ, AK). In the full-text screening phase two reviewers independently searched and reviewed articles for eligibility (SL, PNN, ZNK, AK). Discrepancies were resolved through consensus.

### 2.2. Data Extraction

We extracted the following characteristics of the included systematic reviews: the date of the search, the number of participants and trials included, the stated objective(s) of the review, the type of participants involved, the geographical settings (countries) covered in the studies, details of the intervention or exposure under investigation, the specific comparison made within the review, eligible outcomes, and GRADE (Grading of Recommendations, Assessment, Development, and Evaluations) assessment results, if available. Related to the results of included reviews, we extracted the following data: the specific comparison made within each review, the outcome(s) assessed, the number of trials reporting on each outcome, and the number of included participants, and the results reported, which were derived either from meta-analysis or narrative description (Tables S1–S5, Supplementary File S3). We also collected information related to the subgroup analyses conducted in the included systematic reviews and their results. The measurement units of vitamin D were converted to IU (1 µg of vitamin D = 40 IU). After finalizing the first data extraction, a second reviewer checked the collected data. All disagreements were resolved by consensus.

The methodological quality of the systematic reviews was assessed using the AMSTAR (A MeaSurement Tool to Assess Systematic Reviews) tool [34]. The reviews were evaluated by two reviewers, and any disagreements were resolved through discussion.

### 2.3. Data Synthesis

We summarized the data from the individual reviews narratively and presented these summaries using tables (Tables S1–S5, Supplementary File S3). Association between fortification characteristics (e.g., dose used, intervention duration) and average change in serum 25(OH)D concentrations are presented using tables and complex diagrams. We used bubble plots to show outcomes investigated in individual studies. We assessed the degree of overlap among reviews by collecting data on the individual studies included in the systematic reviews, which are also presented using table charts.

## 3. Results

**Figure 1:**
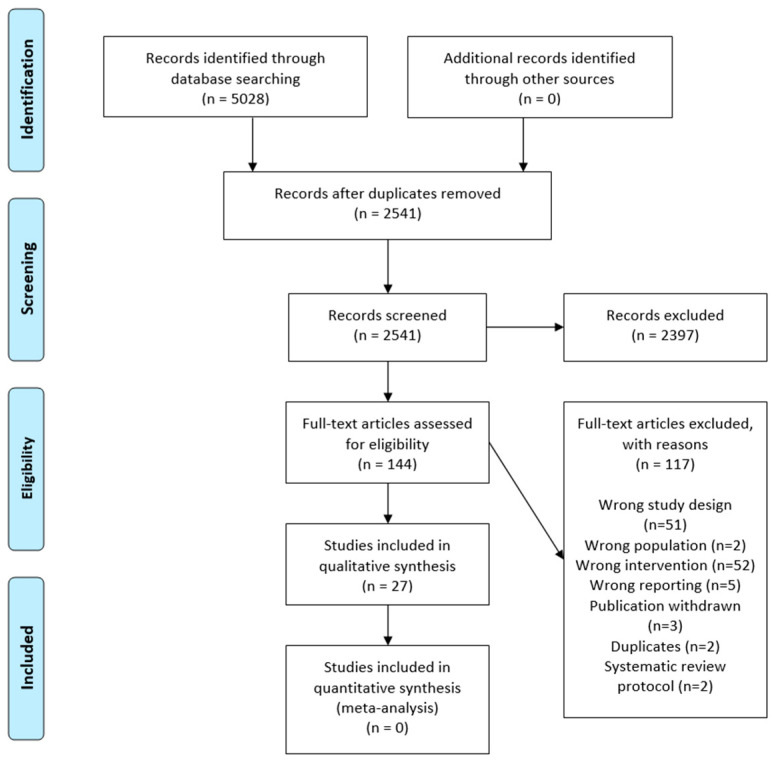
PRISMA flow diagram.

The systematic search yielded 5028 records. After removing 2487 duplicates, we screened 2541 records based on title and abstract. A total of 144 publications were assessed for eligibility based on full text. The excluded articles and the reason for exclusion are listed in Supplementary File S4. Finally, 27 systematic reviews fulfilled the eligibility criteria and were included in this overview of systematic reviews [35,36,37,38,39,40,41,42,43,44,45,46,47,48,49,50,51,52,53,54,55,56,57,58,59,60,61]. The selection process is shown in Figure 1.

### 3.1. Description of Included Systematic Reviews

We summarized the baseline characteristics of the included systematic reviews in Table 1. All data collected from the systematic reviews are shown in Tables S1–S5, Supplementary File S3. The search date in the included systematic reviews were between 2006 and 2022. The systematic reviews included 2 to 40 vitamin D fortification trials.

Of the 27 systematic reviews, 5 included children only [44,56,57,58,60], and 7 included exclusively adults [36,42,43,46,47,48,59], while in 15 reviews all age groups were included [35,37,38,39,40,41,45,49,50,51,52,53,54,55,61]. Most of the systematic reviews included studies of both sexes, with the exception of two studies including only women [48,53].

While most of the studies did not apply restrictions based on the geographic location of the study [35,36,37,38,39,40,41,42,44,45,46,47,48,49,50,51,52,53,54,55,56,57,58,59,60,61], one systematic review included only RCTs from Iran [43]. Only eight systematic reviews reported data on other forms of vitamin D intake, e.g., from sun exposure or dietary vitamin D intake from other foods or supplements [35,36,38,40,41,52,58,59].

The fortified vehicle in the included studies was a dairy product in seven systematic reviews [36,39,47,48,53,56,58] and bread in two systematic reviews [38,61], while all types of food vehicles were included in the rest of the reviews. The types of vehicles included in the systematic reviews are shown in Table S3, Supplementary File S3. The dose of fortification varied between 60–5000 IU/day in most systematic reviews, with the exception of two studies that used 28,000 IU/day of vitamin D for fortification [49,51]. The intervention duration ranged from three weeks to 30 months in the studies included. Only limited information was available on the type of vitamin D compound (Table S3, Supplementary File S3).

Among the 27 included systematic reviews, 22 reported the effect of vitamin D fortification on serum 25(OH)D concentrations [35,36,37,38,40,41,42,43,44,45,46,47,48,51,52,53,55,56,57,58,59,60], 9 reviews reported serum parathormone (PTH) levels [37,38,40,41,46,47,48,51,53], 3 reported serum calcium (Ca) [38,40,53] and 4 systematic reviews reported adverse effects [35,41,55,58]. Five systematic reviews examined the effect of vitamin D-fortified foods on bone markers e.g., osteocalcin (OC), alkaline phosphatase (ALP), total procollagen type 1 N-terminal propeptide (P1NP), serum telopeptides of type-1 collagen (CTX) and bone mineral density (BMD) [37,38,48,51,53]. Regarding anthropometric parameters, two reviews described the effect of food fortification with vitamin D [47,50]. Five systematic reviews described the results on glucose metabolism [39,40,47,49,51], two studies reported blood lipid levels and blood pressure [39,47]. Only one study investigated the effect of vitamin D-fortified food on the prevalence of vitamin D deficiency [60], cancer mortality [45], school performance [60], cognitive function [60], infection rate [60], and the cost-effectiveness of fortification [61].

GRADE assessment of results was performed by only two systematic reviews [53,60]. The majority of the systematic reviews assessed risk of bias of the included trials: the Jadad-scale was used by 11 reviews [35,37,41,43,49,51,52,54,55,57,59] and the Cochrane Risk of Bias Tool by 13 reviews [39,42,43,44,47,48,50,53,54,56,57,58,60]. Risk of bias assessment was not reported in six reviews [36,38,40,45,46,61].

The methodological quality of included systematic reviews assessed using AMSTAR can be found in Table S4, Supplementary File S3 [34]. The mean number of “Yes” answers across the systematic reviews was 6.89 (the median was 7) out of 11. Two reviews were assessed as high quality, where only the conflict of interest of the primary studies was missing [35,60]. In contrast, three reviews demonstrated a high risk of bias, fulfilling the requirements only in 3 fields of AMSTAR assessment [36,40,45]. All of the included systematic reviews established the research question and inclusion criteria in advance, and most of the systematic reviews provided information about the characteristics of the included studies (*n* = 25) and the included publication type (*n* = 25). On the other hand, there are potential sources of biases: the excluded trials were not listed in 92.6% (*n* = 25) of the systematic reviews, and the conflict of interest of the primary studies was not addressed in 88.9% (*n* = 24) of the systematic reviews. In 55.6% (*n* = 15) of the included systematic reviews, the study quality was not considered in formulating conclusions, although it was not assessed only in 18.5% (*n* = 5) of the reviews. Publication bias was not assessed in 55.6% (*n* = 15) of the studies. Meta-analysis was not conducted in 33.3% (*n* = 9) of the systematic reviews. Duplicate study selection and data extraction were not reported in 25.9% (*n* = 7) of the systematic reviews. A comprehensive literature search was not performed, or keywords were not provided in 25.9% (*n* = 7) of the systematic reviews.

### 3.2. Effect of Vitamin D Fortification

#### 3.2.1. Effect of Vitamin D Fortification on Serum 25(OH)D Concentrations

A total of 22 systematic reviews included evidence for the effect of vitamin D fortification on serum 25(OH)D concentrations [35,36,37,38,40,41,42,43,44,45,46,47,48,51,52,53,55,56,57,58,59,60]. Meta-analysis was undertaken in 12 systematic reviews [37,41,42,43,44,47,51,52,53,57,59,60], while 10 systematic reviews reported the findings narratively [35,36,38,40,45,46,48,55,56,58].

Overall, 11 out of 12 systematic reviews calculating pooled estimates described an increase in serum 25(OH)D concentrations. In the 11 systematic reviews describing a significant positive effect [37,41,42,43,44,47,51,52,57,59,60], on average, fortification increased serum 25(OH)D concentrations by 6.9 to 34.7 nmol/L (Table 2). In one systematic review analyzing different age groups separately [53] meta-analysis results showed that vitamin D fortification increased serum concentration of 25(OH)D3 in children (SMD 1.23, 95% CI 0.35 to 2.11, 7 trial, moderate quality of evidence) and post-menopausal women (SMD 0.82, 95% CI 0.30 to 1.34, GRADE: moderate certainty evidence), but not in women of reproductive age (−1.10, 95% CI −3.81 to 1.60, GRADE: moderate certainty evidence). Additionally, 10 systematic reviews summarized results on 25(OH)D concentrations narratively [35,36,38,40,45,46,48,55,56,58]. All of these systematic reviews reported studies with elevated serum 25(OH) vitamin D concentrations in the fortified group versus the control group. Although three reviews described a few studies as well, the results were not significant [56,58] or decreased according to the control group [40].

**Figure 2:**
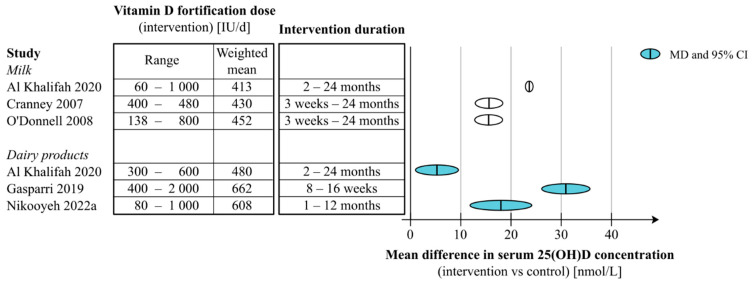
Association between fortification dose used, intervention duration and average change in serum 25(OH)D concentrations, based on systematic reviews quantitatively summarizing effects of milk or dairy product fortification with vitamin D [35,41,44,47,60].

Four systematic reviews summarized the effects of milk fortification quantitatively (either as the pooled effect estimate of all included studies or as a subgroup-analysis) [35,41,53,60], additionally, 1 review summarized the findings on milk fortification qualitatively [56]. Three assessed vitamin D fortification of dairy products quantitatively [42,47,60], and a further four systematic reviews described the results on dairy products narratively [36,39,48,58]. The range of fortification doses used in the milk and dairy product fortification studies summarized by meta-analysis and the pooled effect on serum 25(OH)D concentration is shown in Figure 2.

Two systematic reviews compared the effects of different types of vehicles as a subgroup-analysis [42,60]. One of the systematic reviews reported that milk increased serum 25(OH)D concentration by an MD of 23.72 nmol/L (95% CI 22.86 to 24.58; *I*^2^ = 99%), juice: 11.80 nmol/L (95% CI 7.35 to 16.26; *I*^2^ = 0%), cereal: 8.93 nmol/L (95% CI −0.36 to 18.21; *I*^2^ = 40%), and yogurt and cheese increased 25(OH)D concentration by an MD of 5.34 nmol/L (95% CI 0.97 to 9.70; *I*^2^ = 49%) [60]. The other systematic review did not analyze the effect of milk separately but included a study with oil fortification, which seemed to have the highest treatment effect: oil (*n* = 1): 40.50 nmol/L (95% CI 30.65 to 50.35); Juice (*n* = 5): 34.40 nmol/L (95% CI 31.46 to 37.33); Grain products (*n* = 5): 31.72 nmol/L (95% CI 18.42 to 45.01); Dairy and grain products (*n* = 2): 25.66 nmol/L (95% CI 18.32 to 33.00); dairy products (*n* = 19): 21.25 nmol/L (95% CI 12.51 to 29.98) [42].

A total of nine systematic reviews reported on the association between dose and serum 25(OH)D concentrations [36,42,43,44,54,55,57,59,60]. Their results are summarized in Table 3. The mean change of serum 25(OH)D concentrations for each additional 100 units of vitamin D ranged between 0.7 and 10.8 nmol/L, depending on the fortified vehicle and characteristics of the population. In a systematic review focusing on the fortification of dairy products, a 1–2 nmol/L increase of serum 25(OH)D was seen with every 100 IU vitamin D administered [55]. In a systematic review focusing on the fortification of yogurt, a mean change of 5.05 nmol/L was reported for serum 25(OH)D for every 100 IU vitamin D administered [36]. Based on the population age, the mean change of serum 25(OH)D concentrations for 100 IU vitamin D administered ranged between 0.7 and 6.9 nmol/L in the systematic reviews including only children [44,54,57,60], and ranged between 2 and 6.5 nmol/L in the systematic reviews including only adults [42,43,54,59].

Four systematic reviews considered the effect of the vitamin D dose administered in the subgroup analysis [37,42,52,59]. Although the dose of vitamin D chosen as a cutoff value in the subgroup-analysis was different (400 IU/day, 400 IU/day, 1000 IU/day, and 4000 IU/day, respectively), every systematic review reported greater treatment effect in the subgroup with fortification dose above the cutoff value.

One systematic review assessing data from RCTs with vitamin D3-fortifed foods undertook an individual participant data (IPD)-level meta-analysis of the response of winter serum 25(OH)D to total vitamin D intake among children and adults [54]. Authors found that “IPD-derived vitamin D intakes required to maintain 90%, 95%, and 97.5% of winter 25(OH)D concentrations ≥50 nmol/L are much higher than those derived from standard meta-regression based on aggregate data, due to the inability of the latter to capture between person-variability” [54]. The intake estimates to maintain 90%, 95%, and 97.5% of concentrations ≥50 nmol/L were found to be 17.0, 28.1, and 43.6 µg/day, respectively, when analyses were adjusted for baseline serum 25(OH)D, age, and body mass index (BMI). The authors concluded that 12 µg/day of vitamin D, supplied by fortified foods together with habitual intake, can prevent wintertime vitamin D deficiency (serum 25(OH)D <30 nmol/L) in the vast majority of individuals [54].

**Figure 3:**
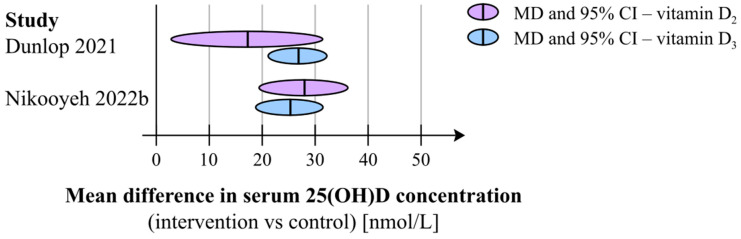
Comparison of the effects of fortification with vitamin D2 or D3 [42,52].

Two systematic reviews compared the effects of vitamin D2 and D3 fortification as part of subgroup analyses (Figure 3) [42,52]. One of the systematic reviews reported greater effects on serum 25(OH)D for vitamin D3 (25 studies) (effect size 26.8 nmol/L; 95% CI: 21.1 to 32.5; *I*^2^ = 97%) than for vitamin D2 (4 studies) (effect size 17.2 nmol/L; 95% CI: 2.78 to 31.7; *I*^2^ = 96%); however, heterogeneity was high [52]. The other systematic review reported no difference between trials that used vitamin D2 or D3 as a fortificant (MD 27.9 nmol/L, 95% CI 19.3 to 36.4 vs. MD 25.2 nmol/L, 95% CI 18.7 to 31.7, *p* = 0.62); however, only two trials assessed the effect of vitamin D2 [42].

Four systematic reviews investigated the effect of the baseline levels of serum 25(OH)D either as subgroup-analyses or separately [41,44,52,59]. All of these studies reported greater treatment effects when the baseline serum 25(OH)D level was <50 nmol/L, although in each case the heterogeneity remained high.

According to the intervention duration three systematic reviews conducted subgroup-analysis [37,42,51]. Two reviews reported no significant differences between trials with shorter or longer duration [42,51], while one systematic review reported that serum 25(OH)D concentrations increased more in studies with a trial duration between three to six months, as compared to those studies with an intervention duration shorter than three months or longer than six months [37].

Based on the AMSTAR tool [34], the quality was heterogeneous (1 to 8 potential sources of biases, from the 11 items of AMSTAR evaluation) among the systematic reviews investigating the effect of fortification with vitamin D on serum 25(OH)D concentrations (Table S4, Supplementary File S3). Six systematic reviews presented biases only in 1 or 2 fields of the AMSTAR tool [35,42,44,52,56,60], in contrast, four reviews had potential sources of biases in 7 or 8 fields of the AMSTAR tool [36,38,40,45].

#### 3.2.2. Effect of Vitamin D Fortification on Prevalence of Vitamin D Deficiency

One systematic review including 16 RCTs with a total number of 4093 healthy children reported a reduction in the prevalence of vitamin D deficiency after fortification of food (including milk, cereal, juice, bread, yogurt, and cheese) compared with no fortification (RR 0.53; 95% CI 0.41 to 0.69; *I*^2^ = 94%, GRADE: high certainty evidence) [60]. The number needed to treat (NNT) was calculated as 6.3 children to prevent one case of vitamin D deficiency [60]. The results of the individual studies are shown in Table 4. The systematic review had a low risk of bias, fulfilling 10 criteria out of 11 items of AMSTAR (Table S4, Supplementary File S3).

#### 3.2.3. Effect of Vitamin D Fortification on Parathormone and Bone Turnover Markers

Altogether, five systematic reviews reported the effect of food fortification with vitamin D on serum parathormone (PTH) levels and bone turnover markers [37,38,48,51,53]. Additionally, four reviews reported serum PTH levels, but not bone turnover markers [40,41,46,47]. The results of the meta-analysis are demonstrated in Table 5.

A recent systematic review (search date: 2020) focused on the effects of the vitamin D fortification of food products on bone biomarkers [51]. The duration of the intervention varied from one to thirty months, a part of included studies co-fortified with calcium (Ca), the dosage of vitamin D fortification varied from 40 IU/day to 28,000 IU/day, and dairy products were the dominant fortified foods used in most studies. The analysis of serum parathyroid hormone (PTH) in 25 reports showed a significant effect of the intervention (MD −5.148, 95% CI −7.341 to −2.955). Subgroup analysis according to age categorization indicated that the point estimates were similar in both older and younger than 18-year-old participants (−4.181 vs. −8.262, respectively). In the pooled analysis of eight reports, serum telopeptides of type-1 collagen (CTx) were significantly decreased in the intervention groups (MD −0.027, 95% CI −0.05 to −0.005). In contrast, serum osteocalcin (OC) did not alter significantly (MD 0.803, 95% CI: −0.65 to 2.255). Subgroup analysis revealed that the results were similar in adolescents and older populations. The duration of intervention was more than six months in only two studies, which did not indicate different results in comparison with studies of less than six months of interventions [51]. No significant increase was observed in the bone mineral density (BMD), with the exception of spine site areas (MD 0.081, 95% CI: 0.047 to 0.116).

Similar effects on PTH levels were reported in a few years earlier (search date: 2017) systematic review including 20 reports [37]. Sub-group analysis showed that serum PTH had more reduction if vitamin D was administered in a dose of >4000 IU/day as compared with doses of ≤400, 400–1000, and 1000–4000) [37]. They found no significant changes according to serum CTx levels or serum OC [37]. In contrast, the hip and spine BMD elevated significantly, and results remained significant if the dose of vitamin D was higher than 400 IU, the population was under the age of 35, the calcium dose was higher than 1000 mg/day, non-dairy fortified vehicles, and the effect seemed more effective at spine site areas [37]. Additionally, serum alkaline phosphatase (ALP) and amino-terminal pro-peptide of type 1 procollagen (P1NP) concentrations were investigated in this systematic review and no significant effects were seen as a result of food fortification with vitamin D [37].

One broadly focused systematic review, including all types of food fortificants and food vehicles, analyzed children, women of reproductive age (WRA), and post-menopausal women separately. In studies included in this systematic review, milk was the preferred food vehicle and the amount of micronutrient used varied significantly among the studies [53]. In children, vitamin D fortification significantly reduced serum PTH concentration (SMD −0.40, 95% CI −0.56 to −0.24), in WRA combined vitamin D and calcium had no impact on serum PTH levels (SMD −0.01, 95% CI −0.32 to 0.30). For post-menopausal women, a pooled analysis showed significant impacts on serum PTH concentration (SMD −2.53, 95% CI −4.42 to −0.65) [53]. Additionally, pooled analyses showed significantly reduced serum levels of P1NP (three studies; SMD of −3.36 (95% CI −6.37 to −0.35) and CTx (four studies; SMD of −4.93 (95% CI: −7.78 to −2.08) in both WRA and post-menopausal women [53].

A systematic review focusing on the fortification of yogurt with vitamin D also reported a significant decrease in PTH in the intervention compared with the control group (MD −15.47 ng/L, 95% CI −19.97 to −10.96; *I*^2^ = 93%) [47].

Among the systematic reviews analyzing the results narratively, a similar effect was observed on serum PTH levels and bone turnover markers [38,40,41,46,48]. Three systematic reviews reported significantly decreased levels of serum PTH in all of the included studies [41,46,48], in contrast, two systematic reviews found conflicting results [38,40]. Two systematic reviews summarized narratively the results on bone turnover markers [38,48]. In one systematic review the results were conflicting [48], the other review found no significant differences in serum OC, ALP, P1NP, or CTx [38].

These systematic reviews presented a risk of bias in 3 to 8 out of 11 items of AMSTAR (Table S4, Supplementary File S3).

#### 3.2.4. Effect of Vitamin D Fortification on Anthropometric Parameters

Two systematic reviews reported pooled results for anthropometric parameters [47,50]. The results of the meta-analyses can be found in Table 6.

One of the systematic reviews pooled the results of 15 studies using diverse food vehicles and found no effect of vitamin D fortification on weight (MD −0.065, 95% CI −0.439 to 0.309, *I*^2^: 88.5%) [50]. Subgroup analysis by duration of intervention showed that the intervention duration of ≤6 months was associated with a reduction in weight of the intervention group (MD −0.368, 95% CI −0.818 to 0.081). However, if the duration was more than six months, it was associated with a significant increase in weight (MD 0.904; 95% CI, 0.119 to 1.688) [50]. Vitamin D fortification reduced waist circumference (MD −1.283; 95% CI, −1.892 to −0.674) and waist-to-hip ratio (MD −0.020; 95% CI, −0.035 to −0.004), but its effects on BMI, fat mass, lean mass, and hip circumference were not significant [50].

The other systematic review, including studies on yogurt fortification with vitamin D, found a significant decrease in body weight (MD = −0.92 kg, 95% CI: −1.44 to −0.40, *I*^2^ = 99%, 7 studies, 589 participants), and waist circumference (MD −2.01 cm, 95% CI −2.56 to −1.47, *I*^2^ = 80%, 5 studies, 426 participants), but no significant change in BMI, and fat mass [47].

Both systematic reviews fulfilled 7 items out of 11 items of AMSTAR (Table S4, Supplementary File S3), presenting potential sources of biases.

#### 3.2.5. Effect of Vitamin D Fortification on Glucose Metabolism

Two systematic reviews—including both healthy and diabetic participants—reported on markers of glucose metabolism [47,49] and one reported on insulin growth factor-1 (IGF-1) quantitatively (Table 7) [51], and further two systematic reviews reported results narratively on glycemic status [39,40]. In the two systematic reviews with meta-analysis, most of the included studies were conducted in diabetic participants, therefore findings may not be generalizable to healthy populations [47,49]. Pooled estimates showed no change in hemoglobin A1c (HbA1c) levels after vitamin D fortification, but indicated a decrease in fasting serum glucose (FSG), fasting serum insulin (FSI), and HOMA-IR (Homeostatic Model Assessment for Insulin Resistance) and an increase in insulin growth factor-1 (IGF-1). These systematic reviews fulfilled 7 to 8 fields out of 11 items of AMSTAR tool (Table S4, Supplementary File S3).

According to the narrative results in one systematic review, the glycemic status was improved in diabetic patients (risk of bias: in 8 out of 11 items of AMSTAR—Table S4, Supplementary File S3) [40], the other review reported one study with no changes in fasting serum glucose (risk of bias: in 3 items out of 11 in AMSTAR evaluation—Table S4, Supplementary File S3) [39].

#### 3.2.6. Effect of Vitamin D Fortification on Lipid Levels

One systematic review, including studies with vitamin D-fortified yogurt, summarized the results of five studies, including 469 participants on lipid profiles [47]. The meta-analysis showed a significant decrease in total cholesterol (MD −13.38 mg/dL, 95% CI −20.19 to −6.56, *I*^2^ = 98%) and triglycerides (MD −30.12 mg/dL, 95% CI −43.22 to −17.02, *I*^2^ = 95%). There was also a decrease in low-density lipoprotein (LDL) cholesterol (MD −7.86 mg/dL, 95% CI −15.35 to −0.37, *I*^2^ = 99%) in intervention groups compared with control groups, while the increase in high-density lipoprotein (HDL) cholesterol was not significant (Table 8). The systematic review presented a risk of bias in 4 out of 11 items of AMSTAR (Table S4, Supplementary File S3).

#### 3.2.7. Effect of Vitamin D Fortification on Serum Calcium and Other Adverse Effects

Three systematic reviews reported results on serum calcium levels [38,40,53]. One systematic review summarizing the results of 7 trials conducted on children reported a significant decrease in serum calcium levels (SMD −0.40, 95% CI: −0.59 to −0.20) [53]. Another systematic review reported results on four trials and narratively found that in three included trials the serum calcium concentration remained stable, in one study decreased, but only in the fortified rye bread group [40]. The third systematic review included studies with fortified bread and only reported that the serum calcium levels remained stable in the fortification studies [38]. The number of items presenting a risk of bias ranged from 4 to 8 of 11 in the AMSTAR tool (Table S4, Supplementary File S3).

A further four systematic reviews assessed other adverse effects of fortification (risk of bias: in 1 to 5 items out of 11 in AMSTAR evaluation—Table S4, Supplementary File S3) [35,41,55,58]. One study investigated the effect of food fortification with vitamin D in children and reported most children reached or maintained sufficiency status with no concentrations reaching the toxic threshold (25(OH)D > 250 nmol/L) [58]. Two systematic reviews reported no adverse effects of fortification [35,55]. One systematic review summarizing the results of three trials with 434 participants reported eight dropouts from the intervention group because of gastrointestinal side effects [41].

#### 3.2.8. Other Reported Outcomes

One systematic review investigated the cost-effectiveness of food fortification and supplementation with vitamin D, and found that vitamin D-fortified bread was cost-saving based on fracture cost (risk of bias: in 5 fields out of 11 in AMSTAR evaluation—Table S4, Supplementary File S3) [61].

Another systematic review included school performance (2 trials, 904 participants), cognitive function (2 trials), and infection rate (2 trials) as secondary outcomes (low risk of bias based on AMSTAR evaluation—Table S4, Supplementary File S3) [60]

A third systematic review investigated the relationship between vitamin D fortification and cancer mortality [45]. Based on three studies, the authors stated: “Fortification with 400 IU, 800 IU, and 2000 IU vit D/day found cancer mortality reductions by 11%, 15%, and 17%, respectively” [45].

### 3.3. Overview of the Individual Studies That Formed the Basis of the Systematic Reviews and the Resulting Overview of Reviews

A total of 115 primary studies were included in the 27 systematic reviews. A table mapping the primary vitamin D fortification studies included in the 27 systematic reviews is shown in Supplementary File S5 [47,62,63,64,65,66,67,68,69,70,71,72,73,74,75,76,77,78,79,80,81,82,83,84,85,86,87,88,89,90,91,92,93,94,95,96,97,98,99,100,101,102,103,104,105,106,107,108,109,110,111,112,113,114,115,116,117,118,119,120,121,122,123,124,125,126,127,128,129,130,131,132,133,134,135,136,137,138,139,140,141,142,143,144,145,146,147,148,149,150,151,152,153,154,155,156,157,158,159,160,161,162,163,164,165,166,167,168,169,170,171,172,173,174,175]. The studies were included in one to eight systematic reviews (mean: 2.72). A total of 46 primary studies were included in only one systematic review, while the remaining 69 primary studies were included in multiple systematic reviews.

Most individual studies investigated the effects of vitamin D fortification of milk (Supplementary File S6) or other dairy products (Supplementary File S7). Among them, the most frequent outcome investigated was serum 25(OH)D. Additionally, there were a lower number of individual studies providing vitamin D-fortified buns or bread (Supplementary File S8), juice or beverages (Supplementary File S9), cereal, biscuits, or snack bars (Supplementary File S10), multiple food items (Supplementary File S11), oil or biofortified food products (Supplementary File S12) to the participants.

## 4. Discussion

This overview summarizes 27 systematic reviews carried out between 2006 and 2022, including 2 to 40 primary vitamin D fortification trials. General populations from diverse age groups were included. In the included primary studies, dairy products and bread were the most frequently fortified foods, with daily vitamin D fortification dosages ranging from 60 to 5000 IU. Based on currently available evidence, fortification of food with vitamin D results in a reduction of vitamin D deficiency and might increase serum 25(OH)D concentrations, to varying degrees, depending on the fortified vehicle, fortification dosage, and characteristics of the population. Fortification of food with vitamin D may have a positive impact on bone turnover and may improve some lipid parameters, but may have only limited effect on anthropometric parameters. The methodological quality of the reviews was assessed using AMSTAR, with most demonstrating moderate to high quality.

The main results of the overview demonstrate a positive impact of vitamin D fortification on serum 25(OH)D concentrations and the reduction of vitamin D deficiency. One study investigating the effect of the systematic voluntary vitamin D fortification policy in Finland between 2000 and 2011 reported improved vitamin D status, which was mostly explained by the fortification (mainly from milk), but supplementation and changes in UV radiation during this time also contributed to this result [109].

Several reports discussed the effectiveness of vitamin D food fortification as a strategy to address vitamin D deficiency across diverse populations [176,177,178,179]. Countries like the US, Canada, India, and Finland have already embraced this strategy, leading to improvements in vitamin D status among their citizens [176,177]. Notably, Southeast Asian countries have also explored the potential benefits of fortifying widely consumed foods, such as edible oil, to address vitamin D deficiency in their populations [178]. Furthermore, investigations in Mongolia have revealed that fortification of staple foods, including flour, milk, and edible oils, with vitamin D3 could significantly raise 25(OH)D concentrations, indicating its potential impact on public health [179]. This alignment with existing research supports the notion that fortification can effectively increase vitamin D concentrations in the body, making it a valuable strategy for addressing the widespread issue of vitamin D deficiency.

One strength of our overview of reviews is that it includes numerous systematic re-views identified using systematic searches in four databases. Additionally, there were no restrictions on the outcomes investigated, ensuring a comprehensive representation of results. We were able to include several systematic reviews investigating the effects of food fortification with vitamin D on serum 25(OH)D concentrations. The high heterogeneity of these results calls attention to the role of certain modifying factors, such as the type of vehicle, the dose of vitamin D administered, the type of vitamin D, the baseline serum 25(OH)D level, the population characteristics, or the difference in settings. Only a small number of the included systematic reviews analyzed these modifying factors separately or as a subgroup analysis, limiting the appropriate evaluation of these factors.

We aimed to assess the benefits or harms of fortification with vitamin D, but the low number of studies assessing the potential adverse effects of vitamin D fortification limited our ability to properly assess the potential harms of this intervention. We found only limited evidence regarding several important outcomes, e.g., bone turnover markers, anthropometric parameters, blood lipid levels, and cancer mortality, making it difficult to comprehensively assess the effect of vitamin D fortification on these outcomes.

When analyzing the primary studies included in the systematic reviews, we found that a large part of the evidence focused on the fortification of milk or dairy products. Some of the included primary studies dealt with the fortification of bread and juice, but only a few of the included primary studies dealt with the fortification of other types of food, such as snacks or oils, limiting the possibility of appropriately assessing their effectiveness.

We collected data on the certainty of evidence in the included systematic reviews and the risk of bias of primary studies to assess the factors that could impact the validity of the results. Although almost all systematic reviews reported the risk of bias assessment of the included primary studies, only two systematic reviews reported GRADE results. We assessed the methodological quality of the included systematic reviews using AMSTAR [34]. The majority of studies demonstrated moderate-to-high-quality methodological rigor and adherence to best practices in conducting systematic reviews. However, it should be considered that although AMSTAR is a valid and feasible measurement tool recommended for the overview of reviews [29], the quality of reporting may modify some items of the AMSTAR tool [180], making the methodological quality dependable on the adequate reporting of the findings as well.

The positive impact of food fortification with vitamin D on serum 25(OH)D concentrations and vitamin D deficiency prevalence highlights that implementing or expanding fortification programs to address vitamin D deficiencies in populations might have positive effects. Collaboration with food manufacturers could be important in the implementation process and might help establish clear guidelines and regulations for the fortification process. Local settings (e.g., latitude, climate), population characteristics (e.g., baseline vitamin D status), dietary vitamin D intake, and factors influencing vitamin D synthesis in the skin (e.g., skin type, clothing habits) at the populational level should be considered when establishing regulations. To maintain the safety and effectiveness of vitamin D fortification efforts, it is crucial to identify appropriate fortification dosages and ensure the consumption of these fortified foods. A modeling study investigating the effect of food fortification with vitamin D (milk, plant-based oil, wheat flour) in seven low/lower-middle income countries with high vitamin D deficiency prevalence found that only wheat flour fortified with 400 IU vitamin D/100 g could theoretically increase the vitamin D intake >200 IU/day in all of these low/lower-middle income countries [181]. By establishing clear guidelines, policymakers and stakeholders can facilitate the successful implementation of vitamin D fortification programs, ensuring that the public receives the intended benefits while minimizing the potential risks associated with inappropriate dosages.

This overview of reviews focused on the generally healthy population, but fortification at the populational level affects individuals with specific diseases as well, which should be considered. Based on the results of this overview of reviews, we were able to identify numerous research gaps. There is still limited knowledge about the effects of vitamin D fortification on important health outcomes, such as PTH levels, bone mineral density, glucose metabolism, lipid levels, and anthropometric measures. To gain a more comprehensive understanding of the overall impact of vitamin D fortification on health, future researchers should prioritize investigating these aspects.

For better comparability of different vehicles, future studies should focus on vehicles other than dairy products or bread. Researchers should consider stratified analyses based on the different types of vitamin D used for fortification to determine whether specific types lead to better vitamin D outcomes. Systematic reviews summarizing evidence on vitamin D fortification should follow a more rigorous methodology.

## 5. Conclusions

Results of this overview of reviews indicate a positive impact of vitamin D fortification of food on serum 25(OH)D concentrations and the reduction of vitamin D deficiency. While these findings are promising, further research exploring the broader health effects and potential harms of vitamin D fortification and optimal fortification dosages for the different food vehicles fortified are necessary to effectively address vitamin D deficiency and its associated health risks.

## Figures and Tables

**Table 1:** Baseline characteristics of the included systematic reviews.

Review (First Author, Year)	Number of Studies	Number of Participants	Included Participants	Eligible Fortified Food Product	Vit D Compound Used for Fortification	Duration of Intervention	Dose of Fortification	Outcome
Aguiar 2017 [61]	14 studies	Not reported	General population of all ages	Food	Not specified	Not reported	800 IU/day	Fractures, cost per avoided fractures
Al Khalifah 2020 [60]	20 RCTs	*n* = 5358	Children	Food	Vitamin D3 or not specified	2–24 months	60–1000 IU/day	Serum 25(OH)D, vitamin D deficiency prevalence, school performance, cognitive function, infection rate, hospital admission length
Brandão-Lima 2019 [58]	5 RCTs	*n* = 792; intervention: *n* = 568, control: *n* = 224	Children: ages 2–11 years, both sexes	Food	Vitamin D2 and D3	1.6–9 months	42–880 IU/serving, 300–880 IU/day	Serum 25(OH)D, harm
Black 2012 [59]	15 RCTs	*n* = 1523	Adults	Food	Not specified	11 weeks–2 years	120–1000 IU/100 g or serving	Serum 25(OH)D
Brett 2018 [57]	26 RCT	*n* = 5403	Healthy children aged 2–18 years	Food	Vitamin D3 or not reported	1.6 month–2 years	100–1000 IU/day	Serum 25(OH)D
Brooker 2022 [56]	12 RCTs	*n* = 4795	Healthy Children Aged 9–48 Months	Milk or milk formula	Not specified	20 weeks–12 months	The dose of milk: 150–750 mL/day	Serum 25(OH)D
Cashman 2021 [54]	11 RCTs	*n* = 1429	Children and adults, both sexes	Food	Vitamin D3	8 weeks–6 months	140–4000 IU/day	Serum 25(OH)D
Cranney 2007 [35]	13 RCTs	*n* = 1281, intervention: *n* = 697, control: *n* = 584	General population of all ages	Food	Vitamin D3 or not specified	3 weeks–24 months	228–800 IU/day	Serum 25(OH)D
Cranney 2008 [55]	11 RCTs	Not reported	General population of all ages	Food	Vitamin D3 or not specified	Not reported	137–1000 IU/day	Serum 25(OH)D, adverse effects
Das 2013 [53]	Children: 7 RCTs, 1 CCT, 2 before-after studies	Not reported	Children and adolescents, age: 2 to 18 years	Milk	Not specified	Not reported	Not reported	Serum 25(OH)D, PTH, Ca, P1NP, CTx
	Women: 13 RCTs, 1 before-after study	Not reported	Women (of reproductive age and post-menopausal)	Food	Not specified	2 weeks–2 years		
Dunlop 2021 [52]	34 RCTs	*n* = 3930, intervention: *n* = 2315, control: *n* = 1615	Children and adults, without compromised vitamin D absorption	Food	vitamin D2 or D3, or not specified	4–104 weeks	200–4000 IU/day	Serum 25(OH)D
Emadzadeh 2022 [51]	40 RCTs	Not reported	General population of all ages	Food	Not specified	1–30 months	40–28,000 IU/day	Serum 25(OH)D, PTH, IGF-1, CTx, OC, BMD
Emadzadeh 2020a [50]	20 RCTs	*n* = 2297, intervention: *n* = 1146, control: *n* = 1151	General population of all ages	Food	Vitamin D3 or not specified	2–24 months	100–28,000 IU/day	Weight, BMI, FM, LM, WC, HC, WHR
Emadzadeh 2020b [49]	11 RCTs	*n* = 1070, intervention: *n* = 532, control: *n* = 538	General population of all ages	Food	Vitamin D3	2–6 months	Range: 1000–28,000 IU/day	FSG, FSI, HOMA-IR, HbA1c
Fonseca Santos 2022 [48]	5 RCTs	Not reported	Postmenopausal women	Food	Vitamin D3	4–12 weeks.	50–200 IU/serving	Serum 25(OH)D, PTH, CTx, TRAP5b, P1NP
Gasparri 2019 [47]	9 studies	*n* = 665, intervention *n* = 322, control *n* = 343	Women and men aged 18 to 99 years	Yogurt	Not specified	8–16 weeks	400–2000 IU/day	Serum 25(OH)D, PTH, weight, BMI, FM, WC, TC, TG, LDL, HDL, HOMA-IR, FSG, BP
Lam 2016 [46]	5 studies	*n* = 181	People living in residential care	Food	Not specified	1–12 months	100–5000 IU/day	Serum 25(OH)D, PTH
Niedermaier 2021 [45]	10 articles	RCT: *n* = 1303, pre-post design: *n* = 6134 and 4051, meta-analysis *n* = 665	General population of all ages	Food	Not specified	8 weeks–11 years	200–1040 IU/day	Serum 25(OH)D, cancer mortality
Nikooyeh 2018 [43]	5 studies	*n* = 189	Iranian adult participants	Food	Not specified	8–12 weeks	1000–2000 IU/day	Serum 25(OH)D
Nikooyeh 2022a [44]	31 studies	*n* = 7593, intervention: *n* = 4583, control: *n* = 3010	Children aged 1 to 18 years	Food	Not specified	1–12 months	80–1000 IU/day	Serum 25(OH)D
Nikooyeh 2022b [42]	23 studies	*n* = 2002, intervention: *n* = 1173, control: *n* = 829	Adults aged 18 years and older	Food	Vitamin D2 or D3	3 weeks–2 years	200–2000 IU/day	Serum 25(OH)D
O’Donnell 2008 [41]	9 RCTs	*n* = 889, intervention: *n* = 437, control: *n* = 452)	All populations, community-dwelling participants	Food	Vitamin D3	3 weeks–24 months	136–1000 IU/day	Serum 25(OH)D, PTH, harm
O’Mahony 2011 [40]	9 studies	*n* = 850	General population of all ages	Food	Vitamin D2 or D3	3–12 weeks,	400–4000 IU/day	Serum 25(OH)D, PTH, Ca, glycemic status
Soto-Mendez 2019 [39]	41 RCTs (2 on fortification with vitamin D)	fortification: *n* = 262, intervention *n* = 104	General population of all ages	Milk or dairy products	Vitamin D3	16 weeks	200–500 IU/day	TC, HDL, LDL, TG, BP, glucose
Souza 2022 [38]	20 articles, including 10 Clinical trial studies	Not reported	General population of all ages	Bread	Vitamin D2 or D3	3 weeks–12 months	172–5000 IU/100 g	Serum 25(OH)D, PTH, Ca, OC, ALP, P1NP, CTx
Tangestani 2020 [37]	20 trials	*n* = 1786	Healthy population, without age restriction,	Food	Not specified	1–24 months	80–5000 IU/day	Serum 25(OH)D, BMD, PTH, OC, ALP, CTx, P1NP
Whiting 2015 [36]	18 publications (1 with fortification)	Not reported	Healthy adults	Food	vitamin D3	8 weeks	400 IU/day	Serum 25(OH)D

ALP: alkaline phosphatase, BMD: Bone mineral density, BMI: Body mass index, BP: blood pressure, Ca: calcium, CCT: controlled clinical trial, CTx: serum telopeptides of type-1 collagen, FM: fat mass, FSG: fasting serum glucose, FSI: fasting serum insulin, HbA1c: hemoglobin A1c, HC: hip circumference, HDL: high-density lipoprotein, HOMA-IR: Homeostatic Model Assessment for Insulin Resistance, IGF-1: insulin growth factor-1, IU: International unit, LDL: low-density lipoprotein, LM: lean mass, OC: osteocalcin, PTH: parathormone, P1NP: Total procollagen type 1 N-terminal propeptide, RCT: Randomized controlled trial, TC: total cholesterol, TG: triglyceride, TRAP5b: tartrate resistant acid phosphatase 5b, WC: waist circumference, WHR: waist-to-hip ratio, 25(OH)D: 25-Hydroxy vitamin D.

**Table 2:** Results of included systematic reviews with meta-analysis on serum 25(OH)D.

Review (First Author, Year	Number of Included Trials	Trial Designs Included	Eligible Vehicle	Vehicle in Included Studies	Effect of Vitamin D Fortification	Direction of Effect
Al Khalifah 2020 [60]	20	RCT	food	milk	MD 23.72 nmol/L (95% CI 22.86 to 24.58)	↑
				juice	MD 11.80 nmol/L (95% CI 7.35 to 16.26)	↑
				cereal	MD 8.93 nmol/L (95% CI −0.36 to 18.21)	–
				yogurt and cheese	MD 5.34 nmol/L (95% CI 0.97 to 9.70)	↑
Black 2012 [59]	15	RCT	food	dairy products, orange juice, bread	MD 19.4 nmol/L (95% CI 13.9 to 24.9)	↑
Brett 2018 [57]	7	RCT	food	milk, yogurt/cheese, cereal-based food, bread	MD 6.9 nmol/L (95% CI 3.7 to 10.0)	↑
Das 2013 [53]	24	RCT + NRSI	food	milk	children SMD 1.23 nmol/L (95% CI 0.35 to 2.11)	↑
				NR	women SMD −1.10 nmol/L (95% CI −3.81 to 1.60)	–
Dunlop 2021 [52]	34	RCT	food	milk, milk powder, milk-based drinks, yogurt, cheese, fruit juice, biscuits, snack bars, bread	MD 21.2 nmol/L (95% CI 16.2 to 26.2)	↑
Emadzadeh 2022 [51]	40	RCT	food	dairy products	MD 16.52 nmol/L (95% CI 11.62 to 21.42)	↑
Gasparri 2019 [47]	9	RCT	yogurt	yogurt	MD 31.00 nmol/L (95% CI 26.10 to 35.91)	↑
Nikooyeh 2018 [43]	5	RCT	food	milk, yogurt, yogurt drink, bread	MD 34.68 nmol/L (95% CI 28.59 to 40.77)	↑
Nikooyeh 2022a [44]	11	RCT	food	dairy products	MD 20.29 nmol/L (95% CI 13.32 to 27.25)	↑
Nikooyeh 2022b [42]	23	RCT	food	dairy products, grain products, juice, oil and dairy with grain products	MD 25.40 nmol/L (95% CI 19.50 to 31.30)	↑
O’Donnell 2008 [41]	4	RCT	food	milk	MD 15.63 nmol/L (95% CI 12.79 to 18.48)	↑
Tangestani 2020 [37]	20	RCT + NRSI	food	milk, yogurt, yogurt drink, cheese, orange juice, bread	MD 16.94 nmol/L (95% CI 13.38 to 20.50)	↑

MD: mean difference, NRSI: non-randomized studies of interventions, RCT: Randomized controlled trial, SMD: Standardized Mean Difference, 95% CI: 95% confidence interval.

**Table 3:** Mean change of serum 25(OH)D concentrations/100 IU vitamin D administered.

Author	Number of Studies	Fortified Vehicle	Specific Population	Mean Change in Se 25(OH)D (nmol/L)/100 IU Vitamin D Administered
Al Khalifah, 2020 [60]	18	cereal, milk, dairy products, bread, juice, two items of food: yogurt and cheese or milk and bread	children	3
Black, 2012 [59]	7	dairy products, juice, bread	adults	3
Brett, 2018 [57]	7	cereal, milk, dairy products, bread, juice	healthy children	6.9
	4		baseline vitamin D status <50 nmol/L	4.2–10.8
Cashman, 2021 [54]	11	milk, dairy products, bread, eggs, orange juice, milk + bread, cheese + Yogurt + eggs + crisp bread		4
	3	milk, dairy products	children	4.75
	8	milk, dairy products, bread, eggs, orange juice, milk + bread, cheese + Yogurt + eggs + crisp bread	adults	6.5
Cranney, 2008 [55]	11	dairy products		1–2
Nikooyeh, 2018 [43]	5	yogurt, yogurt drink, milk, bread	Iranian adult participants	3.5
Nikooyeh, 2022 [44]	11	dairy products, juice, grain products	children	0.7
Nikooyeh, 2022 [42]	23	dairy products, juice, grain product, oil and dairy together with grain products	adult	2
Whiting, 2015 [36]	1	yogurt		5.05

**Table 4:** Effect of fortification with vitamin D on Vitamin D deficiency prevalence (Results of individual studies).

Study	Food Vehicle	Risk Ratio (95% CI)	Direction of Effect
Akkermans et al., 2017 [62]	Milk	0.41 (0.23–0.72)	↓
Benjeddou et al., 2019 [63]	Milk	0.45 (0.24–0.84)	↓
Brett 2018 [64]	Yogurt and cheese	1.77 (0.17–18.26)	–
Brett et al., 2016 [65]	Yogurt and cheese	0.11 (0.03–0.49)	↓
Economos et al., 2014 [66]	Juice	2.06 (0.24–17.96)	–
Graham et al., 2009 [67]	Milk	0.48 (0.29–0.78)	↓
Houghton et al., 2011 [68]	Milk	0.80 (0.66–0.96)	↓
Hower et al., 2013 [69]	Milk	0.77 (0.14–4.21)	–
Khadgawat et al., 2013 [70]	Milk	0.51 (0.46–0.56)	↓
Kuriyan et al., 2016 [71]	Malt- and cocoa-based milk	1.03 (0.34–3.09)	–
Madsen et al., 2013 [72]	Bread and milk	0.26 (0.15–0.44)	↓
Neyestani et al., 2014 [73]	Milk	0.93 (0.83–1.05)	–
Neyestani et al., 2014 [73]	Orange juice	0.93 (0.86–1.00)	–
Ohlund et al., 2017 [74]	Milk	0.28 (0.17–0.46)	↓
Powers et al., 2016 [75]	Cereal and milk	0.64 (0.34–1.02)	–
Rich-Edwards et al., 2011 [76]	Milk	0.27 (0.22–0.33)	↓
Wang et al., 2017 [77]	Milk	0.87 (0.31–2.45)	–

**Table 5:** Results of included systematic reviews with meta-analysis on bone turnover markers.

Review (First Author, Year	Number of Included Trials	Trial Designs Included	Eligible Vehicle	Vehicle in Included Studies	Effect of Vitamin D Fortification	Direction of Effect	GRADE
**Parathyroid hormone (PTH)**							
Das 2013 [53]	7	RCT + NRSI	food	milk	children SMD −0.40 (95% CI −0.56 to −0.24)	↓	⊕⊕OO LOW
	13	RCT + NRSI	food	NR	women of reproductive age SMD −0.01 (95% CI −0.32 to 0.30)	–	⊕⊕OO LOW
			food	NR	post-menopausal women SMD −2.53 (95% CI −4.42 to −0.65)	↓	⊕⊕OO LOW
Emadzadeh 2022 [51]	25	RCT	food	dairy products	MD −5.15 (95% CI −7.34 to −2.96)	↓	not reported
Gasparri 2019 [47]	4	RCT	yogurt	yogurt	MD −15.47 ng/L (95% CI −19.97 to −10.96)	↓	not reported
Tangestani 2020 [37]	15	RCT + NRSI	food	milk, yogurt, yogurt drink, cheese, orange juice, bread	MD −9.22 ug/L (95% CI −14.97 to −3.46)	↓	not reported
Serum ALP							
Tangestani 2020 [37]	8	RCT + NRSI	food	milk, yogurt, yogurt drink, cheese, orange juice, bread	MD −3.434 ug/L (95% CI −7.959 to 1.090)	–	not reported
Serum CTx							
Das 2013 [53]	4	RCT + NRSI	food	NR	SMD −4.93 (95% CI −7.78 to −2.08)	↓	not reported
Emadzadeh 2022 [51]	8	RCT	food	dairy products	MD −0.027 (95% CI −0.05 to −0.005)	↓	not reported
Tangestani 2020 [37]	4	RCT + NRSI	food	milk, yogurt, yogurt drink, cheese, orange juice, bread	MD −0.06 mg/L (95% CI −0.15 to 0.03)	–	not reported
	2				MD −0.307 mg/L (95% CI −1.07 to 0.46)	–	not reported
Serum Ca							
Das 2013 [53]	7	RCT + NRSI	food	NR	SMD −0.40 (95% CI −0.59 to −0.20)	↓	⊕⊕OO LOW
**Bone mineral density (BMD)**							
Emadzadeh 2022 [51]		RCT	food	dairy products	MD 0.081 g/cm^2^ (95% CI 0.047 to 0.116)	↑	not reported
Tangestani 2020 [37]	6	RCT + NRSI	food	milk, yogurt, yogurt drink, cheese, orange juice, bread	MD 0.03 g/cm^2^ (95% CI 0.02 to 0.05)	↑	not reported

ALP: alkaline phosphatase, BMD: Bone mineral density, Ca: calcium, CTx: serum telopeptides of type-1 collagen, GRADE: Grading of Recommendations, Assessment, Development, and Evaluations, MD: mean difference, NRSI: non-randomized studies of interventions, PTH: parathormone, RCT: Randomized controlled trial, SMD: Standardized Mean Difference, 95% CI: 95% confidence interval.

**Table 6:** Results of included systematic reviews with meta-analysis on anthropometric parameters.

Review (First Author, Year	Number of Included Trials	Trial Designs Included	Eligible Vehicle	Vehicle in Included Studies	Effect of Vitamin D Fortification	Direction of Effect	GRADE
**Weight**							
Emadzadeh 2020 [50]	15	RCT	food	milk, yogurt, cheese, orange juice, bread, eggs, snack bar	MD −0.07 kg (95% CI −0.44 to 0.31)	–	not reported
Gasparri 2019 [47]	7	RCT	yogurt	yogurt	MD −0.92 kg (95% CI −1.44 to −0.40)	↓	not reported
**BMI**							
Emadzadeh 2020 [50]	16	RCT	food	milk, yogurt, cheese, orange juice, bread, eggs, snack bar	MD −0.044 kg/m^2^ (95% CI −0.229 to 0.142)	–	not reported
Gasparri 2019 [47]	6	RCT	yogurt	yogurt	MD −0.15 kg/m^2^ (95% CI −0.33 to 0.03)	–	not reported
**Fat mass**							
Emadzadeh 2020 [50]	10	RCT	food	milk, yogurt, cheese, orange juice, bread, eggs, snack bar	MD −0.542% (95% CI −1.207 to 0.123)	–	not reported
Gasparri 2019 [47]	6	RCT	yogurt	yogurt	MD −1.3% (95% CI −2.95 to 0.35)	–	not reported
**Lean mass**							
Emadzadeh 2020 [50]	3	RCT	food	milk, yogurt, cheese, orange juice, bread, eggs, snack bar	MD −0.089 (95% CI −0.496 to 0.317)	–	not reported
**Waist circumference**							
Emadzadeh 2020 [50]	6	RCT	food	milk, yogurt, cheese, orange juice, bread, eggs, snack bar	MD −1.283 cm (95% CI −1.892 to −0.674)	↓	not reported
Gasparri 2019 [47]	5	RCT	yogurt	yogurt	MD −2.01 cm (95% CI −2.56 to −1.47)	↓	not reported
**Hip circumference**							
Emadzadeh 2020 [50]	3	RCT	food	milk, yogurt, cheese, orange juice, bread, eggs, snack bar	MD −0.127 cm (95% CI −0.842 to 0.589)	–	not reported
**Waist-to-hip ratio**							
Emadzadeh 2020 [50]	5	RCT	food	milk, yogurt, cheese, orange juice, bread, eggs, snack bar	MD −0.020 (95% CI −0.035 to −0.004)	↓	not reported

GRADE: Grading of Recommendations, Assessment, Development, and Evaluations, MD: mean difference, RCT: Randomized controlled trial, 95% CI: 95% confidence interval.

**Table 7:** Results of included systematic reviews with meta-analysis on glucose metabolism.

Review (First Author, Year	Number of Included Trials	Trial Designs Included	Eligible Vehicle	Vehicle in Included Studies	Effect of Vitamin D Fortification	Direction of Effect	GRADE
**HbA1c**							
Emadzadeh 2020 [49]	17	RCT	food	milk, yogurt, cheese, orange juice, bread, eggs, snack bar	MD 0.034 (95% CI −0.655 to 0.069)	–	not reported
**Fasting serum glucose (FSG)**							
Emadzadeh 2020 [49]	11	RCT	food	milk, yogurt, cheese, orange juice, bread, eggs, snack bar	MD −2.772 (95% CI −5.435 to −0.109)	↓	not reported
Gasparri 2019 [47]	4	RCT	yogurt	yogurt	MD −22.54 mg/dL (95% CI −37.55 to −7.52)	↓	not reported
**Fasting serum insulin (FSI)**							
Emadzadeh 2020 [49]	9	RCT	food	milk, yogurt, cheese, orange juice, bread, eggs, snack bar	MD −2.937 (95% CI −4.695 to −1.178)	↓	not reported
**HOMA-IR**							
Emadzadeh 2020 [49]	5	RCT	food	milk, yogurt, cheese, orange juice, bread, eggs, snack bar	MD −1.608 (95% CI −3.138 to −0.079)	↓	not reported
Gasparri 2019 [47]	4	RCT	yogurt	yogurt	MD −2.18 (95% CI −2.92 to −1.44)	↓	not reported
**Insulin Growth Factor-1 (IGF-1)**							
Emadzadeh 2022 [51]	8	RCT	food	dairy products	MD 42.789 (95% CI 14.607 to 70.971)	↑	not reported

FSG: fasting serum glucose, FSI: fasting serum insulin, GRADE: Grading of Recommendations, Assessment, Development, and Evaluations, HbA1c: hemoglobin A1c, HOMA-IR: Homeostatic Model Assessment for Insulin Resistance, IGF-1: insulin growth factor-1, MD: mean difference, RCT: Randomized controlled trial, 95% CI: 95% confidence interval.

**Table 8:** Results of included systematic reviews with meta-analysis on serum lipid levels.

Review (First Author, Year	Number of Included Trials	Trial Designs Included	Eligible Vehicle	Vehicle in Included Studies	Effect of Vitamin D Fortification	Direction of Effect	GRADE
**Total cholesterol (TC)**							
Gasparri 2019 [47]	5	RCT	yogurt	yogurt	MD −13.38 mg/dL (95% CI −20.19 to −6.56)	↓	not reported
**Low density lipoprotein (LDL)**							
Gasparri 2019 [47]	5	RCT	yogurt	yogurt	MD −7.86 mg/dL (95% CI −15.35 to −0.37)	↓	not reported
**High density lipoprotein (HDL)**							
Gasparri 2019 [47]	5	RCT	yogurt	yogurt	MD 1.48 mg/dL (95% CI −0.18 to 3.13)	–	not reported
**Triglyceride (TG)**							
Gasparri 2019 [47]	5	RCT	yogurt	yogurt	MD −30.12 mg/dL (95% CI −43.22 to −17.12)	↓	not reported

GRADE: Grading of Recommendations, Assessment, Development, and Evaluations, HDL: high-density lipoprotein, LDL: low-density lipoprotein, MD: mean difference, RCT: Randomized controlled trial, TC: total cholesterol, TG: triglyceride, 95% CI: 95% confidence interval.

## Data Availability

All data generated or analyzed during this study are included in this published article (and its Supplementary Files).

## Supplementary Materials

The following supporting information can be downloaded at: https://www.mdpi.com/article/10.3390/nu15173742/s1, File S1: PRIOR checklist; File S2: Search Strategy; File S3: Table S1–S5 Characteristics of the included studies; File S4: Excluded studies; File S5: Primary studies included in the systematic reviews; File S6: Outcomes investigated in studies on vitamin D fortification of milk and milk powder; File S7: Outcomes investigated in studies on vitamin D fortification of dairy product; File S8: Outcomes investigated in studies on vitamin D fortification of bun and bread; File S9: Outcomes investigated in studies on vitamin D fortification of juice and beverages; File S10: Outcomes investigated in studies on vitamin D fortification of cereal biscuits and snack bars; File S11: Outcomes investigated in studies on vitamin D fortification of multiple food items; File S12: Outcomes investigated in studies on vitamin D fortification of oils and biofortification of eggs and mushrooms.

## References

[1] Holick M.F., Chen T.C. (2008). Vitamin D deficiency: A worldwide problem with health consequences. Am. J. Clin. Nutr..

[2] Charoenngam N., Shirvani A., Holick M.F. (2019). Vitamin D for skeletal and non-skeletal health: What we should know. J. Clin. Orthop. Trauma.

[3] Charoenngam N., Holick M.F. (2020). Immunologic Effects of Vitamin D on Human Health and Disease. Nutrients.

[4] Peters B.S.E., Martini L.A. (2010). Nutritional aspects of the prevention and treatment of osteoporosis. Arq. Bras. De Endocrinol. Metabol..

[5] DeLuca H.F. (2004). Overview of general physiologic features and functions of vitamin D. Am. J. Clin. Nutr..

[6] Lips P., van Schoor N.M., De Jongh R.T. (2014). Diet, sun, and lifestyle as determinants of vitamin D status. Ann. N. Y. Acad. Sci..

[7] Cashman K.D., Dowling K.G., Škrabáková Z., Gonzalez-Gross M., Valtueña J., De Henauw S., Moreno L., Damsgaard C.T., Michaelsen K.F., Mølgaard C. (2016). Vitamin D deficiency in Europe: Pandemic?. Am. J. Clin. Nutr..

[8] Wacker M., Holick M.F. (2013). Sunlight and Vitamin D: A global perspective for health. Derm. -Endocrinol..

[9] Mithal A., Wahl D.A., Bonjour J.P., Burckhardt P., Dawson-Hughes B., Eisman J.A., El-Hajj Fuleihan G., Josse R.G., Lips P., Morales-Torres J. (2009). Global vitamin D status and determinants of hypovitaminosis D. Osteoporos. Int..

[10] Holick M.F., Binkley N.C., Bischoff-Ferrari H.A., Gordon C.M., Hanley D.A., Heaney R.P., Murad M.H., Weaver C.M. (2011). Evaluation, Treatment, and Prevention of Vitamin D Deficiency: An Endocrine Society Clinical Practice Guideline. Med. J. Clin. Endocrinol. Metab..

[11] Del Valle H., Yaktine A., Taylor C., Ross A. (2011). Dietary Reference Intakes for Calcium and Vitamin D.

[12] Roth D.E., Abrams S.A., Aloia J., Bergeron G., Bourassa M.W., Brown K.H., Calvo M.S., Cashman K.D., Combs G., De-Regil L.M. (2018). Global prevalence and disease burden of vitamin D deficiency: A roadmap for action in low- and middle-income countries. Ann. N. Y. Acad. Sci..

[13] Bresson J.L., Burlingame B., Dean T., Fairweather-Tait S., Heinonen M., Hirsch-Ernst K.I., Mangelsdorf I., McArdle H., Naska A., Neuhäuser-Berthold M. (2016). Dietary Reference Values for Vitamin D. EFSA J..

[14] Munns C.F., Shaw N., Kiely M., Specker B.L., Thacher T.D., Ozono K., Michigami T., Tiosano D., Mughal M.Z., Mäkitie O. (2016). Global Consensus Recommendations on Prevention and Management of Nutritional Rickets. J. Clin. Endocrinol. Metab..

[15] Allen L., De Benoist B., Dary O., Hurrell R. (2006). Guidelines on Food Fortification with Micronutrients.

[16] Cashman K.D. (2020). Vitamin D Deficiency: Defining, Prevalence, Causes, and Strategies of Addressing. Calcif. Tissue Int..

[17] Sarafin K., Durazo-Arvizu R., Tian L., Phinney K.W., Tai S., Camara J.E., Merkel J., Green E., Sempos C.T., Brooks S.P. (2015). Standardizing 25-hydroxyvitamin D values from the Canadian Health Measures Survey. Am. J. Clin. Nutr..

[18] Schleicher R.L., Sternberg M.R., Looker A.C., Yetley E.A., Lacher D.A., Sempos C.T., Taylor C.L., Durazo-Arvizu R.A., Maw K.L., Chaudhary-Webb M. (2016). National Estimates of Serum Total 25-Hydroxyvitamin D and Metabolite Concentrations Measured by Liquid Chromatography–Tandem Mass Spectrometry in the US Population during 2007–2010. J. Nutr..

[19] Lippi G., Nouvenne A., Ticinesi A., Bonelli P., Salvagno G.L., Cervellin G., Guidi G.C. (2015). The burden of vitamin D deficiency in a mediterranean country without a policy of food fortification. Acta Bio-Med..

[20] Amrein K., Scherkl M., Hoffmann M., Neuwersch-Sommeregger S., Köstenberger M., Berisha A.T., Martucci G., Pilz S., Malle O. (2020). Vitamin D deficiency 2.0: An update on the current status worldwide. Eur. J. Clin. Nutr..

[21] Tsiaras W.G., Weinstock M.A. (2011). Factors Influencing Vitamin D Status. Acta Derm. Venereol..

[22] Kift R., Berry J., Vail A., Durkin M., Rhodes L., Webb A. (2013). Lifestyle factors including less cutaneous sun exposure contribute to starkly lower vitamin D levels in U.K. South Asians compared with the white population. Br. J. Dermatol..

[23] Marinov D.B., Dimitrova T.T. (2021). Vitamin D Status and Dietary Habits Of Children with Adolescent Idiopathic Scoliosis in Varna. J. IMAB—Annu. Proceeding Sci. Pap..

[24] Ganji V., Shi Z., Al-Abdi T., Al Hejap D., Attia Y., Koukach D., Elkassas H. (2023). Association between food intake patterns and serum vitamin D concentrations in US adults. Br. J. Nutr..

[25] Palacios C., Gonzalez L. (2014). Is vitamin D deficiency a major global public health problem?. J. Steroid Biochem. Mol. Biol..

[26] Weaver C.M., Dwyer J., Fulgoni V.L., King J.C., Leveille G.A., MacDonald R.S., Ordovas J., Schnakenberg D. (2014). Processed foods: Contributions to nutrition. Am. J. Clin. Nutr..

[27] Lips P., Cashman K.D., Lamberg-Allardt C., Bischoff-Ferrari H.A., Obermayer-Pietsch B., Bianchi M.L., Stepan J., El-Hajj Fuleihan G., Bouillon R. (2019). Current vitamin D status in European and Middle East countries and strategies to prevent vitamin D deficiency: A position statement of the European Calcified Tissue Society. Eur. J. Endocrinol..

[28] Niedermaier T., Gredner T., Kuznia S., Schöttker B., Mons U., Lakerveld J., Ahrens W., Brenner H., On behalf of the PEN-Consortium (2022). Vitamin D food fortification in European countries: The underused potential to prevent cancer deaths. Eur. J. Epidemiol..

[29] Pollock M., Fernandes R.M., Becker L.A., Pieper D., Hartling L., Higgins J., Thomas J., Chandler J., Cumpston M., Li T., Page M., Welch V. (2022). Overviews of Reviews. Cochrane Handbook for Systematic Reviews of Interventions.

[30] Gates M., Gates A., Pieper D., Fernandes R.M., Tricco A.C., Moher D., Brennan S.E., Li T., Pollock M., Lunny C. (2022). Reporting guideline for overviews of reviews of healthcare interventions: Development of the PRIOR statement. BMJ.

[31] Moher D., Liberati A., Tetzlaff J., Altman D.G. (2009). Preferred reporting items for systematic reviews and meta-analyses: The PRISMA statement. PLoS Med..

[32] Avau B., Van Remoortel H., De Buck E. (2021). Translation and validation of PubMed and Embase search filters for identification of systematic reviews, intervention studies, and observational studies in the field of first aid. J. Med. Libr. Assoc..

[33] Montori V.M., Wilczynski N.L., Morgan D., Haynes R.B. (2005). Optimal search strategies for retrieving systematic reviews from Medline: Analytical survey. BMJ.

[34] Shea B.J., Hamel C., Wells G.A., Bouter L., Kristjansson E., Grimshaw J., Henry D., Boers M. (2009). AMSTAR is a reliable and valid measurement tool to assess the methodological quality of systematic reviews. J. Clin. Epidemiol..

[35] Cranney A., Horsley T., O’Donnell S., Weiler H., Puil L., Ooi D., Atkinson S., Ward L., Moher D., Hanley D. (2007). Effectiveness and Safety of Vitamin D in Relation to Bone Health. Evid. Rep. Technol. Assess..

[36] Whiting S.J., Bonjour J.-P., Payen F.D., Rousseau B. (2015). Moderate Amounts of Vitamin D3 in Supplements are Effective in Raising Serum 25-Hydroxyvitamin D from Low Baseline Levels in Adults: A Systematic Review. Nutrients.

[37] Tangestani H., Djafarian K., Emamat H., Arabzadegan N., Shab-Bidar S. (2019). Efficacy of vitamin D fortified foods on bone mineral density and serum bone biomarkers: A systematic review and meta-analysis of interventional studies. Crit. Rev. Food Sci. Nutr..

[38] Souza S.V., Borges N., Vieira E.F. (2022). Vitamin d-fortified bread: Systematic review of fortification approaches and clinical studies. Food Chem..

[39] Soto-Méndez M.J., Rangel-Huerta O.D., Ruiz-López M.D., de Victoria E., Anguita-Ruiz A., Gil A. (2019). Role of Functional For-tified Dairy Products in Cardiometabolic Health: A Systematic Review and Meta-Analyses of Randomized Clinical Trials. Adv. Nutr..

[40] OMahony L., Stepien M., Gibney M.J., Nugent A.P., Brennan L. (2011). The Potential Role of Vitamin D Enhanced Foods in Improving Vitamin D Status. Nutrients.

[41] O’Donnell S., Cranney A., Horsley T., Weiler H.A., Atkinson S.A., Hanley D.A., Ooi D.S., Ward L., Barrowman N., Fang M. (2008). Efficacy of food fortification on serum 25-hydroxyvitamin D concentrations: Systematic review. Am. J. Clin. Nutr..

[42] Nikooyeh B., Neyestani T.R. (2021). The effects of vitamin D-fortified foods on circulating 25(OH)D concentrations in adults: A systematic review and meta-analysis. Br. J. Nutr..

[43] Nikooyeh B., Neyestani T. (2018). Efficacy of Food Fortification with Vitamin D in Iranian Adults: A Systematic Review and Meta-Analysis. Nutr. Food Sci. Res..

[44] Nikooyeh B., Ghodsi D., Neyestani T.R. (2021). How Much Does Serum 25(OH)D Improve by Vitamin D Supplement and Fortified Food in Children? A Systematic Review and Meta-Analysis. J. Pediatr. Gastroenterol. Nutr..

[45] Niedermaier T., Gredner T., Kuznia S., Schöttker B., Mons U., Brenner H. (2021). Potential of Vitamin D Food Fortification in Prevention of Cancer Deaths—A Modeling Study. Nutrients.

[46] Lam I.T., Keller H.H., Pfisterer K., Duizer L., Stark K., Duncan A.M. (2016). Micronutrient Food Fortification for Residential Care: A Scoping Review of Current Interventions. J. Am. Med. Dir. Assoc..

[47] Gasparri C., Perna S., Spadaccini D., Alalwan T., Girometta C., Infantino V., Rondanelli M. (2019). Is vitamin D-fortified yogurt a value-added strategy for improving human health? A systematic review and meta-analysis of randomized trials. J. Dairy Sci..

[48] Fonseca Santos R.K., Santos C.B., Reis A.R., Brandão-Lima P.N., de Carvalho G.B., Martini L.A., Pires L.V. (2021). Role of Food Fortification with Vitamin D and Calcium in the Bone Remodeling Process in Postmenopausal Women: A Systematic Review of Randomized Controlled Trials. Nutr. Rev..

[49] Emadzadeh M., Sahebi R., Khedmatgozar H., Sadeghi R., Farjami M., Sharifan P., Ravanshad Y., Ferns G.A., Ghayour-Mobarhan M. (2020). A systematic review and meta-analysis of the effect of Vitamin D-fortified food on glycemic indices. BioFactors.

[50] Emadzadeh M., Rashidmayvan M., Sahebi R., Sadeghi R., Ferns G.A., Ghayour-Mobarhan M. (2020). The effect of vitamin D fortified products on anthropometric indices: A systematic review and meta-analysis. Complement. Ther. Clin. Pract..

[51] Emadzadeh M., Mehdizadeh A., Sharifan P., Khoshakhlagh M., Sahebi R., Sadeghi R., Ferns G.A., Ghayour-Mobarhan M. (2022). The Effects of Vitamin D Fortified Products on Bone Biomarkers: A Systematic Review and Meta-Analysis. Iran. J. Public Health.

[52] Dunlop E., Kiely M.E., James A.P., Singh T., Pham N.M., Black L.J. (2021). Vitamin D Food Fortification and Biofortification In-creases Serum 25-Hydroxyvitamin D Concentrations in Adults and Children: An Updated and Extended Systematic Review and Meta-Analysis of Randomized Controlled Trials. J. Nutr..

[53] Das J.K., Salam R.A., Kumar R., Bhutta Z.A. (2013). Micronutrient fortification of food and its impact on woman and child health: A systematic review. Syst. Rev..

[54] Cashman K.D., Kiely M.E., Andersen R., Grønborg I.M., Madsen K.H., Nissen J., Tetens I., Tripkovic L., Lanham-New S.A., Toxqui L. (2020). Individual participant data (IPD)-level meta-analysis of randomised controlled trials with vitamin D-fortified foods to estimate Dietary Reference Values for vitamin D. Eur. J. Nutr..

[55] Cranney A., Weiler H.A., O’Donnell S., Puil L. (2008). Summary of evidence-based review on vitamin D efficacy and safety in relation to bone health. Am. J. Clin. Nutr..

[56] Brooker P.G., Rebuli M.A., Williams G., Muhlhausler B.S. (2022). Effect of Fortified Formula on Growth and Nutritional Status in Young Children: A Systematic Review and Meta-Analysis. Nutrients.

[57] Brett N.R., Gharibeh N., Weiler H.A. (2018). Effect of Vitamin D Supplementation, Food Fortification, or Bolus Injection on Vitamin D Status in Children Aged 2–18 Years: A Meta-Analysis. Adv. Nutr..

[58] Brandão-Lima P.N., Santos B., da C., Aguilera C.M., Freire A.R.S., Martins-Filho P.R.S., Pires L.V. (2019). Vitamin D Food Fortifi-cation and Nutritional Status in Children: A Systematic Review of Randomized Controlled Trials. Nutrients.

[59] Black L.J., Seamans K.M., Cashman K.D., Kiely M. (2012). An Updated Systematic Review and Meta-Analysis of the Efficacy of Vitamin D Food Fortification. J. Nutr..

[60] Al Khalifah R., Alsheikh R., Alnasser Y., Alsheikh R., Alhelali N., Naji A., Al Backer N. (2020). The impact of vitamin D food fortification and health outcomes in children: A systematic review and meta-regression. Syst. Rev..

[61] Aguiar M., Andronis L., Pallan M., Högler W., Frew E. (2017). Preventing vitamin D deficiency (VDD): A systematic review of economic evaluations. Eur. J. Public Health.

[62] Akkermans M.D., Eussen S.R., van der Horst-Graat J.M., van Elburg R.M., van Goudoever J.B., Brus F. (2017). A Micronutrient-Fortified Young-Child Formula Improves the Iron and Vitamin D Status of Healthy Young European Children: A Randomized, Double-Blind Controlled Trial. Am. J. Clin. Nutr..

[63] Benjeddou K., Qandoussi L., Mekkaoui B., Rabi B., El Hamdouchi A., Raji F., Saeid N., Belghiti H., Elkari K., Aguenaou H. (2019). Effect of multiple micronutrient fortified milk consumption on vitamin D status among school-aged children in rural region of Morocco. Appl. Physiol. Nutr. Metab..

[64] Brett N.R., Lavery P., Agellon S., Vanstone C.A., Maguire J.L., Rauch F., Weiler H.A. (2016). Dietary vitamin D dose-response in healthy children 2 to 8 y of age: A 12-wk randomized controlled trial using fortified foods. Am. J. Clin. Nutr..

[65] Brett N.R., Parks C.A., Lavery P., Agellon S., Vanstone C.A., Kaufmann M., Jones G., Maguire J.L., Rauch F., Weiler H.A. (2018). Vitamin D status and functional health outcomes in children aged 2–8 y: A 6-mo vitamin D randomized controlled trial. Am. J. Clin. Nutr..

[66] Economos C.D., Moore C.E., Hyatt R.R., Kuder J., Chen T., Meydani S.N., Meydani M., Klein E., Biancuzzo R.M., Holick M.F. (2014). Multinutrient-Fortified Juices Improve Vitamin D and Vitamin E Status in Children: A Randomized Controlled Trial. J. Acad. Nutr. Diet..

[67] Graham D., Kira G., Conaglen J., McLennan S., Rush E. (2008). Vitamin D status of Year 3 children and supplementation through schools with fortified milk. Public Health Nutr..

[68] Houghton L.A., Gray A.R., Szymlek-Gay E.A., Heath A.-L.M., Ferguson E.L. (2011). Vitamin D-Fortified Milk Achieves the Targeted Serum 25-Hydroxyvitamin D Concentration without Affecting That of Parathyroid Hormone in New Zealand Toddlers. J. Nutr..

[69] Hower J., Knoll A., Ritzenthaler K.L., Steiner C., Berwind R. (2013). Vitamin D fortification of growing up milk prevents decrease of serum 25-hydroxyvitamin D concentrations during winter: A clinical intervention study in Germany. Eur. J. Pediatr..

[70] Khadgawat R., Marwaha R.K., Garg M.K., Ramot R., Oberoi A.K., Sreenivas V., Gahlot M., Mehan N., Mathur P., Gupta N. (2013). Impact of vitamin D fortified milk supplementation on vitamin D status of healthy school children aged 10–14 years. Osteoporos. Int..

[71] Kuriyan R., Thankachan P., Selvam S., Pauline M., Srinivasan K., Kamath-Jha S., Vinoy S., Misra S., Finnegan Y., Kurpad A. (2016). V The Effects of Regular Consumption of a Multiple Micronutrient Fortified Milk Beverage on the Micronutrient Status of School Children and on Their Mental and Physical Performance. Clin. Nutr..

[72] Madsen K.H., Rasmussen L.B., Andersen R., Molgaard C., Jakobsen J., Bjerrum P.J., Andersen E.W., Mejborn H., Tetens I. (2013). Randomized Controlled Trial of the Effects of Vitamin D–Fortified Milk and Bread on Serum 25-Hydroxyvitamin D Concentrations in Families in Denmark during Winter: The VitmaD Study. Am. J. Clin. Nutr..

[73] Neyestani T.R., Hajifaraji M., Omidvar N., Nikooyeh B., Eshraghian M.R., Shariatzadeh N., Kalayi A., Khalaji N., Zahedirad M., Abtahi M. (2014). Calcium-vitamin D-fortified milk is as effective on circulating bone biomarkers as fortified juice and supplement but has less acceptance: A randomised controlled school-based trial. J. Hum. Nutr. Diet..

[74] Öhlund I., Lind T., Hernell O., Silfverdal S.-A., Karlsland Åkeson P. (2017). Increased vitamin D intake differentiated according to skin color is needed to meet requirements in young Swedish children during winter: A double-blind randomized clinical trial. Am. J. Clin. Nutr..

[75] Powers H.J., Stephens M., Russell J., Hill M.H. (2015). Fortified breakfast cereal consumed daily for 12 wk leads to a significant improvement in micronutrient intake and micronutrient status in adolescent girls: A randomised controlled trial. Nutr. J..

[76] Rich-Edwards J.W., Ganmaa D., Kleinman K., Sumberzul N., Holick M.F., Lkhagvasuren T., Dulguun B., Burke A., Frazier A.L. (2011). Randomized trial of fortified milk and supplements to raise 25-hydroxyvitamin D concentrations in schoolchildren in Mongolia. Am. J. Clin. Nutr..

[77] Wang X., Hui Z., Dai X., Terry P., Zhang Y., Ma M., Wang M., Deng F., Gu W., Lei S. (2017). Micronutrient Fortified Milk and Academic Performance among Chinese Middle School Students: A Cluster Randomized Controlled Trial. Nutrients.

[78] Moreira-lucas T.S., Duncan A.M., Rabasa-lhoret R., Vieth R., Gibbs A., Badawi A., Wolever T.M. (2016). Effect of Vitamin D For-tified Cheese on Oral Glucose Tolerance in Individuals Exhibiting Marginal Vitamin D Status and an Increased Risk for De-veloping Type 2 Diabetes: A Double-Blind, Randomized Placebo-Controlled Clinical Trial. FASEB J..

[79] Adolphi B., Scholz-Ahrens K.E., de Vrese M., Açil Y., Laue C., Schrezenmeir J. (2008). Short-Term Effect of Bedtime Consumption of Fermented Milk Supplemented with Calcium, Inulin-Type Fructans and Caseinphosphopeptides on Bone Metabolism in Healthy, Postmenopausal Women. Eur. J. Nutr..

[80] Al-Daghri N.M., Amer O.E., Khattak M.N., Sabico S., Ansari M.G.A., Al-Saleh Y., Aljohani N., Alfawaz H., Alokail M.S. (2019). Effects of different vitamin D supplementation strategies in reversing metabolic syndrome and its component risk factors in adolescents. J. Steroid Biochem. Mol. Biol..

[81] Sivakumar B., Nair K.M., Sreeramulu D., Suryanarayana P., Ravinder P., Shatrugna V., Kumar P.A., Raghunath M., Vikas Rao V., Balakrishna N. (2006). Effect of Micronutrient Supplement on Health and Nutritional Status of Schoolchildren: Biochemical Status. Nutrition.

[82] Biancuzzo R.M., Young A., Bibuld D., Cai M.H., Winter M.R., Klein E.K., Ameri A., Reitz R., Salameh W., Chen T. (2010). Fortification of orange juice with vitamin D_2_ or vitamin D_3_ is as effective as an oral supplement in maintaining vitamin D status in adults. Am. J. Clin. Nutr..

[83] Bonjour J.P., Carrie A.L., Ferrari S., Clavien H., Slosman D., Theintz G., Rizzoli R. (1997). Calcium-enriched foods and bone mass growth in prepubertal girls: A randomized, double-blind, placebo-controlled trial. J. Clin. Investig..

[84] Bonjour J.-P., Benoit V., Pourchaire O., Ferry M., Rousseau B., Souberbielle J.-C. (2009). Inhibition of markers of bone resorption by consumption of vitamin D and calcium-fortified soft plain cheese by institutionalised elderly women. Br. J. Nutr..

[85] Bonjour J.-P., Benoit V., Pourchaire O., Rousseau B., Souberbielle J.-C. (2011). Nutritional approach for inhibiting bone resorption in institutionalized elderly women with vitamin D insufficiency and high prevalence of fracture. J. Nutr. Health Aging.

[86] Bonjour J.-P., Benoit V., Rousseau B., Souberbielle J.-C. (2012). Consumption of Vitamin D-and Calcium-Fortified Soft White Cheese Lowers the Biochemical Marker of Bone Resorption TRAP 5b in Postmenopausal Women at Moderate Risk of Osteoporosis Fracture. J. Nutr..

[87] Bonjour J.P., Benoit V., Payen F., Kraenzlin M. (2013). Consumption of Yogurts Fortified in Vitamin D and Calcium Reduces Serum Parathyroid Hormone and Markers of Bone Resorption: A Double-Blind Randomized Controlled Trial in Institutionalized El-derly Women. J. Clin. Endocrinol. Metab..

[88] Bonjour J.-P., Benoit V., Atkin S., Walrand S. (2015). Fortification of yogurts with vitamin D and calcium enhances the inhibition of serum parathyroid hormone and bone resorption markers: A double blind randomized controlled trial in women over 60 living in a community dwelling home. J. Nutr. Health Aging.

[89] Bonjour J.P., Dontot-Payen F., Rouy E., Walrand S., Rousseau B. (2017). Evolution of Serum 25OHD in Response to Vitamin D3–Fortified Yogurts Consumed by Healthy Menopausal Women: A 6-Month Randomized Controlled Trial Assessing the Interactions between Doses, Baseline Vitamin D Status, and Seasonality. J. Am. Coll. Nutr..

[90] Chee W.S.S., Suriah A.R., Chan S.P., Zaitun Y., Chan Y.M. (2003). The effect of milk supplementation on bone mineral density in postmenopausal Chinese women in Malaysia. Osteoporos. Int..

[91] Costan A.R., Vulpoi C., Mocanu V. (2014). Vitamin D Fortified Bread Improves Pain and Physical Function Domains of Quality of Life in Nursing Home Residents. J. Med. Food.

[92] Daly R.M., Bass S., Nowson C. (2006). Long-term effects of calcium–vitamin-D3-fortified milk on bone geometry and strength in older men. Bone.

[93] Daly R.M., Brown M., Bass S., Kukuljan S., Nowson C. (2005). Calcium- and Vitamin D3-Fortified Milk Reduces Bone Loss at Clinically Relevant Skeletal Sites in Older Men: A 2-Year Randomized Controlled Trial. J. Bone Miner. Res..

[94] Daly R.M., Petrass N., Bass S., Nowson C.A. (2008). The skeletal benefits of calcium- and vitamin D3–fortified milk are sustained in older men after withdrawal of supplementation: An 18-mo follow-up study. Am. J. Clin. Nutr..

[95] Daly R.M., Nowson C.A. (2009). Long-Term Effect of Calcium-Vitamin D3 Fortified Milk on Blood Pressure and Serum Lipid Concentrations in Healthy Older Men. Eur. J. Clin. Nutr..

[96] De Jong N., Paw M.J.C.A., de Groot L.C., de Graaf C., Kok F.J., van Staveren W.A. (1999). Functional Biochemical and Nutrient Indices in Frail Elderly People Are Partly Affected by Dietary Supplements but Not by Exercise. J. Nutr..

[97] Fisk C.M., Theobald H.E., Sanders T.A.B. (2012). Fortified Malted Milk Drinks Containing Low-Dose Ergocalciferol and Cholecalciferol Do Not Differ in Their Capacity to Raise Serum 25-Hydroxyvitamin D Concentrations in Healthy Men and Women Not Exposed to UV-B. J. Nutr..

[98] Gaffney-Stomberg E., Lutz L.J., Rood J.C., Cable S.J., Pasiakos S.M., Young A.J., McClung J.P. (2014). Calcium and vitamin D supplementation maintains parathyroid hormone and improves bone density during initial military training: A randomized, double-blind, placebo controlled trial. Bone.

[99] Gaffney-Stomberg E., Nakayama A.T., Guerriere K.I., Lutz L.J., Walker L.A., Staab J.S., Scott J.M., Gasier H.G., McClung J.P. (2019). Calcium and vitamin D supplementation and bone health in Marine recruits: Effect of season. Bone.

[100] Ganmaa D., Stuart J.J., Sumberzul N., Ninjin B., Giovannucci E., Kleinman K., Holick M.F., Willett W.C., Frazier L.A., Rich-Edwards J.W. (2017). Vitamin D supplementation and growth in urban Mongol school children: Results from two randomized clinical trials. PLoS ONE.

[101] Grønborg I.M., Tetens I., Andersen E.W., Kristensen M., Larsen R.E.K., Tran T.L.L., Andersen R. (2019). Effect of Vitamin D Fortified Foods on Bone Markers and Muscle Strength in Women of Pakistani and Danish Origin Living in Denmark: A Randomised Controlled Trial. Nutr. J..

[102] Grønborg I.M., Tetens I., Christensen T., Andersen E.W., Jakobsen J., Kiely M., Cashman K.D., Andersen R. (2019). Vitamin D-fortified foods improve wintertime vitamin D status in women of Danish and Pakistani origin living in Denmark: A randomized controlled trial. Eur. J. Nutr..

[103] Hayes A., Duffy S., O’grady M., Jakobsen J., Galvin K., Teahan-Dillon J., Kerry J., Kelly A., O’doherty J., Higgins S. (2016). Vitamin D–enhanced eggs are protective of wintertime serum 25-hydroxyvitamin D in a randomized controlled trial of adults. Am. J. Clin. Nutr..

[104] Hennigar S.R., Gaffney-Stomberg E., Lutz L.J., Cable S.J., Pasiakos S.M., Young A.J., McClung J.P. (2015). Consumption of a Calcium and Vitamin D-Fortified Food Product Does Not Affect Iron Status during Initial Military Training: A Randomised, Double-Blind, Placebo-Controlled Trial. Br. J. Nutr..

[105] Heravifard S., Neyestani T.R., Nikooyeh B., Alavi-Majd H., Houshiarrad A., Kalayi A., Shariatzadeh N., Zahedirad M., Tayebinejad N., Salekzamani S. (2013). Regular Consumption of Both Vitamin D– and Calcium- and Vitamin D–Fortified Yogurt Drink Is Equally Accompanied by Lowered Blood Lipoprotein (a) and Elevated Apoprotein A1 in Subjects with Type 2 Diabetes: A Randomized Clinical Trial. J. Am. Coll. Nutr..

[106] Hettiarachchi M., Lekamwasam S., Liyanage C. (2010). Long-term cereal-based nutritional supplementation improved the total spine bone mineral density amongst Sri Lankan preschool children: A randomized controlled study. J. Pediatr. Endocrinol. Metab..

[107] Ho S.C., Guldan G.S., Woo J., Yu R., Tse M.M., Sham A., Cheng J. (2005). A Prospective Study of the Effects of 1-Year Calcium-Fortified Soy Milk Supplementation on Dietary Calcium Intake and Bone Health in Chinese Adolescent Girls Aged 14 to 16. Osteoporos. Int..

[108] Itkonen S.T., Skaffari E., Saaristo P., Saarnio E.M., Erkkola M., Jakobsen J., Cashman K.D., Lamberg-Allardt C. (2016). Effects of Vitamin D2-Fortified Breadv. Supplementation with Vitamin D2 or D3 on Serum 25-Hydroxyvitamin D Metabolites: An 8-Week Randomised-Controlled Trial in Young Adult Finnish Women. Br. J. Nutr..

[109] Jääskeläinen T., Itkonen S.T., Lundqvist A., Erkkola M., Koskela T., Lakkala K., Dowling K.G., Hull G.L., Kröger H., Karppinen J. (2017). The positive impact of general vitamin D food fortification policy on vitamin D status in a representative adult Finnish population: Evidence from an 11-y follow-up based on standardized 25-hydroxyvitamin D data. Am. J. Clin. Nutr..

[110] Jafari T., Faghihimani E., Feizi A., Iraj B., Javanmard S.H., Esmaillzadeh A., Fallah A.A., Askari G. (2016). Effects of vitamin D-fortified low fat yogurt on glycemic status, anthropometric indexes, inflammation, and bone turnover in diabetic postmenopausal women: A randomised controlled clinical trial. Clin. Nutr..

[111] Jakobsen J., Knuthsen P. (2014). Stability of vitamin D in foodstuffs during cooking. Food Chem..

[112] Johnson J., Mistry V., Vukovich M.D., Hogie-Lorenzen T., Hollis B., Specker B. (2005). Bioavailability of Vitamin D from Fortified Process Cheese and Effects on Vitamin D Status in the Elderly. J. Dairy Sci..

[113] Kanellakis S., Moschonis G., Tenta R., Schaafsma A., van den Heuvel E.G.H.M., Papaioannou N., Lyritis G., Manios Y. (2012). Changes in Parameters of Bone Metabolism in Postmenopausal Women Following a 12-Month Intervention Period Using Dairy Products Enriched with Calcium, Vitamin D, and Phylloquinone (Vitamin K_1_) or Menaquinone-7 (Vitamin K_2_): The Postmenopausal Health Study II. Calcif. Tissue Int..

[114] Keane E.M., Rochfort A., Cox J., McGovern D., Coakley D., Walsh B. (1992). Vitamin-D-Fortified Liquid Milk—A Highly Effective Method of Vitamin D Administration for House-Bound and Institutionalised Elderly. Gerontology.

[115] Keane E.M., Healy M., O’Moore R., Coakley D., Walsh J.B. (1998). Vitamin D-Fortified Liquid Milk: Benefits for the Elderly Com-munity-Based Population. Calcif. Tissue Int..

[116] Kruger M.C., Booth C.L., Coad J., Schollum L.M., Kuhn-Sherlock B., Shearer M.J. (2006). Effect of Calcium Fortified Milk Supplementation with or without Vitamin K on Biochemical Markers of Bone Turnover in Premenopausal Women. Nutrition.

[117] Kruger M.C., Schollum L.M., Kuhn-Sherlock B., Hestiantoro A., Wijanto P., Li-Yu J., Agdeppa I., Todd J.M., Eastell R. (2010). The effect of a fortified milk drink on vitamin D status and bone turnover in post-menopausal women from South East Asia. Bone.

[118] Kruger M.C., Chan Y.M., Lau L.T., Lau C.C., Chin Y.S., Kuhn-Sherlock B., Todd J.M., Schollum L.M. (2017). Calcium and vitamin D fortified milk reduces bone turnover and improves bone density in postmenopausal women over 1 year. Eur. J. Nutr..

[119] Kruger M.C., Ha P.C., Todd J.M., Kuhn-Sherlock B., Schollum L.M., Ma J., Qin G., Lau E. (2012). High-Calcium, Vitamin D Fortified Milk Is Effective in Improving Bone Turnover Markers and Vitamin D Status in Healthy Postmenopausal Chinese Women. Eur. J. Clin. Nutr..

[120] Kruger M.C., Chan Y.M., Lau C., Lau L.T., Chin Y.S., Kuhn-Sherlock B., Schollum L.M., Todd J.M. (2019). Fortified Milk Supplementation Improves Vitamin D Status, Grip Strength, and Maintains Bone Density in Chinese Premenopausal Women Living in Malaysia. BioResearch Open Access.

[121] Kukuljan S., Nowson C.A., Sanders K., Daly R.M. (2009). Effects of resistance exercise and fortified milk on skeletal muscle mass, muscle size, and functional performance in middle-aged and older men: An 18-mo randomized controlled trial. J. Appl. Physiol..

[122] Kukuljan S., Nowson C.A., Bass S.L., Sanders K., Nicholson G.C., Seibel M.J., Salmon J., Daly R.M. (2008). Effects of a Multi-Component Exercise Program and Calcium–Vitamin-D3-Fortified Milk on Bone Mineral Density in Older Men: A Randomised Controlled Trial. Osteoporos. Int..

[123] Kukuljan S., Nowson C.A., Sanders K.M., Nicholson G.C., Seibel M.J., Salmon J., Daly R.M. (2011). Independent and Combined Effects of Calcium-Vitamin D3 and Exercise on Bone Structure and Strength in Older Men: An 18-Month Factorial Design Randomized Controlled Trial. J. Clin. Endocrinol. Metab..

[124] Lau E.M.C., Woo J., Lam V., Hong A. (2001). Milk Supplementation of the Diet of Postmenopausal Chinese Women on a Low Calcium Intake Retards Bone Loss. J. Bone Miner. Res..

[125] Lehtonen-Veromaa M., Möttönen T., Leino A., Heinonen O.J., Rautava E., Viikari J. (2008). Prospective study on food fortification with vitamin D among adolescent females in Finland: Minor effects. Br. J. Nutr..

[126] Li Q., Xing B. (2016). Vitamin D3-Supplemented Yogurt Drink Improves Insulin Resistance and Lipid Profiles in Women with Gestational Diabetes Mellitus: A Randomized Double Blinded Clinical Trial. Ann. Nutr. Metab..

[127] Lovell A.L., Davies P.S.W., Hill R.J., Milne T., Matsuyama M., Jiang Y., Chen R.X., Wouldes T.A., Heath A.L.M., Grant C.C. (2018). Compared with Cow Milk, a Growing-Up Milk Increases Vitamin D and Iron Status in Healthy Children at 2 Years of Age: The Growing-Up Milk–Lite (GUMLi) Randomized Controlled Trial. J. Nutr..

[128] Lu J., Pan H., Hu X., Huang Z., Zhang Q. (2019). Effects of milk powder intervention on bone mineral density and indicators related to bone metabolism in Chinese adolescents. Osteoporos. Int..

[129] Manios Y., Moschonis G., Trovas G., Lyritis G.P. (2007). Changes in biochemical indexes of bone metabolism and bone mineral density after a 12-mo dietary intervention program: The Postmenopausal Health Study. Am. J. Clin. Nutr..

[130] Manios Y., Moschonis G., Panagiotakos D.B., Farajian P., Trovas G., Lyritis G.P. (2009). Changes in biochemical indices of bone metabolism in post-menopausal women following a dietary intervention with fortified dairy products. J. Hum. Nutr. Diet..

[131] Manios Y., Moschonis G., Lyritis G.P. (2011). Seasonal variations of vitamin D status in Greek postmenopausal women receiving enriched dairy products for 30 months: The Postmenopausal Health Study. Eur. J. Clin. Nutr..

[132] Manios Y., Moschonis G., Mavrogianni C., van den Heuvel E., Singh-Povel C.M., Kiely M., Cashman K.D. (2016). Reduced-Fat Gouda-Type Cheese Enriched with Vitamin D3 Effectively Prevents Vitamin D Deficiency during Winter Months in Post-menopausal Women in Greece. Eur. J. Nutr..

[133] Mocanu V., Stitt P.A., Costan A.R., Voroniuc O., Zbranca E., Luca V., Vieth R. (2009). Long-Term Effects of Giving Nursing Home Residents Bread Fortified with 125 μg (5000 IU) Vitamin D3 per Daily Serving. Am. J. Clin. Nutr..

[134] Mocanu V., Vieth R. (2013). Three-Year Follow-up of Serum 25-Hydroxyvitamin D, Parathyroid Hormone, and Bone Mineral Density in Nursing Home Residents Who Had Received 12 Months of Daily Bread Fortification with 125 μg of Vitamin D3. Nutr. J..

[135] Mohammadi-Sartang M., Bellissimo N., de Zepetnek J.O.T., Brett N.R., Mazloomi S.M., Fararouie M., Bedeltavana A., Famouri M., Mazloom Z. (2018). The effect of daily fortified yogurt consumption on weight loss in adults with metabolic syndrome: A 10-week randomized controlled trial. Nutr. Metab. Cardiovasc. Dis..

[136] Moreira-Lucas T.S., Duncan A.M., Rabasa-Lhoret R., Vieth R., Gibbs A.L., Badawi A., Wolever T.M. (2016). Effect of vitamin D supplementation on oral glucose tolerance in individuals with low vitamin D status and increased risk for developing type 2 diabetes (EVIDENCE): A double-blind, randomized, placebo-controlled clinical trial. Diabetes Obes. Metab..

[137] Moschonis G., Manios Y. (2006). Skeletal site-dependent response of bone mineral density and quantitative ultrasound parameters following a 12-month dietary intervention using dairy products fortified with calcium and vitamin D: The Postmenopausal Health Study. Br. J. Nutr..

[138] Moschonis G., Katsaroli I., Lyritis G.P., Manios Y. (2010). The effects of a 30-month dietary intervention on bone mineral density: The Postmenopausal Health Study. Br. J. Nutr..

[139] Moschonis G., Kanellakis S., Papaioannou N., Schaafsma A., Manios Y. (2011). Possible Site-Specific Effect of an Intervention Combining Nutrition and Lifestyle Counselling with Consumption of Fortified Dairy Products on Bone Mass: The Postmenopausal Health Study II. J. Bone Miner. Metab..

[140] Mostafai R., Mohammadi R., Nachvak S.M., Rezaei M., Pasdar Y., Abdollahzad H., Rezvanmadani F., Moradi S., Morvaridzadeh M., Niazi P. (2018). Fortified Yogurt with Vitamin D as a Cost-Effective Food to Prevent Diabetes: A Randomized Double-Blind Clinical Trial. J. Funct. Foods.

[141] Natri A.-M., Salo P., Vikstedt T., Palssa A., Huttunen M., Kärkkäinen M.U., Salovaara H., Piironen V., Jakobsen J., Lamberg-Allardt C.J. (2006). Bread Fortified with Cholecalciferol Increases the Serum 25-HydroxyvitaminD Concentration in Women as Effectively as a Cholecalciferol Supplement. J. Nutr..

[142] Neyestani T.R., Nikooyeh B., Alavi-Majd H., Shariatzadeh N., Kalayi A., Tayebinejad N., Heravifard S., Salekzamani S., Zahedirad M. (2012). Improvement of Vitamin D Status via Daily Intake of Fortified Yogurt Drink Either with or without Extra Calcium Ameliorates Systemic Inflammatory Biomarkers, Including Adipokines, in the Subjects with Type 2 Diabetes. J. Clin. En-docrinol. Metab..

[143] Neyestani T.R., Nikooyeh B., Kalayi A., Zahedirad M., Shariatzadeh N. (2015). A Vitamin D-Calcium-Fortified Yogurt Drink De-creased Serum PTH but Did Not Affect Osteocalcin in Subjects with Type 2 Diabetes. Int. J. Vitam. Nutr. Res..

[144] Nikooyeh B., Neyestani T.R., Farvid M., Alavi-Majd H., Houshiarrad A., Kalayi A., Shariatzadeh N., Gharavi A., Heravifard S., Tayebinejad N. (2011). Daily consumption of vitamin D– or vitamin D + calcium–fortified yogurt drink improved glycemic control in patients with type 2 diabetes: A randomized clinical trial. Am. J. Clin. Nutr..

[145] Nikooyeh B., Neyestani T.R., Zahedirad M., Mohammadi M., Hosseini S.H., Abdollahi Z., Salehi F., Razaz J.M., Shariatzadeh N., Kalayi A. (2016). Vitamin D-Fortified Bread Is as Effective as Supplement in Improving Vitamin D Status: A Randomized Clinical Trial. J. Clin. Endocrinol. Metab..

[146] Nissen J., Vogel U., Ravn-Haren G., Andersen E.W., Nexø B.A., Andersen R., Mejborn H., Madsen K.H., Rasmussen L.B. (2014). Real-life use of vitamin D3-fortified bread and milk during a winter season: The effects of CYP2R1 and GC genes on 25-hydroxyvitamin D concentrations in Danish families, the VitmaD study. Genes Nutr..

[147] Nikooyeh B., Zargaraan A., Kalayi A., Shariatzadeh N., Zahedirad M., Jamali A., Khazraie M., Hollis B., Neyestani T.R. (2019). Vitamin D-fortified cooking oil is an effective way to improve vitamin D status: An institutional efficacy trial. Eur. J. Nutr..

[148] Palacios S., Castelo-Branco C., Cifuentes I., Von Helde S., Baró L., Tapia-Ruano C., Menéndez C., Rueda C. (2005). Changes in bone turnover markers after calcium-enriched milk supplementation in healthy postmenopausal women: A randomized, double-blind, prospective clinical trial. Menopause.

[149] Panunzio M.F., Pisano A., Telesforo P., Tomaiuolo P. (2003). Diet can increase 25-hydroxyvitamin-D3 plasma levels in the elderly: A dietary intervention trial. Nutr. Res..

[150] Recker R.R., Heaney R.P. (1985). The effect of milk supplements on calcium metabolism, bone metabolism and calcium balance. Am. J. Clin. Nutr..

[151] Rosenblum J.L., Castro V.M., Moore C.E., Kaplan L.M. (2012). Calcium and Vitamin D Supplementation Is Associated with De-creased Abdominal Visceral Adipose Tissue in Overweight and Obese Adults. Am. J. Clin. Nutr..

[152] Salehi S., Sadeghi F., Akhlaghi M., Hanifpour M.A., Roshanzamir M. (2018). Vitamin D3-fortified milk did not affect glycemic control, lipid profile, and anthropometric measures in patients with type 2 diabetes, a triple-blind randomized clinical trial. Eur. J. Clin. Nutr..

[153] Sandmann A., Amling M., Barvencik F., König H.H., Bleibler F. (2015). Economic Evaluation of Vitamin D and Calcium Food Fortification for Fracture Prevention in Germany. Public Health Nutr..

[154] Shab-Bidar S., Neyestani T.R., Djazayery A., Eshraghian M.-R., Houshiarrad A., Gharavi A., Kalayi A., Shariatzadeh N., Zahedirad M., Khalaji N. (2011). Regular consumption of vitamin D-fortified yogurt drink (Doogh) improved endothelial biomarkers in subjects with type 2 diabetes: A randomized double-blind clinical trial. BMC Med..

[155] Shab-Bidar S., Neyestani T.R., Djazayery A. (2011). Efficacy of Vitamin D3-Fortified-Yogurt Drink on Anthropometric, Metabolic, Inflammatory and Oxidative Stress Biomarkers According to Vitamin D Receptor Gene Polymorphisms in Type 2 Diabetic Patients: A Study Protocol for a Randomized Controlled Clinical Trial. BMC Endocr. Disord..

[156] Shab-Bidar S., Neyestani T.R., Djazayery A. (2015). Vitamin D receptor *Cdx-2*-dependent response of central obesity to vitamin D intake in the subjects with type 2 diabetes: A randomised clinical trial. Br. J. Nutr..

[157] Suzuki Y., Maruyama-Nagao A., Sakuraba K., Kawai S. (2014). Milk fortified with vitamin D could reduce the prevalence of vitamin D deficiency among Japanese female college students. Arch. Osteoporos..

[158] Tangpricha V., Koutkia P., Rieke S.M., Chen T.C., Perez A.A., Holick M.F. (2003). Fortification of orange juice with vitamin D: A novel approach for enhancing vitamin D nutritional health. Am. J. Clin. Nutr..

[159] Tenta R., Moschonis G., Koutsilieris M., Manios Y. (2010). Calcium and vitamin D supplementation through fortified dairy products counterbalances seasonal variations of bone metabolism indices: The Postmenopausal Health Study. Eur. J. Nutr..

[160] Toxqui L., Blanco-Rojo R., Wright I., Pérez-Granados A.M., Vaquero M.P. (2013). Changes in Blood Pressure and Lipid Levels in Young Women Consuming a Vitamin D-Fortified Skimmed Milk: A Randomised Controlled Trial. Nutrients.

[161] Toxqui L., Pérez-Granados A.M., Blanco-Rojo R., Wright I., Msc C.G.-V., Vaquero M.P. (2013). Effects of an Iron or Iron and Vitamin D–Fortified Flavored Skim Milk on Iron Metabolism: A Randomized Controlled Double-Blind Trial in Iron-Deficient Women. J. Am. Coll. Nutr..

[162] Toxqui L., Pérez-Granados A.M., Blanco-Rojo R., Wright I., de la Piedra C., Vaquero M.P. (2013). Low iron status as a factor of increased bone resorption and effects of an iron and vitamin D-fortified skimmed milk on bone remodelling in young Spanish women. Eur. J. Nutr..

[163] Trautvetter U., Neef N., Leiterer M., Kiehntopf M., Kratzsch J., Jahreis G. (2014). Effect of calcium phosphate and vitamin D3supplementation on bone remodelling and metabolism of calcium, phosphorus, magnesium and iron. Nutr. J..

[164] Tripkovic L., Wilson L.R., Hart K., Johnsen S., de Lusignan S., Smith C.P., Bucca G., Penson S., Chope G., Elliott R. (2017). Daily Supplementation with 15 μg Vitamin D2 Compared with Vitamin D3 to Increase Wintertime 25-Hydroxyvitamin D Status in Healthy South Asian and White European Women: A 12-Wk Randomized, Placebo-Controlled Food-Fortification Trial. Am. J. Clin. Nutr..

[165] Urbain P., Singler F., Ihorst G., Biesalski H.K., Bertz H. (2011). Bioavailability of Vitamin D2 from UV-B-Irradiated Button Mush-rooms in Healthy Adults Deficient in Serum 25-Hydroxyvitamin D: A Randomized Controlled Trial. Eur. J. Clin. Nutr..

[166] Wagner D., Sidhom G., Whiting S.J., Rousseau D., Vieth R. (2008). The Bioavailability of Vitamin D from Fortified Cheeses and Supplements Is Equivalent in Adults. J. Nutr..

[167] Woo J., Lau W., Xu L., Lam C.W.K., Zhao X., Yu W., Xing X., Lau E., Kuhn-Sherlock B., Pocock N. (2007). Milk Supple-mentation and Bone Health in Young Adult Chinese Women. J. Womens Health.

[168] Du X., Zhu K., Trube A., Zhang Q., Ma G., Hu X., Fraser D.R., Greenfield H. (2004). School-milk intervention trial enhances growth and bone mineral accretion in Chinese girls aged 10–12 years in Beijing. Br. J. Nutr..

[169] Zhu K., Du X., Cowell C.T., Greenfield H., Blades B., Dobbins T.A., Zhang Q., Fraser D.R. (2005). Effects of School Milk Intervention on Cortical Bone Accretion and Indicators Relevant to Bone Metabolism in Chinese Girls Aged 10–12 y in Beijing. Am. J. Clin. Nutr..

[170] Zhang Z.Q., Chen Y.M., Wang R.Q., Huang Z.W., Yang X.G., Su Y.X. (2015). The Effects of Different Levels of Calcium Supple-mentation on the Bone Mineral Status of Postpartum Lactating Chinese Women: A 12-Month Randomised, Double-Blinded, Controlled Trial. Br. J. Nutr..

[171] Zhu K., Greenfield H., Du X., Zhang Q., Ma G., Hu X., Cowell C.T., Fraser D. (2008). Effects of two years’ milk supplementation on size-corrected bone mineral density of Chinese girls. Asia Pac. J. Clin. Nutr..

[172] Sun Q., Yang B., Feng L., Chen Q., Liu Y. (2011). Prospective Study on Effect of Calcium and Vitamin D Fortified Drinks on Bone Development in Children. J. Jilin Univ. Med. Ed..

[173] McKenna M.J., Freaney R., Byrne P., McBrinn Y., Murray B., Kelly M., Donne B., O’Brien M. (1995). Safety and Efficacy of In-creasing Wintertime Vitamin D and Calcium Intake by Milk Fortification. QJM.

[174] Green T.J., Skeaff M., Rockell J.E. (2010). Milk Fortified with the Current Adequate Intake for Vitamin D (5μg) Increases Serum 25-Hydroxyvitamin D Compared to Control Milk but Is Not Sufficient to Prevent a Seasonal Decline in Young Women. Asia Pac. J. Clin. Nutr..

[175] Ganmaa D., Tserendolgor U., Frazier L., Nakamoto E., Jargalsaikhan N., Rich-Edwards J., Mph S. (2008). Effects of Vitamin D Fortified Milk on Vitamin D Status in Mongolian School Age Children. Asia Pac. J. Clin. Nutr..

[176] Gupta A. (2014). Fortification of Foods with Vitamin D in India. Nutrients.

[177] Pilz S., März W., Cashman K.D., Kiely M.E., Whiting S.J., Holick M.F., Grant W.B., Pludowski P., Hiligsmann M., Trummer C. (2018). Rationale and Plan for Vitamin D Food Fortification: A Review and Guidance Paper. Front. Endocrinol..

[178] Yang Z., Laillou A., Smith G., Schofield D., Moench-Pfanner R. (2013). A Review of Vitamin D Fortification: Implications for Nutrition Programming in Southeast Asia. Food Nutr. Bull..

[179] Grant W.B., Boucher B.J. (2019). A Review of the Potential Benefits of Increasing Vitamin D Status in Mongolian Adults through Food Fortification and Vitamin D Supplementation. Nutrients.

[180] Faggion C.M. (2015). Critical appraisal of AMSTAR: Challenges, limitations, and potential solutions from the perspective of an assessor. BMC Med. Res. Methodol..

[181] Cashman K.D., O’dea R. (2019). Exploration of strategic food vehicles for vitamin D fortification in low/lower-middle income countries. J. Steroid Biochem. Mol. Biol..

