# Supplementary material for: Fortification of Staple Foods for Household Use with Vitamin D: An Overview of Systematic Reviews
Source: Nutrients. 2023 Aug 26;15(17):3742. doi: 10.3390/nu15173742 (PMC10489979; doi:10.3390/nu15173742)
Supplement: Supplementary file 1 [file nutrients-15-03742-s001.zip › Supplementary_File_S2_Search_Strategy.pdf]

Ovid Medline

1. exp Vitamin D/
2. (vitamin D* or calciferol* or cholecalciferol* or colecalciferol* or ergocalciferol* or calciol*).tw.
3. or/1-2
4. Food, Fortified/
5. (fortif* or enrich* or enhanc*).tw.
6. or/4-5
7. Validated Sensitivity-and-precision balancing strategy by Montori et al. (REF: Montori VM, Wilczynski NL, Morgan D, Haynes RB; Hedges Team. Optimal search strategies for retrieving systematic reviews from Medline: analytical survey. BMJ. 2005 Jan 8;330(7482):68. doi: 10.1136/bmj.38336.804167.47.)

Meta-analysis.mp,pt. OR review.pt. OR search:.tw.

Embase

8. 'vitamin d'/exp
9. 'vitamin d*':ab,ti OR calciferol*:ab,ti OR cholecalciferol*:ab,ti OR colecalciferol*:ab,ti OR ergocalciferol*:ab,ti OR calciol*:ab,ti
10. #1 OR #2
11. 'fortified food'/exp
12. fortif*:ab,ti OR enrich*:ab,ti OR enhanc*:ab,ti
13. ((boost* OR increas* OR improv* OR add*) NEAR/3 ('vitamin d*' OR calciferol* OR cholecalciferol* OR colecalciferol* OR ergocalciferol* OR calciol*)):ab,ti
14. #4 OR #5 OR #6
15. #3 AND #7
1. Validated systematic review search filter by (REF: Avau B, Van Remoortel H, De Buck E. Translation and validation of PubMed and Embase search filters for identification of systematic reviews, intervention studies, and observational studies in the field of first aid. J Med Libr Assoc. 2021 Oct 1;109(4):599-608. doi: 10.5195/jmla.2021.1219.)

((‘meta analysis (topic)’/exp OR ‘meta analysis’/exp OR (meta NEXT/1 analy*):ab,ti OR metaanaly*:ab,ti OR ‘systematic review (topic)’/exp OR ‘systematic review’/exp OR (systematic NEXT/1 review*):ab,ti OR (systematic NEXT/1 overview*):ab,ti) OR (cancerlit:ab,ti OR cochrane:ab,ti OR embase:ab,ti OR psychlit:ab,ti OR psyclit:ab,ti OR psychinfo:ab,ti OR psycinfo:ab,ti OR cinahl:ab,ti OR cinhal:ab,ti OR ‘science citation index’:ab,ti OR bids:ab,ti) OR ((reference NEXT/1 list*):ab,ti OR bibliograph*:ab,ti OR hand-search*:ab,ti OR (manual NEXT/1 search*):ab,ti OR ‘relevant journals’:ab,ti) OR ((‘data extraction’:ab,ti OR ‘selection criteria’:ab,ti) AND review/it)) NOT (letter/it OR editorial/it OR (‘animal’/exp NOT (‘animal’/exp AND ‘human’/exp)))

Epistemonikos

(title:((vitamin D* OR calciferol* OR cholecalciferol* OR colecalciferol* OR ergocalciferol* OR calciol*)) OR abstract:((vitamin D* or calciferol* or cholecalciferol* or colecalciferol* or ergocalciferol* or calciol*))) AND (title:((fortif* or enrich* or enhanc*)) OR abstract:((fortif* or enrich* or enhanc*)))
Publication type: Systematic Review

Cochrane CENTRAL

1. MESH DESCRIPTOR Vitamin D EXPLODE ALL TREES
2. (vitamin D* OR calciferol* OR cholecalciferol* OR colecalciferol* OR ergocalciferol* OR calciol*):TI,AB,KY
3. #1 OR #2

4. MESH DESCRIPTOR Food, Fortified
5. (fortif* OR enrich* OR enhanc*):TI,AB,KY
6. ((boost* OR increas* OR improv* OR add*) ADJ3 (vitamin D* OR calciferol* OR cholecalciferol* OR colecalciferol* OR ergocalciferol* OR calciol*)):TI,AB,KY
7. #4 OR #5 OR #6
8. #3 AND #7
9. INREVIEW
