# Supplementary material for: Fortification of Staple Foods for Household Use with Vitamin D: An Overview of Systematic Reviews
Source: Nutrients. 2023 Aug 26;15(17):3742. doi: 10.3390/nu15173742 (PMC10489979; doi:10.3390/nu15173742)
Supplement: Supplementary file 1 [file nutrients-15-03742-s001.zip › Supplementary_File_S3_Table_S1-S5_Characteristics_of_the_included_studies.pdf]

Characteristics of included systematic reviews, Table S1:

Review (First author, year)	Title	Date of search, databases searched	Number of included trials (number of participants included)	Review question/ objective	Trial designs included	Exclusion criteria
Aguiar 2017	Preventing vitamin D deficiency (VDD): a systematic review of economic evaluations	Date of search: January 2015, updated: April 2016<br><br>Databases searched: MEDLINE, EMBASE, Econlit, NHS EED, CEA, and RepEC.	14 studies	"Systematically review and critically appraise economic evaluations of population strategies to prevent VDD."	"Full economic evaluations, defined as a consideration of both costs and outcomes together"	VDD occurred as secondary to other health conditions, such as liver disease or malabsorption
Al Khalifah 2020	The impact of vitamin D food fortification and health outcomes in children: a systematic review and meta-regression	Date of search: May 2019<br><br>Databases searched: Medline, Embase, Global Health, and Cochrane (CENTRAL) databases	20 RCTs (15 parallel RCTs and 5 cluster RCTs) (n=5358)	"To assess the effectiveness of utilizing vitamin D fortification in staple foods to improve 25hydroxyvitamin D (25(OH)D) concentration and to reduce the prevalence of vitamin D deficiency among healthy children"	RCTs	- Wrong comparator,<br>- duplicate, secondary report of included study,<br>- wrong intervention,<br>- wrong study design,<br>- wrong outcomes,
Brandão-Lima 2019	Vitamin D food fortification and nutritional status in children: A systematic review of randomized controlled trials	Date of search: January 2019<br><br>Databases searched: PubMed, SCOPUS, Bireme, Lilacs, and the website ClinicalTrials.gov, grey-literature search:	5 RCTs (n=792; intervention group: n=568, control: n=224)	"To evaluate the available evidence of dairy food fortification as a strategy for maintenance or recovery of nutritional status related to vitamin D in children"	RCTs	- Animal or in vitro studies,<br>- manuscript published only in summary form, and review studies,<br>- studies including children diagnosed with diseases that compromised vitamin D metabolism,<br>- studies who offered fortified foods with vitamin

		Google Scholar and OpenThesis.				D in conjunction with other nutrients
Black 2012	An updated systematic review and meta-analysis of the efficacy of vitamin D food fortification	Date of search: December 2011<br><br>Databases searched: Ovid MEDLINE, PubMed, CINAHL, Embase, and Cochrane Central Register of Controlled Trials	15 RCTs (n=1523)	"To update the evaluation of the evidence for efficacy of vitamin D fortification, including recently published data, and to summarize key outcomes and data requirements in the context of new knowledge about recommendations for vitamin D intakes."	RCTs	- Duplication,<br>- studies with insufficient data to analyze the treatment effect compared with a control,<br>- Not a randomized intervention study,<br>-Children,<br>- Institutionalized elderly,<br>- No 25(OH)D measured,<br>- No control group,<br>- Significant difference in baseline<br>25(OH)D between groups,<br>- Control group consumed equal<br>amounts of vitamin D,<br>- Not possible to quantify vitamin D<br>intake/d
Brett 2018	Effect of Vitamin D Supplementation, Food Fortification, or Bolus Injection on Vitamin D Status in Children Aged 2-18 Years: A Meta-Analysis	Date of search: December 2016<br><br>Databases searched: Ovid, PubMed, Embase and the Cochrane Central Register of Controlled Trials	26 RCT (n=5403); 9 studies with fortified foods (n=3173)	"To investigate the mean effect of vitamin D interventions (fortified foods, supplements, bolus injections) on vitamin D status in children 2–18 y of age"	RCTs	- obese populations<br>- or with chronic diseases or<br>- disease states influencing vitamin D metabolism.<br>- Studies with no mean ± SD vitamin D status
Brooker 2022	Effect of Fortified Formula on Growth and Nutritional Status in Young Children: A Systematic Review and Meta-Analysis	Date of search: articles published 1 January 1990 - 16 June 2022<br><br>Databases searched: PubMed, Web of Science, Scopus,	12 parallel-arm design RCTs (n=4795)	"To undertake a comprehensive systematic review and meta-analysis of all randomized controlled trials conducted in apparently healthy infants 9–48 months of	RCTs	-Studies published prior to 1990<br>-Studies published in non-English language<br>-Animal studies, In vitro studies<br>-Studies in adolescents and adults

		ProQuest, and Cochrane Library		age that investigated the effect of consuming a young child formula (in comparison to cow's milk or an unfortified comparator child formula) on infant growth and nutritional status."		-Acute studies (<3-month intervention period)<br>-Observational studies, review articles, conference abstracts, protocol papers "
Cashman 2021	Individual participant data (IPD)-level meta-analysis of randomized controlled trials with vitamin D-fortified foods to estimate Dietary Reference Values for vitamin D	Date of search: May–July 2018 (final screen: July 31st 2018)<br><br>Databases searched: PubMed, Ovid Medline and Embase, ClinicalTrials.gov, Cochrane Central Register of Controlled Trials (CENTRAL), and the International Standard Randomized Controlled Trials Number (ISRCTN) registry	11 RCTs (n=1429)	"The aims of the present work were firstly, through the process of a systematic review, to identify RCTs with vitamin D3-fortifed foods and subsequently use their individual data to undertake a priority IPD meta-regression analysis of the response of winter serum 25(OH)D to total vitamin D3 intake in both children and adults. Secondly, to compare our IPD-derived vitamin DRV estimates based on vitamin D3-fortifed food RCTs with international DRV which were largely based on vitamin D3-supplement RCTs; as well as comparing these estimates with those from our previous IPD (also based on vitamin D3-supplement RCTs)."	RCTs	- in infants (0–12 months) and young toddlers (12–23.9 months),<br>- pregnant or lactating women, and - dark-skinned individuals (defined as those with a Fitzpatrick skin type of V or VI).<br><br>- vitamin D3-fortifed/enhanced/enriched food(s) less frequently than weekly (e.g., monthly, quarterly, annually)<br><br>- duration <6 weeks<br><br>- RCTs that had not assessed habitual vitamin D intake in study participants

Cranney 2007	Effectiveness and safety of vitamin D in relation to bone health	Date of search: articles published from 1966 to June 2006<br><br>Databases searched: MEDLINE(R); Embase; CINAHL; AMED; Biological Abstracts; and the Cochrane Central Register of Controlled Trials	13 RCTs (n=1281 participant, intervention group: n=697, control group: n=584)	Does Dietary Intake from Foods Fortified with Vitamin D Affect Concentrations of Circulating 25(OH)D?	RCTs	- no information about the vitamin D content of the dietary source
Cranney 2008	Summary of evidence-based review on vitamin D efficacy and safety in relation to bone health	Date of search: articles published from 1966 to June 2006<br><br>Databases searched: MEDLINE, EMBASE, CINAHL, AMED, Biological Abstracts, and The Cochrane Central Register of Controlled Trials	11 RCTs	2) Does food fortification, sun exposure, or vitamin D supplementation affect circulating concentrations of 25(OH)D?	RCTs	Not reported
Das 2013	Micronutrient fortification of food and its impact on woman and child health: a systematic review	Date of search: 1 November 2012<br><br>Databases searched: MEDLINE, PubMed, POPLINE, Literatura Latino Americana em Ciências da Saúde, Cumulative Index to Nursing and Allied Health Literature, Cochrane Library, British Library for Development Studies at the International	Children: 7 RCTs, 1 CCT and 2 before-after studies<br><br>Women: 13 RCTs, 1 before-after study	"To assess the effectiveness of food fortification with single micronutrients (iron, folic acid, vitamin A, vitamin D, iodine, zinc) as well as MMN when compared with no fortification on the health and nutrition of women and children."	RCTs, CCTs and before-after studies	- home fortification with micronutrient powders, food contents, intake levels, bioavailability, - comparisons between different food vehicles or - comparisons among compounds of the same micronutrient,<br>- comparisons between fortification and supplementation, - bio-fortification and - studies evaluating the sensory impacts of fortification

		Development Statistics, WHO regional databases and the IDEAS database of unpublished working papers, Google and Google Scholar				
Dunlop 2021	Vitamin D Food Fortification and Biofortification Increases Serum 25-Hydroxyvitamin D Concentrations in Adults and Children: An Updated and Extended Systematic Review and Meta-Analysis of Randomized Controlled Trials	Date of search: December 2018, updated: 25 September, 2020"<br><br>Databases searched: CINAHL, MEDLINE, PubMed, Embase, the Cochrane Library, and gray and unpublished literature sites: WHO, Open Grey, MedNar, and NTIS	34 publications (n=2398 adults: n=1345 intervention, 1053 controls; n=1532 children: n=970 intervention, n=562 controls)	"We investigated the effect of vitamin D food fortification and biofortification on circulating 25-hydroxyvitamin D (25(OH)D) concentrations. "	RCTs	- clinical illness that may affect gut integrity/vitamin D absorption,<br>- duration <4 weeks,<br>- no equivalent placebo for control group,<br>- no quantification of daily dose or total intake,<br>- fortification dose <5 µg/d,<br>- no baseline or endpoint 25(OH)D measurement<br>- nonrandom allocation,<br>- nonblinding of participants.
Emadzadeh 2022	The Effects of Vitamin D Fortified Products on Bone Biomarkers: A Systematic Review and Meta-Analysis	Date of search: Jan 2020<br><br>Databases searched: PubMed/Medline, ISI web of knowledge, Cochrane Library, Science Direct, and Scopus, Google scholar	40 RCTs	"To assess the effectiveness of vitamin D fortification on special bone biomarkers."	RCTs	- book chapters, editorials, review articles and abstracts presented in congresses (without any full texts),<br>- studies with multi-nutrient fortified product in the intervention group, supplementation<br>- use of fortified product in the control group.<br>- follow up < 1 month,<br>- non-English articles,<br>- irrelevant outcomes
Emadzadeh 2020	The effect of vitamin D fortified products on anthropometric indices: A	Date of search: end of 2019	20 RCTs (n=2297, intervention: n=1146,	"To assess the impact of vitamin D fortified food on weight, body mass	RCTs	- multi-nutrient fortification/ other micronutrients

	systematic review and meta-analysis	Databases searched: PubMed/Medline, ISI Web of Knowledge, ScienceDirect, Scopus, Cochrane Library and Google Scholar”	control: n=1151)	index (BMI), fat mass, waist circumference (WC), hip circumference (HC) and waist to hip ratio (WHR)”		- supplementation,<br>- not used unfortified or regular diet as a control group,<br>- no anthropometric outcome,<br>- congress abstract/ no full access/ republished articles<br>- not English articles,
Emadzadeh 2020	A systematic review and meta-analysis of the effect of Vitamin D-fortified food on glycemic indices	Date of search: September 2018, last search: 25 November<br><br>Databases searched: PubMed/Medline, ISI Web of Knowledge, Cochrane Library, Science Direct, Scopus and Google Scholar	11 RCTs (n=1070, intervention: n=532, control: n=538)	“The aim of this systematic and meta-analysis was to evaluate effects of Vitamin D fortification on indices of glycemic control”	RCTs	- conference papers, book chapters and reviews<br>- Foods fortified with multinutrients /other nutrients<br>- irrelevant control<br>- not English articles were excluded
Fonseca Santos 2022	Role of food fortification with vitamin D and calcium in the bone remodeling process in postmenopausal women: a systematic review of randomized controlled trials	Date of search: 28 July 2020<br><br>Databases searched: PubMed, Lilacs, Scopus, and Bireme databases, grey-literature search: OpenThesis and Google Scholar	5 RCTs (1 of which was a crossover)	“To discuss effects of the intake of vitamin D–fortified foods in isolated form or associated with calcium on bone remodeling in postmenopausal women.”	RCTs	- Observational, in vitro or animal, and review studies, manuscripts published only in summary form
Gasparri 2019	Is vitamin D-fortified yogurt a value-added strategy for improving human health? A systematic review and meta-analysis of randomized trials	Date of search: not reported<br><br>Databases searched: PubMed, Scopus, and Google Scholar	9 studies (n=665 participants, intervention n=322, control n=343)	What are the health outcomes associated with vitamin D-fortified yogurt consumption?	RCTs	- Studies in children<br>- studies not randomized<br>- no control group
Lam 2016	Micronutrient Food Fortification for Residential Care: A Scoping Review of Current Interventions	Date of search: articles published from 2000 to December 2012, updated: April 2015	5 studies (n=181 participants)	“To (1) explore the available evidence on the efficacy of micronutrient	RCTs and studies with pretest/posttest 1-group design	- studies with multiple participant groups (e.g., community, retirement, and LTC), -if results were

		Databases searched: Ovid MEDLINE, Ovid EMBASE, EBSCO CINAHL, and Web of Science.		fortification in residential care, (2) determine which nutrients and foods could successfully be used for food fortification, and (3) identify gaps that need to be addressed with further research."		merged across sectors, (unless most participants from residential care)<br>- if studies did not evaluate the intervention,<br>- used ONS in an arm of the study<br>- no biomarkers as an outcome to determine efficacy.<br>- Non-English publications
Niedermaier 2021	Potential of Vitamin D Food Fortification in Prevention of Cancer Deaths-A Modeling Study	Date of search: February 2021<br><br>Databases searched: PubMed	10 articles (n=1303 from RCTs, n=6134 and 4051 from observational pre-post design, n=665 from a meta-analysis)	Can vitamin D food fortification achieve similar increases in vitamin D levels as vitamin D supplementation at doses that were found to be effective in reducing cancer mortality?<br><br>What would be the costs for such food fortification, and how would they compare with saved costs from prevented cancer deaths?	RCTs, observational pre-post design, meta-analysis	- Non-English-language articles,<br>- comments, correspondences,<br>- studies not reporting sufficient details on the key parameters of interest (baseline serum levels, daily uptake, change in serum levels, definition of intervention and control group)
Nikooyeh 2018	Efficacy of Food Fortification with Vitamin D in Iranian Adults: A Systematic Review and Meta-Analysis	Date of search: June 2018<br><br>Databases searched: MEDLINE, PubMed, EMBASE, and The Cochrane Central Register of Controlled Trials	5 studies (n=189)	"The objective of this study was to evaluate whether food fortification with vitamin D has the potential to increase serum 25(OH)D concentrations in Iranian people"	RCTs	- participants other than Iranian;<br>- publications not in English/Persian

Nikooyeh 2022a	How Much Does Serum 25(OH)D Improve by Vitamin D Supplement and Fortified Food in Children? A Systematic Review and Meta-Analysis	Date of search: articles published from January 2010 to October 2020<br><br>Databases searched: PubMed, Scopus, Web of Science (WoS), and Cochrane Database of Systematic Reviews (Cochrane Library, CDSR)	31 studies (n=7593 participants, intervention group: n=4583, control: n=3010);<br>11 trials compared the efficacy of vitamin D-fortified food versus unfortified, food	"This study was conducted to respond certain important questions regarding the efficacy of vitamin D fortification and supplementation in children using a meta-analytical approach"	RCTs	- Incomplete data;<br>- duplicate publication of articles;<br>- obscurely reported outcomes, or lack of control groups; and<br>- animal as well as non-interventional studies
Nikooyeh 2022b	The effects of vitamin D-fortified foods on circulating 25(OH)D concentrations in adults: a systematic review and meta-analysis	Date of search: articles published from January 2000 to July 2020<br><br>Databases searched: PubMed, Scopus, Web of Science (WoS), Cochrane Database of Systematic Reviews (Cochrane Library, CDSR) and Google Scholar	23 studies (n=2002 participants, intervention group: n=1173, control group: n=829)	"To evaluate whether vitamin D-fortified products can be a suitable solution for tackling vitamin D deficiency. Our secondary objective was to determine the effect of some variables including age, latitude and BMI on efficacy of this strategy"	RCTs	- incomplete data;<br>- duplicate publication of articles;<br>- obscurely reported outcomes, or lack of control groups; and<br>- non-interventional studies
O'Donnell 2008	Efficacy of food fortification on serum 25-hydroxyvitamin D concentrations: systematic review	Date of search: June 2006<br><br>Databases searched: MEDLINE, Embase, CINAHL, AMED, Biological Abstracts, and the Cochrane Central Register of Controlled Trials (CENTRAL).	9 RCTs - 8 parallel design, and 1 factorial design (n=889, intervention group: n=437, control group: n=452)	"To assess the effect of vitamin D–fortified foods on circulating concentrations of 25(OH)D. The secondary objective was to determine the effect of individual characteristics, such as age, BMI, ethnicity, and baseline vitamin D status."	RCTs	- not an English-language publication, - review publication,<br>- no specific study questions,<br>- lacked outcomes of interest,<br>- not human study,<br>- not quantified the amount of vitamin D in the prescribed intervention
O'Mahony 2011	The potential role of vitamin D enhanced foods in improving vitamin D status	Date of search: May 2011	9 studies (n=850)	"To provide an overview of the vitamin D containing foods	RCTs	- Not reported<br><br>- Non-English publication

		Databases searched: PubMed and Medline		available, to include natural, fortified and enriched sources" It will describe the randomized controlled trials that have been performed with vitamin D enhanced foods and the effectiveness of these in raising serum 25(OH)D levels		
Soto-Mendez 2019	Role of Functional Fortified Dairy Products in Cardiometabolic Health: A Systematic Review and Meta-analyses of Randomized Clinical Trials	Date of search: from 15 March to 30 April 2018<br><br>Databases searched: MEDLINE and SCOPUS	2 studies with vitamin D fortification (in total 41 studies; Phytosterols fortification: 31 studies, FAs: 8) (fortification: n=262, intervention n=104)	Does the intake of fortified dairy products have any effect on biomarkers of cardiometabolic risk among subjects of all ages, healthy, at risk of disease or nutritional deficiencies, with any acute or chronic diseases?	RCTs	- fortification with probiotics.<br>- RCTs published in another language than English or Spanish
Souza 2022	Vitamin d-fortified bread: Systematic review of fortification approaches and clinical studies	Date of search: from January 2000 to July 2020 (in abstract: between January 2000 and March 2021)<br><br>Databases searched: PUBMED, WEB of SCIENCE and SCOPUS databases	20 articles, including 10 Clinical trial studies	The main objective: investigating the vitamin D fortification in breads (inclusion criteria). In addition, it is important to highlight that identifying the form and dosage of vitamin D used, the type of bread flour, and the effects on health were the primary questions that guided this systematic review.	Clinical trial studies (n=10),	- Abstracts, review articles, scientific opinion and those that did not have the availability of the full text<br><br>- Non-English publication
Tangestani 2020	Efficacy of vitamin D fortified foods on bone mineral	Date of search: October 2017	20 trials (n=1786)	To summarize the efficacy of vitamin D	interventional studies	- reviews, cellular and molecular studies, animal

	density and serum bone biomarkers: A systematic review and meta-analysis of interventional studies	Databases searched: PubMed and Scopus, reference lists of articles		fortified foods on serum 25-hydroxyvitamin D (25(OH)D), bone mineral density and bone biomarkers		studies, observational studies, crossover studies, and case reports, study protocols,<br>- conference papers, letters, notes, books, and editorials,<br>- non-English studies<br>- studies with population that affect the vitamin D metabolisms like pregnant women, hypo-and hyperthyroidism, hypo-and hyperparathyroidism, renal diseases, and cirrhosis,<br>- studies with irrelevant content or unavailable,<br>- fortification of breast milk,<br>- studies conducted in mental retardation population,<br>- multi nutrient fortifications
Whiting 2015	Moderate amounts of vitamin D3 in supplements are effective in raising serum 25-hydroxyvitamin D from low baseline levels in adults: a systematic review	Date of search: articles published between 2003 and 2013<br><br>Databases searched: Medline database	18 publications with 25 separate comparisons (17 supplement studies and 1 study of fortified food)	Aim: to examine whether modest daily doses (5–20 µg) as found in fortified foods or multivitamin supplements had a measurable impact on vitamin D status,<br><br>A secondary objective was to examine whether 10 µg made a demonstrable impact on vitamin D status defined as moving from	RCTs, meta-analyses of RCTs	- not human studies;<br>- review articles;<br>- non-relevant outcomes,<br>- studies lacking blood level data pre- and post-treatment;<br>- no control group;<br>- bolus treatments (weekly, monthly, yearly);<br>- vitamin D <5 µg or >20 µg;<br>- baseline 25(OH)D ≥75 nmol/L;<br>subjects not defined as healthy (cancer, diabetes, kidney failure, HIV);

				below to above 50 nmol/L, or from less than 30 nmol/L to above 30 nmol/L		- studies <8 weeks; and<br>- age <19 years.<br>- Not an English or French language publication

Characteristics of included systematic reviews, Table S2:

Review (First author, year)	Participants (age, specific conditions etc.) - eligible to be included	Setting (countries, season)	Estimation of other vit D intake (sunshine, diet, vit D supplementation)	Intervention and comparison	Relevant outcomes	GRADE assessment of relevant outcomes Method used to assess risk of bias
Aguiar 2017	No limitations to the type of participants	"Studies were carried out in the UK, the USA, Australia, France, Finland, Sweden, and Germany. Additionally, there was one multi-country study that carried out cost-effectiveness analyses in seven European countries."	Not reported	Interventions: administering vitamin D alone, vitamin D in combination with calcium or multi-vitamin supplements containing vitamin D.	- fractures (n=8)<br>- falls (n=5)	Not reported
Al Khalifah 2020	children	"The RCTs were conducted in Canada, China, Denmark, Germany, India, Iran, Mongolia, Morocco, New Zealand, Sri Lanka, Sweden, the UK, and the USA"<br><br>"The trials were conducted at 7–56° altitude."<br><br>**Setting:**<br>school, clinical and community	"17% of pre-school children consume vitamin D supplements regularly,"<br><br>"a meta-analysis of 18 RCTs on vitamin D supplementation in adults reported low compliance"	Fortification of milk, cereal, juice, bread, yogurt, and cheese with vitamin D<br><br>compared with no food fortification or placebo for any period of time.	- serum 25(OH)D concentration<br>- prevalence of vitamin D deficiency,<br>- school performance,<br>- cognitive function,<br>- school absences,<br>- infection rate,<br>- hospital admission length when children required admission because of acute illness acquired during the trial, and<br>- compliance with the intervention	Cochrane RoB tool

Brandão-Lima 2019	Children ages 2-11 years, both sexes	The studies were conducted in 4 different countries: 2 in Canada (latitude>40° N), others in Sweden (latitude 55◦ N e 63◦ N), Germany (latitude > 50◦ N), and Mongolia (latitude 48◦ N)<br><br>2 studies were conducted exclusively during a period with no efficient sun exposure (autumn and winter), and 2 other studies also included spring; only 1 study covered all seasons	Skin pigmentation was evaluated in 4 studies (3 according to Fitzpatrick's scale), 2 studies used spectrophotometer: more than 50% of the children evaluated had types I to III skin. 1 study did not evaluate this information.<br><br>4 studies also considered the usual intake of vitamin D: with semiquantitative food frequency questionnaire (FFQ), short FFQ, and the combination of 24-h dietary recall (24HR) and 13-item semiquantitative FFQ	Food fortified with vitamin D<br><br>versus unfortified food	- serum 25(OH)D concentration	Cochrane Collaboration's tool
Black 2012	adults	Not specified.<br>"Studies that were vitamin D focused accounted for season; 7 were conducted at latitudes =40° north"	"Sunlight exposure was reported in 4 studies"	Foods fortified with cholecalciferol or ergocalciferol"<br><br>versus vitamin D-unfortified food or regular diet	- serum 25(OH)D concentration	Jadad Scale
Brett 2018	Healthy children aged 2–18 y; Study populations with vitamin D deficiency were included if children were otherwise healthy	"Location (20 of 26 trials were in locations at ≥40oN or S) of the interventions were highly variable; only 1 trial was conducted in the southern hemisphere"	"Latitude (…) did not significantly affect the change in serum 25(OH)D per 100 IU vitamin D/d"	Fortified foods<br><br>vs. unfortified foods	- serum 25(OH)D concentration	- The Cochrane qualitative bias tool<br>- and Jadad 5-point Scale

Brooker 2022	Apparently Healthy Children Aged 9-48 Months at Trial Commencement	"Three studies were conducted in Latin America, three in Asia, three in Europe, two in the Pacific region, and one study was a multi-center trial across several countries in Asia and Europe"	Not reported	Fortified milk or formula (with iron, vitamins C and D, zinc, iodine, prebiotics, probiotics, and essential fatty acids)<br><br>vs non-fortified milk or formula	- serum 25(OH)D concentration<br>- growth parameters and/or<br>- biochemical markers (hemoglobin, ferritin, transferrin, soluble transferrin receptor, plasma zinc, urinary iodine), energy-, protein-, and iron intake	Cochrane Risk of Bias Tool 2.0 for intervention studies
Cashman 2021	Populations of interest: male and female children and adults	winter<br><br>"The RCTs were conducted in 8 countries within North America and Europe."	"The foods were to be consumed as part of a diet and not oral supplements"	Intervention: vitamin D3 consumed orally as a fortified /enhanced /enriched food(s) and taken daily or weekly<br><br>vs unfortified food with vitamin D	- Serum 25(OH)D concentration	- Jadad scale<br>- the Cochrane Collaboration's tool
Cranney 2007	No limitations to the type of participants	Countries:<br>Ireland, US, Australia, Netherlands, China, Spain, Italy, Finland, Malaysia	"Sunlight exposure was assessed in only three of the 11 trials although several others excluded subjects who had recent or planned exposure to higher-than-usual levels of sunshine. Methods of ascertainment included a sunlight exposure score during the summer in a subsample, the	Food fortified with vitamin D<br><br>vs. non-fortified food or usual diet	- Serum 25(OH)D concentration	Jadad scale

			percentage of participants who were outside daily during sunny period and the percentage who avoided sunlight and an outdoor score to reflect the average exposure to sunlight per day per season. Results showed that sunlight exposure did not predict post therapy serum 25(OH)D in the total sub-sample, that there was no significant difference in sunlight exposure between groups at baseline or during the study. “			
Cranney 2008	age groups (infants, children, women of reproductive age, and older men and women)	Not reported	Not reported	vitamin D2 or D3 (with or without calcium) versus control	- serum 25(OH)D concentration	Jadad scale
Das 2013	- Infants, children and adolescents of all age groups (ages 2 to 18 years)<br>- Women (of reproductive age and post-menopausal)	In “Low-income, lower middle-income, Upper middle-income and higher-income countries”<br><br>Children:<br>“Twenty-two of the studies were carried out in upper middle-income countries (UMIC) and higher	Not reported	Fortified food with vitamin D<br><br>vs unfortified food	- Serum 25(OH)D3 concentration<br>- alkaline phosphatase and<br>- serum PTH (parathyroid hormone)	Cochrane Collaboration’s tool

		income countries (HIC), and 18 were from lower middle-income countries (LMIC) and lower income countries (LIC).”<br><br>Women:<br>“Ten of the studies were carried out in UMIC/HIC and three studies were from LIC/LMIC.”				
Dunlop 2021	Children and adults, participants without compromised vitamin D absorption	“Studies were conducted in 18 different countries across 4 continents: Asia, Europe, North America, and Oceania, spanning latitudes of 3–60°”<br><br>22 publications concluded their intervention in winter/spring, whereas 6 concluded in summer/autumn, seasons of intervention were not specified in 6 publications	“Potentially confounding factors (e.g. (…) sun exposure, (...), for which sufficient data were unavailable, may have influenced outcomes”	Fortified food with vitamin D<br><br>compared with unfortified food	- Serum 25(OH)D concentration	Jadad scale
Emadzadeh 2022	No limitations to the type of participants	Studies were from fourteen different countries.	Not reported	Fortified food with vitamin D	- Serum 25(OH)D concentration<br>- serum PTH<br>- serum IGF-1	Jadad scale

				vs unfortified food (or a regular diet) for at least one month	- serum telopeptides of type-1 collagen (C-terminal: CTx)<br>- serum osteocalcin<br>- Bone Mass Density (BMD) such as total, femoral neck, and lumbar BMD	
Emadzadeh 2020/a	No limitations to the type of participants with respect to age, sex, and anthropometric indices	"Eight studies were from Iran, four studies were from the USA, two studies from Canada, Australia and Greece, and one each from China and Denmark"<br><br>The season of the year during which the intervention was undertaken were not addressed in all the studies included	Not reported	Fortified food with vitamin D (or calcium plus vitamin D)<br><br>compared with placebo (regular diet)	Anthropometric indices, including:<br>- body mass index,<br>- total body weight,<br>- fat mass,<br>- lean mass,<br>- waist circumference and<br>- hip circumference	Cochrane Collaboration risk of bias tool"
Emadzadeh 2020/b	No limitations	From 11 studies, 9 studies were based in Iran, 1 in the United States, and 1 in Canada.	Not reported	"Vitamin D-fortified food or calcium–Vitamin D (Ca–D)-fortified food<br><br>versus regular diet (or using nonfortified food)"	Glycemic status:<br>- HbA1C,<br>- fasting serum glucose (FSG),<br>- fasting serum insulin (FSI) and<br>- homeostatic model assessment-insulin resistance (HOMA-IR))	Jadad scale
Fonseca Santos 2022	Postmenopausal women	France (4 RCTs), Switzerland (1 RCT)	Not reported	Intervention: "Intake of fortified food with vitamin D, either isolated or associated with calcium"	- serum 25(OH)D and/or<br>- 1,25(OH)2D<br>- serum PTH concentrations;	Cochrane Collaboration tool for bias risk assessment

				Comparison: “Intake of nonfortified foods”	- bone resorption and/or formation markers (CTX, TRAP5b, and P1NP)	
Gasparri 2019	women and men aged 18 to 99 years	Not reported	Not reported	Vitamin D-fortified yogurt<br><br>compared with plain yogurt	- Serum 25(OH)D concentration<br>- anthropometric parameters:<br>- body weight,<br>- waist circumference and<br>- body mass index;<br>- blood pressure,<br>- lipid profile (total cholesterol, LDL, HDL, TG) and<br>- glucose metabolism (fasting serum glucose, fasting serum insulin)	Cochrane risk of bias tool
Lam 2016	The study sample was from residential care”	“Studies were conducted in France (n = 3 studies), with 1 study conducted each in Romania, Canada, and Spain.”	Not reported	Food fortified with vitamin D. Both controlled and uncontrolled trials included.<br><br>Comparator not specified.	- Serum 25(OH)D concentration<br>- serum calcium<br>- serum PTH<br>- Bone mineral density (BMD)<br>- quality of life (QOL)	Not reported
Niedermaier 2021	No limitations	2 study from Ireland, 1 from India, 2 from Finland, 1 from China, 1 from Iran, 1 from USA, 1 from Denmark	Not reported	Vitamin D fortified food<br><br>vs placebo	- cancer mortality,<br>- serum 25(OH)D concentration<br>- costs of vitamin D fortification (savings from prevented cancer deaths and fracture costs),	Not reported

					- preventable years of life lost	
Nikooyeh 2018	Iranian adult participants	Iran	Not reported	fortified food either with vitamin D2 or D3 (with or without calcium)<br><br>versus control	- serum 25(OH)D concentrations	- Cochrane risk of bias tool and<br>- Oxford quality scoring system (Jadad scale)
Nikooyeh 2022a	children from 1 to 18 years of age	"Thirteen (trials) from Asia, eight from America and Canada, eight from Europe and Australia, and two from Africa".<br>No specific data for the 11 fortification studies	"Latitude of location (B 0.04, 95%CI –0.26, 0.35, P = 767) did not significantly influence the between-group difference of 25(OH)D levels"	vitamin D-fortified food<br><br>versus the same, but unfortified, food	- serum 25(OH)D concentrations	Cochrane risk of bias tool
Nikooyeh 2022b	adults aged 18 years and older	"Of the twenty-three studies included, four were from North America including Canada, six from Asia and the rest from Europe and Australia"	"Most of the trials were conducted in countries located in latitude higher than 35°. The treatment effect was better in trials from countries located in lower than 35°. However, no differing patterns were clearly evident between these subgroups ($\chi 2$ 3·65, P 0·06)" "latitude of location (P = 0·096) did not significantly influence the between-group difference of 25(OH)D concentrations"	foods fortified with vitamin D2 or vitamin D3 alone or in combination with Ca<br><br>vs. unfortified foods or no intervention	- Serum 25(OH)D concentration	Cochrane risk of bias tool

O'Donnell 2008	all populations, community-dwelling participants	2 trials – USA,<br>2 trials - Ireland<br>others from Malaysia, Australia, Netherlands, China, Finland<br><br>Season of sample:<br>3 trials – winter<br>1 trial – spring<br>1 trial - Late winter (baseline) and summer (end of study)<br>4 trials – not reported	Vitamin D dietary intake was evaluated at baseline in 3 trials. During the intervention, the total intake of vitamin D from diet was reported in one trial, and that from diet and supplements was reported in another trial.<br>7 trials excluded participants with regular vitamin D/ multivitamin suppl.<br>Sun exposure was assessed in 3 trials.	foods fortified with vitamin D<br><br>vs no intervention, regular diet, or unfortified foods (milk/orange juice/cheese/bread) or regular food + vit. D3 supplement	- serum 25(OH)D concentrations	Jadad scale
O'Mahony 2011	No limitations	Most of the interventions were performed during winter months, when the cutaneous synthesis of vitamin D is low and does not contribute to circulating levels of 25(OH)D	Most of the interventions were performed during winter months, when the cutaneous synthesis of vitamin D is low and does not contribute to circulating levels of 25(OH)D	Fortified food<br><br>vs usual diet/ unfortified food/ supplement/ no intervention	- Serum 25(OH)D concentration<br>- serum PTH<br>- serum calcium concentration	Not reported
Soto-Mendez 2019	Humans of all ages and stages of life, including healthy subjects; subjects at risk of disease or nutritional deficiencies; subjects in different physiological stages (e.g., pregnancy, menopause,	Not reported	Not reported	fortified milk or dairy products<br><br>compared to controls (non-exposed), placebos (unfortified products or products	Biomarkers of cardiometabolic health:<br>- TC,<br>- LDL cholesterol,<br>- TG, and<br>- blood pressure	- Certainty of evidence assessments were performed with use of the GRADE Pro GDT application.

	etc.); and subjects with any acute or chronic disease			with a regular content of the nutrient of interest), or another treatment group (different levels of exposure or another vehicle of fortification)		- Risk of bias assessment "according to Cochrane guidelines "
Souza 2022	No limitations	Finland, Germany, United Kingdom, Denmark, Belgium and Ireland,<br><br>4 studies: fortification during the winter (theoretical models: 2, clinical studies: 2)	Studies used data from national nutrition surveys in their respective countries (RDA and UL, information about seasonality sun exposure to vitamin D and the 25(OH)D serum status in the population)<br><br>One included study considered the synthesis of vitamin D in the skin	Fortified wheat bread<br><br>vs regular wheat bread,<br><br>1 study – Vitamin D2 vs Vitamin D3 fortified bread	- Serum 25(OH)D concentration	Not reported
Tangestani 2020	Healthy population, without age restriction,	studies were conducted in England (1), French (3), Greece (5), Spain (1), Romania (2), Australia (2), Iran (3), Japan (1), and China (2)<br><br>sample size ranges from 40 to 429.	Not reported	Vitamin D fortified foods (with or without calcium).<br><br>Comparator not specified.	- any fractures,<br>- Serum 25(OH)D concentration<br>- bone mineral density (BMD) of femoral neck or lumbar spine,<br>- serum parathormone (PTH), and<br>- bone turnover markers (BTM) like OC, ALP, P1NP, and CTX.	- Jadad scale<br><br>- GRADE not reported
Whiting 2015	Healthy adults	5 studies - USA, 2 from France, 2 from Finland, others are	1 fortification study – conducted in winter time with limited sun	Vitamin D fortified food/supplement	- Serum 25(OH)D concentration	Not reported

		from Denmark, Scotland, Chile, Ireland, Netherlands, Bangladesh, Germany and Austria, Brazil<br><br>1 fortification study – Female population in France, Institutionalized, BMI = 26, winter time, limited sun exposure	exposure, no supplementation	vs control group (placebo-controlled or having no placebo pill)		

Characteristics of interventions, Table S3:

Review (First author, year)	Intervention	Prevention or treatment	Population (mean age, specific condition)	Type of food used as vehicle	Dose of fortification	Vit D compound (D2/D3)	Intervention duration and follow up	Compliance (dropout rates, any way compliance is estimated)
Aguiar 2017	Bread fortification with vitamin D (800 IU) + calcium (200 mg)	Prevention	Women over 65 years in Germany	Bread	800 IU	Not specified	Not reported	Not reported
Al Khalifah 2020	fortification of milk, cereal, juice, bread, yogurt, and cheese with vitamin D	Prevention	Age: 1.4 to 18 years	- milk,<br>- cereal,<br>- juice,<br>- bread<br>- two items of food: yogurt and cheese or milk and bread	60 -1000 IU/day	11 trials: Vitamin D3,<br><br>9 didn’t report	Range 2–24 months,<br><br>Median intervention duration: 5 months	***“Compliance was defined differently in the studies.*** “<br><br>“In our review, compliance was similar among fortified and unfortified food products. "<br><br>Lost to follow-up: 91/395, 14/85, 9/39, 3/77, 2/51, 59/757, 34/176, 0/172, 0/60, 44/225, 39/92, 63/776, 3/227, 0/321, 13/146, 7/170, 17/206, 5/78,

								0/278, 0/294,64/360
Brandão-Lima 2019	Vitamin D-fortified foods	Prevention	- children of both sexes,<br>- Age: 2 - 11 years.<br>- 3 studies -Normal weight children.<br>- 2 studies - Children with normal weight, thinness, overweight, and obesity.<br>- Skin pigmentation evaluation in 4 studies (3 according to Fitzpatrick's scale),<br>- 2 studies used spectrophotometer: more than 50% of the children had types I - III skin.<br>- 1 study did not evaluate this information.<br><br>- Vitamin D status at baseline was variable, with children at risk of deficiency (<30 nmol/L) and of insufficiency (30–49 nmol/L), and with status classified as	cheddar cheese, yogurt, milk	42 - 880 IU/serving<br><br>300–880 IU/ day	Vitamin D2/D3	1.6 - 9 months	Not reported

			sufficiency (≥50 nmol/L)<br>- 1 study - almost all children: at risk for vitamin D insufficiency and deficiency					
Black 2012	Foods fortified with Vitamin D2 or D3	Prevention	- Adults; age range 18-91 years;<br>- healthy or diabetic (in 2 trials);<br>- 6 studies were conducted in women only and 2 in men only	- dairy products (12)<br>- orange juice (2)<br>- bread (1)	120-1000 IU/ 100 g or serving<br><br>8 studies: ≤400 IU	Not specified	11 weeks - 2 years	"All studies reported data on dropouts; 3 had a dropout rate of >15% " "The compliance rate was reported in 10 studies". "Compliance was not reported in 5 studies and was extremely low in one (45% at the final time point)"
Brett 2018	Fortified food with vitamin D	Prevention	- Healthy children 2–18 Years,<br>- 3 trials only in boys and<br>- 4 trials only in girls"	- cereal–based food,<br>- milk,<br>- bread,<br>- orange juice,<br>- yogurt/cheese	100 – 1000 IU /day	Vitamin D3/ not reported	1.6 month - 2 years	"Drop-out percentage was reported in 17 of 26 trials: (range: 1–22%)"<br>10 had average compliance >80%

								13 of 26 trials did not report compliance
Brooker 2022	Fortified milk or formula with vitamin D	Prevention	- All studies: mixed-sex populations; sex ratio: range 39% to 57% males.<br>- age: from 9 months to 48 months. - most studies: ‘apparently healthy’ young children, (studies varied in their classification of ‘healthy’, or did not elaborate on this criterion	Milk,<br>milk formula	The dose of milk prescribed: 150 - 750 mL/day	Not specified	20 weeks - 12 months.	Compliance rate range: 71–100%
Cashman 2021	Orally consumed Vitamin D3 as a fortified/enhanced/enriched food(s) and taken daily or weekly.	Prevention	- ages 2–89 years<br>- 7 studies in adults,<br>- 3 in children,<br>- 1 in both age-groups.<br>- 3 studies in adult females only,<br>- the rest: males and females.	- cheese or a cow’s milk-based beverage (5)<br>- yoghurt and cheese (1),<br>- bread (1);<br>- eggs (1);<br>- orange juice or biscuits (1);<br>- milk plus bread (1);<br>- combination of 4 foods (vitamin D-fortified low-fat cheese, yoghurt, eggs and crisp bread)	140 – 4000 IU/day<br><br>5 studies: ≤400 IU/day<br><br>1 study: 400 and 600 IU/day<br><br>4 studies 480-1200 IU/day<br><br>1 study: 4000 IU/day	Vitamin D3	8 weeks - 6 months.	“There was a relatively low percentage of participant dropouts (0–18.5% within a study arm) and only one study had a dropout rate of >15%.”

Cranney 2007	Fortified food with vit D	Prevention	11 trials - adults<br>2 trials – young adults<br>3 trials- postmenopausal women<br>1 – elderly men<br>4 – elderly adults	- Dairy products (milk, milk powder, cheese),<br>- Orange juice,<br>- Wheat bread	Most study: 400 IU/day<br><br>2 studies: 600 or 800 IU/day<br><br>2 studies: 228 - 240 IU/day	8 trials: Vitamin D3<br><br>3 trials: did not report	3 weeks - 24 months	"Compliance was reported in 4 trials and was >85%"
Cranney 2008	Food fortified with vitamin D	Prevention	- Most of the trials: postmenopausal women or older men, >60 years<br>- few high-quality trials: infants, children, pregnant women, or lactating women.<br>- Most studies were in white populations only.	- Dairy products (in most included trials)	137–1000 IU /day	7 trials: Vitamin D3,<br><br>in the remaining trials: not specified	Not reported	Not reported
Das 2013	Fortified food with vitamin D	Prevention	7 trials Children<br>- 1 study: preterm infants<br>- others: on children and adolescents from 6 years to 18 years of age.<br><br>13 trials Women (reproductive age and post-menopausal)	- Milk<br><br>Not reported	"The amount of micronutrient used varied significantly among the studies"<br><br>"The level of fortification varied significantly"	Not specified	Not reported<br><br>2 weeks - 2 years	Not reported
Dunlop 2021	Fortified food with vitamin D	Prevention	- age: <18, ≥18 and ≥55 years in 7, 17,	- milk, milk powder, milk-	200 – 4000 IU/day	3 publications: vitamin D2	4 - 104 week	Compliance rates were

			and 9 publications, respectively.<br>- 27 studies: adults only<br>- 7 included only children.<br>- All trials: mixed sex or females only, none included only males.<br>- 19 studies healthy, free-living participants.<br>- 4 studies postmenopausal women,<br>- 3 studies: people with type 2 diabetes.<br>- 8 studies: Various conditions (frail elderly, institutionalized elderly, women in sheltered accommodation, postmenopausal women with type 2 diabetes, iron deficiency, metabolic syndrome, overweight and obesity, and gestational diabetes mellitus)	based drinks, yogurt, cheese, fruit juice, biscuits, snack bars, crisp breads and lavash bread.<br><br>- 3 studies - multiple food administration (egg, yogurt, cheese and crisp breads, and yogurt and cheese).<br><br>- 1 study provided a range of fortified foods (fruit juice, compote, custard, 2 fruit yogurt options, custard, or cheese curd with fruit) from which participants selected 2 daily	mean dose weighted by n participants = 648 IU/day;<br>- adults 756 IU/day;<br>- children 472 IU/day	and vitamin D3,<br><br>1 publication: vitamin D2.<br><br>22 publication (17 adults, 5 children): vitamin D3<br><br>8 publications (6 adults, 2 children) did not specify vitamin D,<br><br>None used 25(OH)D2 or 25(OH)D3”		reported in 24 publications, all with average rates >80%.<br><br>Compliance, was missing from almost one-third of included studies.

Emadzadeh 2022	Fortified food with vitamin D	Prevention	- 8 studies: children and adolescents. Mean age: 2 - 84 years<br>- 17 studies: only females<br>- 2 studies: only males.	- Dairy products (most studies),<br>- fortified orange juice,<br>- bread,<br>- biscuit and snack bars.	40 IU/day - 28000 IU/day	Not specified	1 - 30 months	In the Jadad scale
Emadzadeh 2020	"Vitamin D fortified foods"	Prevention	- 6 articles: age: <50 years old.<br>- 3 studies: in children (2–12 years old).<br>- 5 studies in adults >50 years old.<br>Other studies: wide age range"<br>- Racial characteristics: not addressed in all the studies	- Eggs,<br>- milk,<br>- yoghurt,<br>- cheese,<br>- orange juice,<br>- bread,<br>- snack bar	100-28000 IU/day<br><br>10 studies <1000 IU<br><br>in other studies ≥1000 IU	Vitamin D3/ not specified	2 - 24 months	Not reported
Emadzadeh 2020	Vitamin D-fortified food	Prevention	- Age: 18 to 75 years<br>- All studies were conducted on diabetic subjects<br>- Except 2 studies with healthy and prediabetic subjects	Dairy products, except 1 study: juice	Range: 1000 IU – 28000 IU/day	Vitamin D3	2 - 6 months	In 1 article participants monitored strictly and the compliance rate was about 100%.
Fonseca Santos 2022	Vitamin D and calcium-fortified food	Prevention	Postmenopausal women: Mean age ranged from 56.1 to 86.9 years.	- Yogurt (2),<br>- Cheese (1),<br>- Soft plain cheese (2)	50 - 200 IU /serving	Vitamin D3	4 - 12 weeks.	Not reported
Gasparri 2019	vitamin D-fortified yogurt + calcium	Treatment	- 3 study involved subjects with type 2 diabetes, - 1 study involved	Yogurt	Vitamin D alone: 500, 1000, or 2000 IU	Not specified	8 - 16 weeks	Not reported

			prediabetic subjects, and<br>- 2 studies involved elderly institutionalized women,<br>- 3 studies considered pregnant women with gestational diabetes mellitus at the onset of their second trimester<br>- 5 studies: men and women<br>- 4 studies: only women		or vitamin D (dose of 400 or 500 IU) plus calcium			
Lam 2016	Vitamin D ± Calcium fortified food	Prevention	- study sample was from residential care<br>- Mean age ranged from 68.3 to 87.2 years	cheese, yogurt, and buns	100 IU – 5000 IU/day	Not specified	Intervention duration: 1 - 12 months;<br>Follow-up: 1 - 24 months	"This study did not examine compliance of treatment, as this information was not included in most studies"
Niedermaier 2021	vitamin D food fortification	Prevention	- Participants' age: from school aged children to older individuals (in most of the cases: adults),<br>- healthy population	- Milk and milk products (6),<br>- bread (2),<br>- milk and bread (1),<br>- orange juice (1)	200–1040 IU /day	Not specified	8 weeks–11 years	Not reported
Nikooyeh 2018	fortified food either with vitamin D2 or D3 (with or without calcium)	Prevention	- Age: from 20 years of age<br>- 4 studies were conducted in	- Yogurt (n=1),<br>- yogurt drink (n=2),<br>- milk (n=1),<br>- bread (n=1)	1000-2000 IU /day	Not specified	8 – 12 weeks	Not reported

			participants with type 2 diabetes<br>- 1 in women only					
Nikooyeh 2022a	vitamin D-fortified food	Prevention	- Age: 1 to 18 years;<br>- all children: healthy<br>- one out of 11 studies described the included population as vitamin D deficient	Dairy products (9 out of 11 studies)<br>Orange juice<br>Cereal	80 - 1000 IU /day	Not specified	1 - 12 months	“All trials provided data on losses to follow-up". Out of the 11 fortification trials four “trials reported losses of >10%”
Nikooyeh 2022b	foods fortified with vitamin D2 or vitamin D3 alone or in combination with Ca (10 trials)	Prevention	- Adults >18 years;<br>- 12 studies - in women and 2 in men.<br>- Subjects with type 2 diabetes - in three studies. - 3 studies – with initially vitamin D insufficient participants,<br>- vitamin D status was not among the inclusion criteria in other studies”	- dairy products (21 out of 23)<br>- juice,<br>- grain product,<br>- oil and dairy together with grain products	200 - 2000 IU/day	2 trials (3 arms): Vitamin D2<br><br>Others: Vitamin D3	3 weeks – 2 years	“All trials provided data on losses to follow-up; only four reported losses of >10 %”
O'Donnell 2008	Milk or dairy products, orange juice, bread fortified with vitamin D	Prevention	- All studies: community-dwelling participants.<br>- Young adults: (mean age < 30 yrs) 3 trials<br>- older persons: 6 trials (2 trials –	- Milk or dairy products (7): milk (5), cheese (1),<br>- nutrient-dense fruit- and dairy-based products (1)<br>- Orange juice (1)	136-1000 IU/day	Vitamin D3	3 weeks – 24 months<br><br>7 studies ≥ 3 months	8 trials provided data on losses to follow-up, only 1 reported loss of >20%

			postmenopausal women)<br>- The ethnicity was reported in only 3 trials<br>- BMI was reported in 4 trials<br>- 7 trials excluded participants with regular vitamin D/ multivitamin suppl.<br>- 2 trials did not report on the use of supplements	- Bread (1)				
O'Mahony 2011	Vitamin D2/D3 fortified/ enhanced food consumption	Prevention	- volunteers were generally healthy (1 study – diabetic subjects),<br>- representing both genders<br>- and age groups ranging from 18 to 87 years	- Milk (2),<br>- cheese (2),<br>- yogurt (1),<br>- orange juice (2),<br>- UV enhanced mushrooms (1),<br>- bread (1)	400-4000 IU/day	Vitamin D2 or D3	Most of the interventions: 3 - 12 weeks,<br><br>2 studies: 24 months	Compliance, dropout rate was reported in most of the studies<br><br>1 trial - Three subjects dropped out of the study due to difficulties with compliance.<br><br>1 trial - five dropouts in the cheese group<br><br>1 trial- no dropouts<br>2 trials - compliance was good

Soto-Mendez 2019	Milk or dairy products fortified with vitamin D	Prevention	- Age: 25.6-, 28.6 years<br>- Women with gestational diabetes (in 1 study) and<br>- young women with low Fe stores (in 1 study)	Milk or dairy products	200-500 IU/day	Vitamin D3	16 weeks	Not reported
Souza 2022	Bread fortified with Vitamin D	Prevention	- 6 trials – adults (4 trials – healthy women)<br>- 1 trial - Children and adults<br>- 3 trials - elderly (2 trials – nursing home residents)	Bread	172 -5000 IU/100 g	14 studies: Vitamin D3<br><br>4 studies: Vitamin D2	3 weeks - 12 months	Not reported
Tangestani 2020	vitamin D fortified food consumption	Prevention	- 14 Studies - on women, 2 on men and 4 on both genders<br>- 16 studies - on the elderly: 7 on postmenopausal women, 2 on diabetic postmenopausal women and diabetic men, 2 on nursing homes resident, 2 community dwelling home resident, 2 on the elderly with or without vertebral fractures;<br>- 2 studies on the youth and	- Dairy products: milk (6), yogurt (4), yogurt drink (1), cheese (2) and combination of milk with yogurt (5),<br>- Orange juice (1)<br>- Bread (2)	80 - 5000 IU/day	Not specified	1 - 24 months	Not reported

			postpartum lactating women,<br>- 2 on children and urban girls					
Whiting 2015	Vitamin D and Ca++ fortified yogurt	Prevention	1 fortification study – Female population in France, Institutionalized, BMI = 26, wintertime, limited sun exposure	Yogurt	400 IU	Vitamin D3	8 weeks	Not reported

AMSTAR, Table S4

Options: Yes / No / Can't answer / Not applicable

Review (First author, year)	1 research question and inclusion criteria	2 duplicate study selection and data extraction	3 literature search in at least two electronic sources, keywords	4 publication types	5 list of included and excluded studies	6 characteristics of the included studies	7 quality of the included studies	8 quality was taken into account used in formulating conclusions	9 appropriate methods used to combine the findings	10 publication (reporting) bias assessed	11 conflict of interest included	Total score (out of max. 11)
Aguiar 2017	Yes	No	Yes	Yes	No	Yes	Yes	Yes	Not Applicable	No	No	6
Al Khalifah 2020	Yes	Yes	Yes	Yes	Yes	Yes	Yes	Yes	Yes	Yes	No	10
Brandão-Lima 2019	Yes	Yes	Yes	Yes	No	Yes	Yes	No	Not Applicable	No	No	6
Black 2012	Yes	Yes	Yes	Yes	No	Yes	Yes	No	Yes	No	No	7
Brett 2018	Yes	Yes	Yes	Yes	No	Yes	Yes	No	Yes	No	Yes	8
Brooker 2022	Yes	Yes	Yes	Yes	No	Yes	Yes	Yes	Yes	Yes	No	9
Cashman 2021	Yes	Yes	Yes	No	No	Yes	Yes	Yes	Yes	No	No	7
Cranney 2007	Yes	Yes	Yes	Yes	Yes	Yes	Yes	Yes	Yes	Yes	No	10
Cranney 2008	Yes	Yes	No	Yes	No	No	Yes	Yes	Yes	No	No	6
Das 2013	Yes	Yes	No	Yes	No	Yes	Yes	Yes	Yes	No	No	7
Dunlop 2021	Yes	Yes	Yes	Yes	No	Yes	Yes	Yes	Yes	Yes	No	9
Emadzadeh 2022	Yes	Yes	Yes	Yes	No	No	Yes	Yes	Yes	Yes	No	8
Emadzadeh 2020	Yes	Yes	No	No	No	Yes	Yes	Yes	Yes	Yes	No	7

Emadzadeh 2020	Yes	Yes	Yes	Yes	No	Yes	Yes	No	Yes	Yes	No	8
Fonseca Santos 2022	Yes	Yes	Yes	Yes	No	Yes	Yes	No	Not Applicable	No	No	6
Gasparri 2019	Yes	No	Yes	Yes	No	Yes	Yes	No	Yes	Yes	No	7
Lam 2016	Yes	Yes	Yes	Yes	No	Yes	No	No	Not Applicable	No	No	5
Niedermaier 2021	Yes	No	No	Yes	No	Yes	No	No	Not Applicable	No	No	3
Nikooyeh 2018	Yes	Can't Answer	Yes	Yes	No	Yes	Yes	No	Yes	No	No	6
Nikooyeh 2022a	Yes	Yes	Yes	Yes	No	Yes	Yes	No	Yes	Yes	Yes	9
Nikooyeh 2022b	Yes	Yes	Yes	Yes	No	Yes	Yes	No	Yes	Yes	Yes	9
O'Donnell 2008	Yes	Yes	No	Yes	No	Yes	Yes	Yes	Yes	No	No	7
O'Mahony 2011	Yes	Can't Answer	No	Yes	No	Yes	No	No	Not Applicable	No	No	3
Soto-Mendez 2019	Yes	Yes	Yes	Yes	No	Yes	Yes	Yes	Not Applicable	Yes	No	8
Souza 2022	Yes	No	Yes	Yes	No	Yes	No	No	Not Applicable	No	No	4
Tangestani 2020	Yes	Yes	Yes	Yes	No	Yes	Yes	No	Yes	Yes	No	8
Whiting 2015	Yes	Can't Answer	No	Yes	No	Yes	No	No	Not Applicable	No	No	3

Results of included reviews, Table S5:

Review (First author, year)	Comparison	Outcome	Number of studies; number of participants (for each outcome)	Results (from meta-analysis or narrative description)	Heterogeneity, further subgroup analysis	RoB results	GRADE assessment
Aguiar 2017	"Bread fortification with vitamin D (800 IU) + calcium (200 mg)<br>vs<br>No food fortification"	"Fractures" and "Cost per avoided fracture"	1 study (Sandmann et al., 2015)	"Bread fortification with vitamin D and calcium was cost saving"	Not Applicable	Not reported	GRADE not reported

Al Khalifah 2020	Fortified food products vs unfortified food, different food products, or no food	25(OH)D concentration nmol/l”	18 RCTs, including 4044 total children	MD of 15.51 nmol/L (95% CI 6.28, 24.74; I2 = 99%)”	I^2 = 99%<br>“Based on the food vehicle used, age groups, country income level, the methodological quality of the included studies, and RCT type to explain the heterogeneity.”<br>Food vehicle:<br>- Milk increased 25(OH)D concentration by an MD of 23.72 nmol/L (95% CI 22.86, 24.58; I^2 = 99%), Heterogeneity remained substantial<br>- juice increased 25(OH)D concentration by an MD of 11.80 nmol/L (95% CI 7.35, 16.26; I^2 = 0%),<br>- cereal increased 25(OH)D concentration by an MD of 8.93 nmol/L (95% CI − 0.36, 18.21; I^2 = 40%), and<br>- yogurt and cheese increased 25(OH)D concentration by an MD of 5.34 nmol/L (95% CI 0.97, 9.70; I^2 = 49%).<br><br>Trials that used milk for fortification:<br>- clear benefit among preschool and school-aged children.<br>- school-aged children had higher 25(OH)D concentration than pre-school children.<br>- no heterogeneity detected in the preschool group (I^2 = 0%), and the degree of overlap of the point estimates and CIs were homogenous, compared with those in the school-aged children (I^2 = 99%).<br><br>Country income level, differences in the methodological quality, and RCT type were not statistically significant.”	- most of the studies had a low RoB for randomization and an unclear RoB for allocation concealment<br>- Four studies had a high RoB for blinding<br>- One study had a moderate RoB for incomplete outcome data<br>- For selective outcome reporting, one study was judged to be at high RoB<br>- four out of the five cluster RCTs had a high RoB because they did not account for the cluster design effect<br>- two studies had unclear methods for RCT design<br>- In summary, three studies were determined to have a high RoB	“⊕⊕⊕⊕ High”

		vitamin D deficiency prevalence	16 RCTs, including 4093 total children	Food fortification reduced vitamin D deficiency by an RR of 0.53 (95% CI 0.41, 0.69; I2 = 94%)	I^2 = 94%		"⊕⊕⊕⊕ High"
		school performance in math, science, and social science (measured using age- and gender-standardized end-of-term test scores)	2 trials (904 participants)		Math: SMD of 0.90 (95% CI –0.39 ,2.18; I^2 = 99%)<br>Social studies: SMD of 1.20 (95% CI –1.43 ,3.84; I^2 = 100%)<br>Science: SMD of 1.44 (95% CI –0.94, 3.81; I^2 = 100%)		Math: "⊕OOO Very LOW"
		cognitive function (IQ)" - measured by Wechsler Scale of Intelligence or IQ test	3 studies (using different measurement scales)	Significantly improved cognitive function by an MD 1.22 (95% CI 0.65, 1.79)<br><br>*1 study used a scale that could not be combined with the other studies"*	Sub-group analysis not conducted		"⊕⊕⊕O Moderate"
		Infection rate and hospitalization	2 RCTs	1 study: lower chest infection rate among the vitamin D fortified group (MD − 0.35, 95% CI − 0.58, − 0.12)<br>1 study: no events of URTI or diarrhea in either group after 14 months of follow-up	Sub-group analysis not conducted		
Brandão-Lima 2019	Fortified food with vit D2/D3	Serum 25(OH)D measured in:	5 trials	3 studies: increase of 25(OH)D levels in all groups	studies showed clinical and methodological heterogeneity that could not be sufficiently	100% were classified as low	GRADE not reported

	vs Unfortified food with vitamin D	mean difference± standard deviation (nmol/L)		receiving fortified foods (Δ (nmol/l)<br>1. 30.0 +/-13.2 (Mongolian milk),<br>47.4+/-27.7 (USA milk);<br>2. 4.5 ± 11.7 (EAR),<br>3.2 ± 11.0 (RDA);<br>3. 13.0 ± 15.8 (10 ug),<br>24.0 ± 18.8 (25 ug)),<br>respective control groups did not present alteration (1. 0 ± 10.0; 2. −2.5 ± 13.4; 3. 1.0 ± 17.5)<br><br>1 study: After winter (intervention group vs control) Δ (nmol/l) 8.3 ± 23.6 vs −12.0 ± 20.0, Summer (intervention group vs control) 15.3 ± 18.1 vs 22.5 ± 18.5<br><br>1 study: 3 months (intervention group vs control) Δ (nmol/l) −0.6 ± 12.2 vs −9.2 ± 15.2;<br>6 months (intervention group vs control) Δ (nmol/l) −6.9 ± 10.9 vs −10.9 ± 14.5	explored in the subgroup analysis and could produce clinically inconsistent results<br><br>In the studies, no differences were observed between the intervention and control groups in relation to age, sex, and BMI.	risk of selection bias.<br>- Allocation concealment: 100% of the studies presented low risk, while<br>- 80% of the studies presented a low risk for the performance bias domain<br>- The evaluation of detection, attrition, and reporting bias showed that 100% of the studies presented low risk of bias.<br>- According to the analysis of other sources of bias (assessment of sun exposure and skin color), 60% of the studies presented low risk.”	
		Harm (se25(OH)D>250 nmol/L)	5 studies	most children reached or maintained sufficiency status with no concentrations of 25(OH)D > 250 nmol/L	Meta-analysis not conducted		

Black 2012	foods fortified with D2 or D3 vs. unfortified food or regular diet	Δ circulating 25(OH)D concentrations	16 independent studies (from 15 publications); n= 1513, 767 treated and 746 controls)	19.4 nmol/L (95% CI: 13.9, 24.9), corresponding to a 1.2 nmol/L (95% CI: 0.72, 1.68) increase in 25(OH)D for each 1μg/d ingested [mean serum 25(OH)D (nmol/L) = 1.198 (vitamin D intake) + 2.711; adjustedR^2= 0.67; P< 0.001]	"There was a high level of heterogeneity across the 16 studies P= < 0.0001; I^2 = 89%"<br><br>- latitude: ≤40° compared with lower latitude [22.4 (14.8, 30.0) and 17.3 (10.4, 24.3), respectively]. Heterogeneity remained high at 91% for both.<br>- Mean baseline 25(OH)D concentrations: <50 nmol/L compared with ≥50 nmol/L [24.9 (15.6, 34.1) and 13.6 (9.5, 17.7), respectively]. Heterogeneity was 94% among studies with a mean baseline 25(OH)D <50 nmol/L but was much lower at 35% among studies with a mean baseline 25(OH)D ≥50 nmol/L.<br>- dose: heterogeneity was 78% in those studies using ≥10μg/d and 73% in those studies using <10μg/d. The overall treatment effect ≥10μg/d: 25.9 (19.3, 32.4), <10μg/d [11.6 (6.7, 16.6)]."	5 studies scored <3 on the Jadad scale, the remainder achieved a score of =3	GRADE not assessed
Brett 2018	Food fortified with vitamin D vs unfortified food	Mean change in serum 25(OH)D per 100 IU vitamin D/d	7 trials	6.9 nmol/L [95% CI: 3.7 - 10.0 nmol/L]; I^2 = 99.9%	Heterogeneity: I^2 = 99.9%<br><br>"When baseline vitamin D status <50 nmol/L, fortified-food intervention groups (n = 4) showed increases in serum 25(OH)D of 4.2–10.8 nmol/L /100 IU vitamin D/day	- Cochrane tool: study designs were qualitatively high, 8–92% of studies had a low risk of bias for the 7 categories of bias<br>- 96% had Jadad scores ≥4	GRADE not reported
Brooker 2022	Fortified milk or formula with vit D vs non-fortified milk or formula"	serum 25(OH)D	3 trials	"Two studies reported significant increases in serum vitamin D concentrations during the intervention"<br><br>1 trial -" no difference in either vitamin D	Meta-analysis not conducted	thirteen articles were considered to have 'some concerns', four were deemed 'low risk', and two 'high risk'	GRADE not reported

				concentrations at the end of the intervention period"<br><br>1 study with a 12-month intervention period reported a change in the relevant outcomes at four months only.			
Cashman 2021	vitamin D intervention group vs control /placebo group	Daily Vitamin D intake needed	11 trials	12 µg/day of vitamin D, supplied by fortified foods together with habitual intake, can prevent wintertime vitamin D deficiency (serum 25(OH)D <30 nmol/L) in the vast majority of individuals<br><br>Cutoff: ≥ 50 nmol/L<br>The vitamin D intake estimate required to maintain 90% and 95% of individuals was 33.4 µg/day and 57.5 µg/day, respectively using the adjusted model, and 17.0 µg/day and 28.1 µg/day, respectively using the unadjusted model.<br><br>Cutoff: ≥ 25 nmol/L<br>vitamin D intake needed to maintain 97.5% of individuals above this threshold to be 5.9 µg/ day based on the unadjusted model; this decreased to 2.0 µg/day with adjustment for covariates	- age group: intake estimates allowing 90%, 95% and 97.5% of children (2–17.9 years) to maintain serum 25(OH)D ≥ 50 nmol/L were two to five times lower than those of adults (≥ 18 years)<br>- compliance: no statistical difference (P = 0.11) in median compliance between adults and children<br>- median requirement (i.e. EAR) at the 50 nmol/L serum 25(OH)D threshold was similar for adults and children (5.0 and 5.1 µg/day, respectively) based on the unadjusted model, but was higher for children than adults (3.8 versus 2.6 µg/day, respectively) based on the adjusted mode<br>- latitude ≥ 50 N (7 RCTs) results were broadly similar to the equivalent estimates from the full RCT dataset (i.e., 40–63 N)	All 11 studies achieved a Jadad score of ≥ 3 (18% and 55% with scores of 4 and 5, respectively)	GRADE not reported

				Cutoff: ≥ 30 nmol/L estimated a vitamin D intake of 12.2 µg/ day to maintain 97.5% of individuals ≥ 30 nmol/L using the unadjusted model and 4.5 µg/day using the adjusted model			
Cranney 2007	Food fortified with vitamin D vs unfortified food, usual diet or no intervention	25(OH)D	11 trials	7 trials were included in the meta-analysis. Combining all seven trials was not possible due to heterogeneity of the treatment effect (I2 = 79.2 percent).<br>The individual weighted mean differences (WMD) demonstrated a clear trend toward a significantly higher absolute change in serum 25(OH)D in the treatment group versus control.	• younger versus older individuals: younger individuals: significant absolute increase in 25(OH)D levels (4 trials, N = 323, WMD 17.02, 95% CI 12.49, 21.56, heterogeneity I^2 = 44.4 percent)<br>• "Combined data from 2 trials (N = 275) similar in the dietary vehicle used (fortified skim milk), population studied (postmenopausal women and young adults), dose of vitamin D (400 and 480 IU daily), type of vitamin D (D3), 25(OH)D assay (RIA), and outcome (total 25(OH)D) demonstrated a significantly higher absolute change in serum 25(OH)D (WMD 15.71, 95% CI 12.89, 18.53, heterogeneity I^2 = 0 percent) in the treatment group"	Six of 11 trials had Jadad quality scores ≥3	Not reported
		Harms	11 trials	None of the studies reported adverse side effects	Not applicable		Not reported
Cranney 2008	Food fortified with vitamin D vs control	25(OH)D concentration	11 food fortification RCTs	Treatment effect varied from 15 to 40 nmol/L.	Heterogeneity of the food fortification trials limited our ability to quantitatively synthesize the results.	- 52% of the studies > 3 or higher on the Jadad 5 scale, and	GRADE not reported

					Could not determine whether the effect of eating food fortified with vitamin D on serum 25(OH)D concentration varied by age, body mass index, or ethnicity.	- 29% of these had a score of 4.<br>- most of the higher-quality evidence came from studies of postmenopausal women and men >60 y;<br>- relatively few high-quality controlled studies in infants, children, and adolescents.	
		Adverse events		The food fortification trial reports did not list any adverse events.	Not applicable		GRADE not reported
Das 2013	Fortified food with vit D vs unfortified food with vit D	Serum 25 (OH)vitamin D3	Children<br>7 trials	"Analysis of the results from RCTs showed that fortification with vitamin D significantly increased serum concentration of 25-hydroxy-vitamin D3 (SMD: 1.23 (95% CI: 0.35, 2.11))"	Vitamin D alone:<br>- in children SMD: 1.76 (95% CI: 0.37, 3.15; 2 studies, 651 participants)<br><br>Combined vitamin D and Ca:<br>- in children: SMD: 1.58 (95% CI: 1.28, 1.87; 1 study, 235 participants)	Not reported	Quality of evidence: Moderate
		Serum 25(OH)vitamin D3	Women<br>13 trials	"Vitamin D and calcium fortification in WRA had a non-significant impact on vitamin D3 levels with a SMD of −1.10 (95% CI: -3.81, 1.60)"; "For the post-menopausal women, pooled analysis showed significant impacts on serum concentration of 25-hydroxy-D3 with a SMD of 0.82 (95% CI: 0.30, 1.34)"	In women of reproductive age:<br><br>- Vitamin D alone: SMD: 0.26 (95% CI: -0.22, 0.75; 1 study, 66 participants)<br><br>- Combined vitamin D and Ca: SMD: -2.50 (95% CI: -3.22, -1.78; 1 study; 55 participants)	Not reported	Quality of evidence: Moderate
		Serum PTH	Children 7 trials	"Analysis of the results from RCTs showed that fortification with vitamin D significantly (…) reduced	- Vitamin D only: no studies identified<br>- Ca and Vitamin D: 2 studies 327 participants, SMD: -0.52 (95% CI: -0.74, -0.29)	Not reported	Quality of evidence: Low

				serum parathyroid hormone concentration (SMD: -0.40 (95% CI: -0.56, -0.24))”			
		Serum PTH	Women 13 trials	“The analysis showed that vitamin D and calcium fortification in WRA had a non-significant impact on (…) serum parathyroid hormone levels with SMD of −0.01 (95% CI: -0.32, 0.30).” “For the post-menopausal women, pooled analysis showed significant impacts on (…) serum parathyroid hormone concentration with SMD of −2.53 (95% CI: -4.42, -0.65).”	- Vitamin D only: SMD: 0.10 (95% CI: -0.28, 0.48, 2 studies 108 participants)<br>- Ca and Vitamin D: SMD: -0.23 (95%CI: -0.78, 0.32, 1 study 52 participants)<br><br>- Post-menopausal women: no studies identified	Not reported	Quality of evidence: Low
		Serum Ca	Children 7 trials	SMD: -0.40 (95%CI: -0.59, -0.20)	- Vitamin D only: no studies identified<br>- Ca and Vitamin D: 1 study 235 participants, SMD: -0.50 (95%CI: -0.76, -0.24)	Not reported	Quality of evidence: Low
		P1NP	3 studies	SMD: −3.36 (95%CI: -6.37, -0.35)	Sub-group analysis not conducted	Not reported	Not reported
		CTx	4 studies	SMD: −4.93 (95%CI: -7.78, -2.08)	Sub-group analysis not conducted	Not reported	Not reported
Dunlop 2021	fortified food with vit D (one study biofortified with vit D) vs unfortified food	Serum 25(OH)D	34 trials (2398 adults: 1345 intervention, 1053 controls; 1532 children: 970 interventions, 562 controls)	Treatment effect (WMD) of 21.2 nmol/L (95% CI 16.2, 26.2)”	“Heterogeneity was high (I^2 = 96%, chi-squared P < 0.001”<br><br>**- by baseline circulating 25(OH)D**: greater effect size for studies with baseline <50 nmol/L (WMD 26.5 (20.8, 32.2), p<0.001, v = 95%) than ≥50 nmol/L (WMD 14.9 (8.62, 21.2), p<0.001, I^2 =94%)	- 32 were assigned a Jadad Scale score of ≥3<br>- 24 were assigned score of 5<br>- Two publications were assigned a score of 2	GRADE not reported

					- by **vitamin D treatment dose** ≥10 µg/d (WMD 26.2 (18.3, 34.2), p<0.001, I^2 =97%) than <10 µg/d. (WMD 14.6 (8.75, 20.5), p<0.001, I2=93%)<br><br>**- by effect of addition of calcium to treatment foods:** Calcium dose≥500 mg/d had a considerably lower overall treatment effect (WMD 10.4 (5.04, 15.7), I^2=80%) than Ca <500 mg/d (24.6 (18.4, 30.7), I^2 =97%)<br><br>**- effect of interventions by vitamin compound:** greater for vitamin D3 (25 studies) (effect size 26.8 nmol/L; 95% CI: 21.1, 32.5; I^2 = 97%; chi-squared P < 0.001) than for vitamin D2 (4 studies) (effect size 17.2 nmol/L; 95% CI: 2.78, 31.7; I^2 = 96%; chi-squared P < 0.001). However, heterogeneity was high<br><br>***- health and diabetes***: healthy participants (n=19): effect size was similar to the overall effect size (21.5 (13.6, 29.4), I^2 =98%, P<0.001). Participants with type 2 diabetes (n=3): Effect size was 34.2 nmol/L (95% CI: 28.1, 40.3) with low heterogeneity (I^2 = 0%; chi-squared P = 0.566).<br><br>**- multivariate metaregression: overall effect of interventions.**<br>Low vitamin D status (effect difference 9.38 nmol/L; 95% CI: 0.17, 18.6), daily vitamin D dose ≥10 µg (effect difference 12.0 nmol/L; 95% CI: 2.40, 21.6), and difference in daily calcium dose ≥500 mg (effect difference –11.9 nmol/L; 95 CI: –23.6, –0.30) between intervention and control groups were		

					significantly associated with the effect of the interventions.<br><br>**Dose-response analyses**<br>"Wald test for nonlinearity was P < 0.001 for dose-response analyses conducted using restricted cubic splines for all studies combined, adults, children, and for vitamin D3 only. Thresholds occurred at ~25.6 nmol/L at a dose of ~21 μg/d for all studies combined and at ~29.9 nmol/L at a dose of ~20 μg/d for vitamin D3. When studies were separated by adults and children, the rate began to slow at a mean difference of ~18 nmol/L 25(OH)D, but at a higher dose (~10 μg/d) in adults than children (~5 μg/d)."		
Emadzadeh 2022	Fortified food with vitamin D vs unfortified food (or a regular diet) for at least one month	serum vitamin D	48 interventional groups	difference in means=16.518 nmol/L, P-value<0.001, CI 95%: 11.618 to 21.418	- duration of intervention: no significant differences ≤ 6 months: (MD: 18.074 P-value<0.001, CI 95% 12.23 to 23.918), > 6 months (MD: 10.938, P-value=0.002, CI 95% 4.009 to 17.867).<br>- age: >18 yrs old (difference in means=19.453, P-value<0.001, CI 95%: 12.859 to 26.048), <18 years (difference in means=8.686, P-value=0.064, CI 95%: −0.497 to 17.869).<br>- Meta-regression did not show statistically significant associations between mean difference of vitamin D level and latitude of where the study was undertaken (P=0.37)	- 13 studies obtained a complete Jadad score (complete 5 scores).<br>- The quality was inadequate (<3) in 8 studies, while others had a score of three or more	GRADE not reported
		serum PTH	25 reports	difference in means=−5.148, P-value<0.001, CI 95%: −7.341 to −2.955	- Age: similar in both >18 and <18-year-old participants (−4.181, p<0.001, CI 95%: -6.503 to 1.859) vs. (−8.262, p=0.002, CI95%: -13.497 to −3.02) respectively		GRADE not reported

		serum IGF-1	8 interventional groups	difference in means=42.789, P-value=0.003, CI 95%: 14.607 to 70.971	Sub-group analysis not conducted		GRADE not reported
		serum telopeptides of type-1 collagen (C-terminal: CTx)	8 reports	difference in means=−0.027, P-value=0.018, CI 95%: −0.05 to −0.005	Sub-group analysis not conducted		GRADE not reported
		serum osteocalcin	11 study	difference in means=0.803, P-value=0.279, CI 95%: −0.65 to 2.255	- age: <18 yrs: (MD: 3.886, P-value=0.28, CI95%: -3.165 to 10.936;) >=18 yrs: (MD: 0.381, P-value=0.614, CI95%: -1.099 to 1.861;).<br>- duration of intervention: >6 months (2 studies), did not indicate different results vs studies <6 months of interventions		GRADE not reported
		Bone Mass Density (BMD) - total, femoral neck, and lumbar BMD		- vitamin D fortification was not associated with a significant increase in BMD in each specific anatomical site - except spine: difference in means=0.081, P-value<0.001, CI 95%: 0.047 to 0.116	Sub-group analysis not conducted		GRADE not reported
Emadzadeh 2020	Fortified food with vit D vs placebo	Weight	15 trials	difference in means, − 0.065; 95% CI, − 0.439 to 0.309; P = 0.734) (I2: 88.5%, P < 0.001	- duration of intervention: ≤6 months (difference in means, − 0.368; 95% CI, − 0.818 to 0.081; P = 0.109). >6 months (difference in means, 0.904; 95% CI, 0.119 to 1.688; P = 0.024)<br><br>- the type of fortification": calcium plus vitamin D fortified foods (difference in means, 0.374; 95% CI, − 0.221 to 0.97; P = 0.218). Vitamin D fortification (difference in means, − 0.560; 95% CI, − 1.130 to 0.010; P = 0.054).	the study quality was not appropriate in all those studies that were included	GRADE not reported
		Body mass index	16 trials	difference in means, − 0.044; 95% CI, − 0.229 to	duration of the intervention: <6 months (difference in means, − 0.156; 95% CI, −		GRADE not reported

				0.142; P = 0.643) (I2: 83.36%, P < 0.001)	0.362 to 0.05; P = 0.138), >6 months (difference in means, 0.382; 95% CI, 0.195 to 0.570; P < 0.001)"		
		Fat mass	10 studies	difference in means, − 0.542; 95% CI, − 1.207 to 0.123; P = 0.11) (I2: 85.05%, P < 0.001)	- duration of the intervention: ≤6 months, (difference in means, − 0.677; 95% CI, − 1.408 to 0.054; P = 0.07). Two studies >6 months (difference in means, 0.071; 95% CI, − 2.221 to 2.363; P = 0.952).<br><br>- fortification type: calcium plus vitamin D (difference in means, − 0.051; 95% CI, − 1.118 to 1.016; P = 0.926), vitamin D fortified food (difference in means, − 0.888; 95% CI, − 1.771 to − 0.004; P = 0.049)		GRADE not reported
		lean mass	3 trials	difference in means, − 0.089; 95% CI, − 0.496 to 0.317; P = 0.666) (I2: 40.65%, P = 0.16)	Sub-group analysis not conducted		GRADE not reported
		Waist circumference	6 studies	difference in means, − 1.283; 95% CI, − 1.892 to − 0.674; P < 0.001) (I2: 13.24%, P = 0.33	"Fortification type" subgroup: calcium plus vitamin D fortified foods (difference in means, − 0.863; 95% CI, − 1.959 to 0.233; P = 0.123). Vitamin D fortified foods (5 studies - difference in means, − 1.448; 95% CI, − 2.242 to − 0.654; P < 0.001)		GRADE not reported
		Hip circumference	3 trials	difference in means, − 0.127; 95% CI, − 0.842 to 0.589; P = 0.729) (I2: 0%, P = 0.88)	Sub-group analysis not conducted		GRADE not reported
		Waist-to-hip ratio	5 trials	difference in means, − 0.020; 95% CI, − 0.035 to − 0.004; P = 0.012) (I2: 82.19%, P < 0.001	Sub-group analyses not conducted		GRADE not reported
Emadzadeh 2020	Fortified food with vitamin d vs Regular diet (unfortified with vit d)	Fasting serum glucose	11 trials	MD: −2.772, p = .041, and 95% CI: −5.435 to −0.109	- Type of intervention: Vitamin D alone: (−3.641, 95% CI: −7.229 to −0.053) significantly reduced FSG more than Ca–D group (−1.8, 95% CI: −7.344 to 3.741)	All studies were with high scores (>3) according to Jadad scale	GRADE not reported

					- duration of intervention: in <3 months of intervention: MD equals to −7.677 [95% CI: −14.067 to −1.296]; in >3 months of intervention: MD equals to −1.554 [95% CI: −2.393 to −0.716]		
		Fasting serum insulin	9 trials	MD: −2.937, P = .001 and 95% CI: −4.695 to −1.178	- type of intervention: pooled effect in Ca–D subgroup: −2.122, 95% CI: −5.865 to 1.61; in Vitamin D subgroup: −3.432, 95% CI: −5.83 to −1.035<br><br>- intervention duration: short versus long duration (MD: −3.589, 95% CI: −5.9 to −1.278 vs. MD: −0.444, 95% CI: −2.284 to 1.396, respectively		GRADE not reported
		HOMA-IR, homeostatic model assessment of insulin resistance	5 trials	MD: −1.608, p = .039 and 95% CI: −3.138 to −0.079	Sub-group analysis not conducted		GRADE not reported
		hemoglobin A1C	7 trials	MD: 0.034, p = .113 and 95% CI: −0.655 to 0.069	Sub-group analysis not conducted		GRADE not reported
Fonseca Santos 2022	Intake of food fortified with vitamin D and calcium vs. nonfortified food	25(OH)D	5 trials	25(OH)D:<br>- in 2 studies (fortifying cheese): significant increase<br>- fortified yogurt (Higher doses of vitamin D3 (10 ug) and calcium): significant increases<br>- In one study (cheese with Vitamin D and Ca) no significant increases	Meta-analysis not conducted	- 2 studies had low risks of bias for all the items evaluated<br>- Other studies had mixed low risks, uncertain risks, and high risks of bias.	GRADE not reported
		PTH	5 trials	- decrease in PTH secretion (with baseline PTH values > 60 ng/mL) was associated with the increase in the	Meta-analysis not conducted		GRADE not reported

				25(OH)D concentrations (dose dependent results)			
		CTX	5 trials	- 4 trials: reduction of markers of bone resorption<br>- 1 study: No changes	Meta-analysis not conducted		GRADE not reported
		TRAP5b	5 trials	- 4 trials: reduction of markers of bone resorption<br>- 1 study: No changes	Meta-analysis not conducted		GRADE not reported
		P1NP	4 trials	- 1 study: significant increase (soft plain cheese with vitamin D + Ca)<br>- Other studies: no significant alterations	Meta-analysis not conducted		GRADE not reported
Gasparri 2019	Vitamin D fortified yogurt (with or without calcium) vs Plain yogurt ((with or without calcium))	serum 25-hydroxy vitamin D (nmol/L	9 RCTs, n=665	MD = +31.00 nmol/L [95% CI: 26.10 to 35.91; P < 0.00001; I^2=100%]	"Considerable between-study heterogeneity was observed for most outcomes"<br>Sub-group analysis not conducted	all studies were low/ unclear risk of bias for the Random sequence generation/ Allocation concealment/ Participant personnel blinding/ Outcome assessment blinding/ Incomplete outcome data/ Selective reporting/ Other bias	Not reported
		parathyroid hormone (ng/L)	4 RCTs, n=265	MD – 15.47 ng/L [-19.97 to -10.96; P < 0.00001; I^2=93%]	Sub-group analysis not conducted		Not reported
		body weight (kg)	7 RCTs, n=589	MD -0.92 kg [-1.44 to -0-40; P=0.0005; I^2=99%]	Sub-group analysis not conducted		Not reported
		BMI (kg/m^2)	6 RCTs, n=486	MD -0.15 kg/m^2 [-0.33 to 0.03; P=0.09; I^2=2%]	Sub-group analysis not conducted		Not reported
		waist circumference (cm)	5RCTs, n=426	MD –2.01 cm [-2.56 to –1.47; P < 0.00001; I^2=80%]	Sub-group analysis not conducted		Not reported
		fat mass (percentage	6 RCTs, n=486	MD -1.3 percentage [-2.95 to 0.35; P=0.12; I^2=94%]	Sub-group analysis not conducted		Not reported
		total cholesterol (mg/dL)	5 RCTs, n=469	MD -13.38mg/dL [-20.19 to –6.56; P=0.0001; I^2=98%]	Sub-group analysis not conducted		Not reported

		triglycerides (mg/dL)	5 RCTs, n=469	MD –30.12 mg/dL [-43.22 to –17.12; P < 0.00001; I^2=95%]	Sub-group analysis not conducted		Not reported
		LDL cholesterol (mg/dL)	5 RCTs, n=469	MD –7.86 mg/dL [-15.35 to –0.37; P=0.04; I^2=99%]	Sub-group analysis not conducted		Not reported
		HDL cholesterol (mg/dL)	5 RCTs, n=469	MD 1.48 mg/dL [-0.18 to 3.13; P=0.08; I^2=96%]	Sub-group analysis not conducted		Not reported
		homeostatic model assessment insulin resistance (HOMA-IR)	4 RCTs; n= 369	MD –2.18 [-2.92 to –1.44; P < 0.00001; I^2=68%]	Sub-group analysis not conducted		Not reported
		fasting serum glucose (mg/dL)	4 RCTs, n=339	MD –22.54 mg/dL [-37.55 to –7.52; P=0.003; I^2=99%]	Sub-group analysis not conducted		Not reported
		systolic blood pressure (mmHg)	4RCTs, n=366	MD –0.97 mmHg [-3.02 to 1.08; P=0.35; I^2=89%]	Sub-group analysis not conducted		Not reported
		diastolic blood pressure (mmHg)	4 RCTs, n=366	-0.29 mmHg [-2.13 to 1.55; P=0.76; I^2=39%]	Sub-group analysis not conducted		Not reported
Lam 2016	Vitamin D and calcium-fortified cheese, yogurt, and buns. Comparator not specified or uncontrolled	25(OH)D,	2 RCTs (n=80) + 2 pretest/ posttest 1-group design	"All studies reported an increase in 25(OH)D,"	Meta-analysis not conducted	Not reported	GRADE not reported
		PTH	4 studies	Consistently decrease with treatment, suggesting that	Meta-analysis not conducted	Not reported	GRADE not reported

				a minimum of 400 IU vitamin D and 302 mg calcium for 1 month is needed to see this effect.			
Niedermaier 2021	Fortified food with vitamin D vs placebo	serum levels of 25(OH)D	10 study	increases by 10–48 nmol/L<br><br>approximately 1.2 nmol/L per µg (40 IU) of vitamin D	Meta-analysis not conducted	Not reported	Not reported
		Cancer mortality	3 studies	Fortification with 400 IU, 800 IU, and 2000 IU vit D /day found cancer mortality reductions by 11%, 15%, and 17%, respectively	Meta-analysis not conducted	Not reported	Not reported
Nikooyeh 2018	fortified food either with vitamin D2 or D3 (with or without calcium) versus control	serum 25(OH)D concentrations	5 RCTs	MD 34.68 [28.59 - 40.77]; I^2=55%	Sub-group analysis not conducted	All studies scored ≥3 on the Jadad scale.<br>All studies at low risk of bias using Cochrane tools.	Not reported
Nikooyeh 2022a	vitamin D-fortified food vs. the same, but unfortified food	serum 25(OH)D concentrations	11 RCTs	MD 20.29 [13.32 - 27.25]; I^2=97.61%	• Vitamin D fortified foods caused significant increases in all age subgroups ((under 5 years: MD 19.3, 95% CI 4.7–33.9; 5–12 years: MD 22.8, 95% CI 13.0–32.6; 12–18 years: MD 4.2, 95% CI 1.3, 7.0, P < 0.001); (1 study was conducted in adolescents with small amount of vitamin D as a fortificant (80 IU/day)).<br>• Treatment effect was better if the mean serum 25(OH)D <50 nmol/L at baseline (MD 24.6, 95% CI 12.8–36.4 vs MD 15.1, 95% CI 6.3–23.9); however, no differing patterns were clearly evident between these subgroups (chi2 = 1.58, P = 0.21).	- In 14 trials, the information on the methods used for allocation concealment was unclear<br>- All trials provided data on losses to follow-up and random sequence generation.<br>- 8 trials reported losses of >10%.<br>- 3 trials reported no blinding	Not reported

						methods for participants and the research team	
Nikooyeh 2022b	foods fortified with vitamin D2 or vitamin D3 alone or in combination with Ca vs. unfortified foods or no intervention	serum 25(OH)D concentrations	23 RCTs	MD: 25.4 nmol/l, (95 % CI 19.5, 31.3; 2002 participants; I2=97.15%)	• Dose: statistically significant effect in the subgroups of all studies with all doses used for fortification (both more than and less than 1000 IU/d). The effect was significantly stronger >1000 IU vitamin D /day (>1000 IU, MD: 41·5 nmol/l, (95 % CI 33·0, 50·0) v. < 1000 IU, MD: 18·2, (95 % CI 12·7, 23·7), P < 0·001)"<br>• Fortificant: effects in all sub-groups (vitamin D2 (n=3): 27.93 [19.39 - 36.46]; vitamin D3 (n=29): 25.26 [18.75 - 31.76]<br>• Duration: effects in all subgroups (less than 3 months (n=11): 28.09 [12.50 - 43.67]; 3 months and more (n=21): 24.27 [19.17 - 29.37)<br>• Vehicle: effects in all sub-groups (dairy products (n=19): 21.25 [12.51 - 29.98]; Juice (n=5): 34.40 31.46 - 37.33; Grain products (n=5): 31.72 [18.42 - 45.01]; Oil (n=1): 40.50 [30.65 - 50.35; Dairy and grain products (n=2): 25.66 [18.32 - 33.00]<br>• Sex: effects in all sub-groups (both genders (n=11): 38.20 [25.01 - 51.39; women (n=19): 19.30 [14.46 - 24.14]; men (n=2): 21.54 [12.00 - 31.07]	- All trials provided data on losses to follow-up; only four reported losses of >10 % (36,37,44,47).<br>- In eight trials, the information on the methods used for allocation concealment was unclear<br>- Two trials reported small sample size (< 15 subjects in each arm)	Not reported

					• Latitude: lower than 35 (n=7): 33.98 [25.05 - 42.90]; higher than 35 (n=25): 22.90 [16.07 - 29.90]		
O'Donnell 2008	Fortified food vs usual diet/ no intervention/ unfortified food	serum 25(OH)D concentrations<br><br>Weighted mean difference (WMD) (and 95% CIs)	7 trials (n=585)	in 4 studies, the serum 25(OH)D concentration was >80 nmol/L<br>the increment in the 25(OH)D concentration appears to be larger than the increment of 1 nmol/L/ug<br><br>Overall treatment estimate was not feasible because of significant heterogeneity (I 2 = 70.6%).<br><br>the individual WMDs showed a significantly greater increase in absolute mean change in serum 25(OH)D in the treatment group than in the control group: range 14.5 (95% CIs: 10.6, 18.4) nmol/L to 34.5 (17.64, 51.36) nmol/L (3.4 –25 ug vitamin D/d).	- Subgroup analysis by population or intake level did not explain the heterogeneity with respect to the treatment effect.<br><br>- 4 trials (n = 446; 3.45–20 ug vitamin D3/d) that used an RIA to measure serum 25(OH)D reduced heterogeneity of the treatment effect and showed a statistically significant increase in serum 25(OH)D [WMD = 15.70 (95% CI: 12.62, 18.77) nmol/L; I^2 = 0.0%; P = 0.77].<br><br>- vehicle: milk (4 trials, n = 466; 3.45–20 ug vitamin D/d) [15.63 (12.79, 18.48) nmol/L; I2 = 0.0%; P = 0.77]	- 5 trials had a total score of 3 on the Jadad scale.<br>- 8 trials provided data on losses to follow-up, 1 reported loss of >20%.<br>- In all trials, the information on the methods used for allocation concealment was unclear.<br>- 8 did not report whether they had included an intention-to-treat analysis; 1 reported performing a similar analysis	GRADE not reported
	Vitamin D–fortified cheese (15 ug vitamin D3/d) vs unfortified cheese or no cheese	Serum 25(OH)D	1 RCT	after 2-months: the serum 25(OH)D concentration decreased by a mean of 6 +/- 2 nmol/L (P = 0.001)	Sub-group analysis not conducted		GRADE not reported

	Fortified food vs usual diet/ no intervention/ unfortified food	Se PTH	6 trials	3 trials: significantly lower concentration at the end of the study in the intervention group than in the control group	Sub-group analysis not conducted		GRADE not reported
	Fortified food vs usual diet/ no intervention/ unfortified food	baseline 25-hydroxyvitamin D concentrations		Participants with lower baseline 25(OH)D concentrations (i.e., <50 nmol/L) were more likely to reach the higher end of study serum 25(OH)D concentrations.	Sub-group analysis not conducted		GRADE not reported
	Fortified food vs usual diet/ no intervention/ unfortified food	Harm	3 trials (n=434, n=204 intervention group)	8 participants dropped out/withdrew from the intervention group because of gastrointestinal side effects	Sub-group analysis not conducted		GRADE not reported
O'Mahony 2011	Fortified milk vs usual diet	Change from baseline of serum 25(OH)D (%)	2 trials (n=367)	In both studies, 25(OH)D concentrations increased for the supplemented group compared to baseline: 25.0% vs. 7.4% (control group: 4.1% vs – 19.9%)	Meta-analysis not conducted	Not reported	GRADE not reported
	Fortified Yogurt +/- Ca++ vs plain yogurt	Change from baseline of serum 25(OH)D (%)	1 trial (n=90)	daily intake of 25 µg of vitamin D3 with or without calcium significantly increased serum 25(OH)D3 concentrations after 12 weeks by 75.0% and 67.6%, (control: -10.6%)	Meta-analysis not conducted		GRADE not reported
		Glycemic status		glycemic status in diabetic patients was improved	Meta-analysis not conducted		GRADE not reported

	Fortification of cheese vs placebo/no cheese	Change from baseline of serum 25(OH)D (%)	2 trials (n=190)	1 study: 120% increase in serum 25(OH)D in the intervention group vs placebo cheese: -7.8%<br>1 study: Intervention group: -8.7% decrease in their serum 25(OH)D. (Control group with placebo or no cheese: 10% and 5,6%). The authors explanation: a higher baseline value of 25(OH)D in the supplemented group.	Meta-analysis not conducted		GRADE not reported
	Fortified orange juice vs placebo	Change from baseline of serum 25(OH)D (%)	2 trials (n=135)	positive effect on serum 25(OH)D concentration (+150%, 67.1% - with vitamin D2, 71.5% - with vitamin d3) vs placebo: 45% and –8.6%	Meta-analysis not conducted		GRADE not reported
	UV enhanced mushrooms in a soup vs placebo	Change from baseline of se 25(OH)D (%)	1 trial (n=27)	50% increase in serum 25(OH)D concentration (Ctrl: -28.9%)	Meta-analysis not conducted		GRADE not reported
	Fortified bread vs regular bread	Change from baseline of serum 25(OH)D (%)	1 trial (n=41)	serum 25(OH)D increased by approximately 60% (Ctrl:<br>-1.2%	Meta-analysis not conducted		GRADE not reported
		Serum PTH,	6 trials	2 studies: decrease in se PTH, 4 studies: No changes in se PTH.	Meta-analysis not conducted		
		Serum Ca	4 trials	- In 3 trials the Ca concentration remained stable<br>- 1 study: calcium concentration decreased in	Meta-analysis not conducted		

				the fortified rye bread group			
Soto-Mendez 2019	Milk and dairy products fortified with vitamin D vs. no exposure or unfortified products or products with a regular vitamin D content	Total cholesterol	2 studies (262 participants)	Meta-analysis was not possible (few available publications)<br><br>- 1 study: decrease (0.55 ± 0.97 compared with −0.60 ± 0.98 mmol/L, P = 0.04)<br>- 1 study no changes	Meta-analysis not conducted	- 1 study: low risk of bias,<br>- 1 study: unclear risk of bias for allocation concealment, random sequence generation, blinding of outcome assessment	GRADE not reported
		HDL cholesterol	1 study	No changes	Meta-analysis not conducted		GRADE not reported
		LDL cholesterol	1 study	decreased (0.21 ± 0.58 compared with −15.3 ± 21.7 mmol/L; P = 0.05)	Meta-analysis not conducted		GRADE not reported
		ratio of TC/HDL cholesterol	1 study	0.4 ± 0.6 compared with −0.3 ± 0.3; P = 0.02	Meta-analysis not conducted		GRADE not reported
		Triglyceride	2 studies	- 1 study decrease (mean ± SD 0.45 ± 0.48 compared with −0.27 ± 0.49 mmol/L; P = 0.02),<br>- 1 study: No changes	Meta-analysis not conducted		GRADE not reported
		Blood pressure	1 study	reductions in systolic (P = 0.017) and diastolic (P = 0.010) blood pressure	Meta-analysis not conducted		GRADE not reported
		glucose	1 study	No changes	Meta-analysis not conducted		GRADE not reported
Souza 2022	Bread fortified with vitaminD2/D3. Comparator not specified	serum 25(OH)D levels	10 studies	No meta-analysis was conducted<br><br>9 papers used vitamin D3 for bread fortification purposes and showed an effective response to	Meta-analysis not conducted	Not reported	GRADE not reported

				increasing 25(OH)D serum status."<br>Vitamin D3 is preferable to vitamin D2 from UVB irradiated yeast for bread fortification."			
		PTH		No meta-analysis was conducted<br><br>"3 studies did not find significant results in the serum PTH levels, 2 studies showed an increase, 4 studies showed a decrease in this parameter.	Meta-analysis not conducted	Not reported	GRADE not reported
		Serum calcium		"The bread fortification strategy did not affect the serum calcium levels."	Meta-analysis not conducted	Not reported	GRADE not reported
		se. OC, ALP, P1NP, CTX	1 study	no differences in se. OC, ALP, P1NP, CTX after 12 weeks of 30 µg of vitamin D3/day from four foods (150 g yoghurt, 60 g cheese, 1 egg and 9 g bread).	Meta-analysis not conducted	Not reported	GRADE not reported
Tangestani 2020	food fortification vs placebo	serum 25(OH) D	14 trials	Se 25(OH)D: MD=16.94 nmol/L, 95% CI: 13.38, 20.50; p<0.001; I2=99.0%, p<0.001	25(OH) D increased more in those studies<br>- population (patients) (I^2 = 99.0%; p=0.000),<br>- fortification dose of vitamin D >4000 IU/day (I 2=99.0%; p < 0.001),<br>- and trial duration >3 months and <6 months (I 2=99.0%; p < 0.001)	- 6 studies Jadad score =5<br>- 2 studies Jadad score = 4<br>- 5 studies Jadad score = 3<br>- 2 studies Jadad score = 2	Grade not reported

		hip and spine BMD	6 trials (15 interventional groups)	MD: 0.03 gr/cm2, 95% CI: (0.02, 0.05); $p < 0.001$, and I2=58.8%; $p < 0.002$	- VDFs interventions improved BMD in studies conducted on both sex (I^2 = 58.8%; p=0.002),<br>- age of < 35 (I 2 = 58.8%; p=0.002), - vitamin D dose of higher than 400 IU/d (I^2 = 58.8%; p=0.002) and<br>- Ca dose of higher than 1000 mg/d (I^2 =58.8%; p=0.002).<br>- Vitamin D fortification via non-dairy food (I^2 =58.8%; p=0.002)<br>- more effective at spine site area (I^2 =58.8%; p=0.002) and<br>- in low quality studies (I^2 =58.8%; p=0.002)."	- 5 studies Jadad score = 1	
		Serum PTH	15 studies	MD: -9.22 ug/L; 95% CI: (-1 4.97, -3.46); p= 0.002, heterogeneity (I 2= 98.8%; $p < 0.001$).	Potential source of heterogeneity dose of vitamin D fortification.<br><br>Serum PTH had more reduction in dose of >4000 IU/day of vitamin D (I^2 =58.8%; p=0.002) compared with doses of<400, 400–1000, and 1000–4000"		
		serum OC	7 trials (9 intervention)	MD: 4.097 ug/L; 95% CI: (-7.20, 15.39); p = 0.477	sources of heterogeneity: age, study population, fortification dose of vitamin D, and trial duration.<br><br>- VDFs showed greater increase of serum OC in trials in which participants had age of > 35 years (I^2 = 99.8%; $p < 0.001$) compared with age of <35,<br>- patient population (I^2 = 99.8%; $p < 0.001$) compared with apparently healthy subjects,<br>- fortification dose of >400 IU/d of vitamin D (I^2 = 99.8%; $p < 0.001$) compared with dose of <400 and<br>- trial duration of >6 months (I^2 = 99.8%; $p < 0.001$) compared with <3, and 3–6		

		Serum ALP	8 studies (10 intervention)	MD: -3.434 ug/L; 95% CI: (-7.959, 1.090); p = 0.137, I 2 = 97.1%; p = 0.2.	Probable sources of heterogeneity: trial duration and quality of studies.<br><br>- ALP was reduced in studies with duration of >3 months (I^2 =97.1%; p < 0.001) compared with duration of <3 months,<br>- and in trials judged of lower quality (<2) (I^2 =97.1%; p < 0.001) than those judged of higher (>3) quality.		
		serum CTX	6 studies (4 of them were reported serum CTX in mg/L and 2 in nmol/L)	studies with different unites were analyzed separately (not comparable).	Pooled estimated of MDs for 4 studies in mg/L: -0.060 (95% CI: (-0.15, 0.03); p ¼= 0.218) and (I 2 = 96.5%; p < 0.001)<br><br>for 2 other studies in nmol/L was -0.307 (95% CI: (-1.07, 0.46); p = 0.43) and (I 2 = 0.0%; p = 0.86).		
		serum P1NP	3 studies	Fixed model: MD: -1.13 ng/ml; 95% CI: (-13.76,11.48); p = 0.86 and (I 2 = 37.3%; p = 0.2) random model: MD:1.34; 95% CI: (-17.55, 20.23); p = 0.88 and (I 2 = 37.3%; p = 0.2).	Sub-group analysis not conducted		
Whiting 2015	Fortified yogurt vs placebo	Mean rate constant (change in 25(OH)D in nmol/L per µg vitamin D intake)	1 study (n=89)	Net rise in 25(OH)D: 20.2 nmol/L<br><br>Rate Constant: 2.02 nmol/L /ug<br><br>Administering 10 ug Vitamin D/ day: serum 25(OH)D status rose from “insufficient” (30–50 nmol/L) to “sufficient” (>50 nmol/L)	Meta-analysis not conducted	Not reported	GRADE not reported
