# Supplementary material for: Fortification of Staple Foods for Household Use with Vitamin D: An Overview of Systematic Reviews
Source: Nutrients. 2023 Aug 26;15(17):3742. doi: 10.3390/nu15173742 (PMC10489979; doi:10.3390/nu15173742)
Supplement: Supplementary file 1 [file nutrients-15-03742-s001.zip › Supplementary_File_S4_Excluded_studies.pdf]

Author	Title	Reason for exclusion
**Abrams et al., 2003**(1)	Calcium, magnesium, phosphorus and vitamin D fortification of complementary foods	Wrong study design (narrative review)
**Al Khalifah et al., 2018** (2)	The impact of vitamin D fortification of staple food for children: A systematic review and meta-analysis	Publication withdrawn
**Andersen et al., 2001** (3)	Towards a strategy for optimal vitamin D fortification (OPTIFORD)	Wrong study design (narrative review)
**Balachandar et al., 2021**(4)	Relative Efficacy of Vitamin D2 and Vitamin D3 in Improving Vitamin D Status: Systematic Review and Meta-Analysis	Wrong intervention (no staple food fortification)
**Barbagallo et al., 2022** (5)	Effect of Calcifediol on Physical Performance and Muscle Strength Parameters: A Systematic Review and Meta-Analysis	Wrong intervention (no staple food fortification)
**Beaudart et al., 2014** (6)	The effects of vitamin d on skeletal muscle strength, muscle mass and muscle power: A meta-analysis of randomized controlled trials	Wrong intervention (supplementation)
**Bendik et al., 2014** (7)	Vitamin D: a critical and essential micronutrient for human health	Wrong study design (narrative review)
**Bhutta et al., 2015** (8)	Food fortification as a strategy for alleviating micronutrient deficiencies in low-and middle-income countries: A systematic review	Wrong intervention (fortification not with vitamin D)
**Bischoff-Ferrari et al., 2019** (9)	Vitamin D Supplementation in Older Adults: Is the Hype Definitely Over?	Wrong intervention (supplementation)
**Bjelakovic et al., 2011** (10)	Vitamin D supplementation for prevention of mortality in adults	Wrong intervention (supplementation)
**Bolland et al., 2014** (11)	Calcium and vitamin D: An update	Wrong study design (narrative review)
**Bonjour et al., 2009** (12)	Minerals and vitamins in bone health: the potential value of dietary enhancement	Wrong study design (narrative review)
**Brandi et al., 2013** (13)	Calcidiol [25(OH)D3]: from diagnostic marker to therapeutical agent	Wrong study design (narrative review)
**Brenner et al., 2022** (14)	Potential of vitamin d supplementation or vitamin d food fortification for preventing cancer deaths in germany	Publication withdrawn
**Caini et al., 2021**(15)	Vitamin d and the risk of non-melanoma skin cancer: A systematic literature review and meta-analysis on behalf of the italian melanoma intergroup	Wrong intervention (no staple food fortification)
**Calvo et al., 2005**(16)	Vitamin D intake: a global perspective of current status	Wrong study design (narrative review)
**Calvo et al., 2004** (17)	Vitamin D fortification in the United States and Canada: current status and data needs	Wrong study design (narrative review)

**Cashman et al., 2019** (18)	Individual participant data (IPD)-level meta-analysis of randomised controlled trials among dark-skinned populations to estimate the dietary requirement for vitamin D	Systematic review protocol
**Cashman et al., 2016** (19)	Tackling inadequate vitamin D intakes within the population: fortification of dairy products with vitamin D may not be enough	Wrong study design (narrative review)
**Cashman et al., 2022** (20)	Individual participant data (IPD)-level meta-analysis of randomised controlled trials to estimate the vitamin D dietary requirements in dark-skinned individuals resident at high latitude	Wrong reporting (results of fortification and supplementation studies were analysed together)
**Chibuzor et al., 2020** (21)	Vitamin D, calcium or a combination of vitamin D and calcium for the treatment of nutritional rickets in children	Wrong study population (people with a specific disease)
**Chowdhury et al., 2014** (22)	Vitamin D and risk of cause specific death: Systematic review and meta-analysis of observational cohort and randomised intervention studies	Wrong intervention (no staple food fortification)
**Chua et al., 2023** (23)	The effect of vitamin D as adjunct to allergen immunotherapy (AIT): A systematic review and meta-analysis	Wrong intervention (no staple food fortification)
**Curtis et al., 1990** (24)	Infant nutrient supplementation	Wrong study design (narrative review)
**da Silva et al., 2022** (25)	Impact of vitamin D on cognitive functions in healthy individuals: A systematic review in randomized controlled clinical trials	Wrong intervention (supplementation)
**Darnton-Hill et al., 2019** (26)	Public Health Aspects in the Prevention and Control of Vitamin Deficiencies	Wrong study design (scoping review)
**Darnton-Hill et al., 2002**(27)	Fortification strategies to meet micronutrient needs: successes and failures	Wrong study design (narrative review)
**Das et al., 2018** (28)	Food fortification with calcium and vitamin D: Impact on health outcomes	Publication withdrawn
**Das et al., 2019** (29)	Food fortification with multiple micronutrients: impact on health outcomes in general population	Wrong intervention (fortification not with vitamin D)
**Datta et al., 2016** (30)	Food Fortification and Supplement Use-Are There Health Implications?	Wrong study design (narrative review)
**De-Regil et al., 2012** (31)	Vitamin D supplementation for women during pregnancy	Wrong intervention (supplementation)
**Dewey et al., 2020** (32)	Vitamin D from Supplements Consumed during Infancy and Toddlerhood and Bone Health: A Systematic Review	Wrong intervention (supplementation)<br><br>Duplicate

**Dötsch-Klerk et al., 2022** (33)	Modelling health and economic impact of nutrition interventions: a systematic review	Wrong intervention (no staple food fortification)
**Dunlop et al., 2020** (34)	Efficacy of vitamin D food fortification and biofortification in children and adults: a systematic review protocol	Systematic review protocol
**Eichler et al., 2012** (35)	Effects of micronutrient fortified milk and cereal food for infants and children: a systematic review	Wrong intervention (fortification not with vitamin D)
**El-Abbadi et al., 2014** (36)	Yogurt: role in healthy and active aging	Wrong study design (narrative review)
**Feehan et al., 2022** (37)	Vitamin D deficiency in nursing home residents: a systematic review	Wrong intervention (supplementation)
**Fraile Navarro et al., 2021** (38)	Vitamin D recommendations in clinical guidelines: A systematic review, quality evaluation and analysis of potential predictors	Wrong study design (systematic review of clinical guidelines)
**G et al., 2014** (39)	Fortification of foods with vitamin D in India	Wrong study design (narrative review)
**Grant et al., 2019** (40)	A Review of the Potential Benefits of Increasing Vitamin D Status in Mongolian Adults through Food Fortification and Vitamin D Supplementation	Wrong study design (narrative review)
**Grant et al., 2009** (41)	Estimated benefit of increased vitamin D status in reducing the economic burden of disease in western Europe	Wrong study design (narrative review)
**Grossmann et al., 2010** (42)	Evaluation of vehicle substances on vitamin D bioavailability: A systematic review	Wrong intervention (no staple food fortification)
**Guo et al., 2018** (43)	25(OH)D3-enriched or fortified foods are more efficient at tackling inadequate vitamin D status than vitamin D3	Wrong study design (narrative review)
**Haimi et al., 2017** (44)	Vitamin D deficiency/insufficiency from childhood to adulthood: Insights from a sunny country	Wrong study design (narrative review)
**Haridas et al., 2022** (45)	Micronutrient interventions among vulnerable population over a decade: A systematic review on Indian perspective	Wrong intervention (fortification not with vitamin D)
**Hayes et al., 2017** (46)	Food-based solutions for vitamin D deficiency: putting policy into practice and the key role for research	Wrong study design (narrative review)
**Hennessy et al., 2013** (47)	The impact of voluntary food fortification on micronutrient intakes and status in European countries: a review	Wrong study design (narrative review)
**Hiligsmann et al., 2017** (48)	A scoping review of the public health impact of vitamin D-fortified dairy products for fracture prevention	Wrong study design (scoping review)

**Holden et al., 2008** (49)	Vitamin D in foods: development of the US Department of Agriculture database	Wrong study design (primary study)
**Hossein-Nezhad et al., 2013** (50)	Vitamin D for health: A global perspective	Wrong study design (narrative review)
**Hung et al., 2023** (51)	Is Circulating Vitamin D Status Associated with the Risk of Venous Thromboembolism? A Meta-Analysis of Observational Studies	Wrong intervention (no staple food fortification)
**Huotari et al., 2008** (52)	Vitamin D and living in northern latitudes--an endemic risk area for vitamin D deficiency	Wrong study design (narrative review)
**Itkonen et al., 2021** (53)	Vitamin D status and current policies to achieve adequate vitamin D intake in the Nordic countries	Wrong study design (narrative review)
**Itkonen et al., 2018** (54)	Vitamin D Fortification of Fluid Milk Products and Their Contribution to Vitamin D Intake and Vitamin D Status in Observational Studies-A Review	No control group
**Johnson et al., 2006** (55)	Vitamin D, aging, and the 2005 Dietary Guidelines for Americans	Wrong study design (narrative review)
**Khayyatzadeh et al., 2019** (56)	What is the best solution to manage vitamin D deficiency?	Wrong study design (narrative review)
**Kiely et al., 2018** (57)	Summary Outcomes of the ODIN Project on Food Fortification for Vitamin D Deficiency Prevention	Wrong study design (narrative review)
**Kuang et al., 2020** (58)	The combination effect of vitamin K and vitamin D on human bone quality: a meta-analysis of randomized controlled trials	Wrong intervention (supplementation)
**Lamberg-Allardt et al., 2013** (59)	Vitamin D - a systematic literature review for the 5th edition of the Nordic Nutrition Recommendations	Wrong study design (overview of reviews)
**Mardiah et al., 2021** (60)	The Role of Vitamin D in Stunting Prevention: A Literature Review	wrong intervention (no staple food fortification)
**Marwaha et al., 2019** (61)	Bioavailability of nanoemulsion formulations vs conventional fat soluble preparations of cholecalciferol (D3) - An overview	Wrong intervention (no staple food fortification)
**Mazur et al., 2022** (62)	Vitamin D and Vitamin D3 Supplementation during Photodynamic Therapy: A Review	Wrong intervention (supplementation)
**McCourt et al., 2022** (63)	Using food fortification to improve vitamin D bioaccessibility and intakes	Wrong study design (narrative review)
**Méndez-Sánchez et al., 2023** (64)	Calcium and vitamin D for increasing bone mineral density in premenopausal women	Wrong intervention (supplementation)
**Michael et al., 2022** (65)	An Evidence-Based Review of Vitamin D for Common and High-Mortality Conditions	Wrong intervention (no staple food fortification)
**Mirhosseini et al., 2018** (66)	Vitamin D Supplementation, Serum 25(OH)D Concentrations and	Wrong intervention (supplementation)

	Cardiovascular Disease Risk Factors: A Systematic Review and Meta-Analysis	
**Morilla-Herrera et al., 2016** (67)	Effectiveness of Food-Based Fortification in Older People. A Systematic Review and Meta-Analysis	Wrong intervention (fortification not with vitamin D)
**Mosekilde et al., 2005** (68)	Vitamin D and the elderly	Wrong study design (narrative review)
**Mutgi et al., 2009** (69)	Vitamin D and breast cancer incidence: A meta-analysis	Wrong study population (people with a specific disease)
**Moulas et al., 2018** (70)	Vitamin D fortification of foods and prospective health outcomes	Wrong study design (narrative review)
**Mousa et al., 2016** (71)	Effect of Vitamin D supplementation on inflammation: Protocol for a systematic review	Wrong intervention (supplementation)
**Musazadeh et al., 2023** (72)	Vitamin D protects against depression: Evidence from an umbrella meta-analysis on interventional and observational meta-analyses	Wrong intervention (supplementation)
**Nakamura et al., 2006** (73)	Efficacy of optimization of vitamin D in preventing osteoporosis and osteoporotic fractures: A systematic review	Wrong intervention (no staple food fortification)
**Niedermaier et al., 2022** (74)	Vitamin D food fortification in European countries: the underused potential to prevent cancer deaths	Wrong study design (narrative review)
**Nikooyeh et al., 2022** (75)	Improvement of vitamin D status through consumption of either fortified food products or supplement pills increased hemoglobin concentration in adult subjects: Analysis of pooled data from two randomized clinical trials	Wrong study design (meta-analysis without a systematic search)
**Nowson et al., 2010** (76)	Prevention of fractures in older people with calcium and vitamin D	Wrong study design (narrative review)
**Obbagy et al., 2019** (77)	Types and Amounts of Complementary Foods and Beverages and Micronutrient Status: A Systematic Review	Wrong intervention (fortification not with vitamin D)<br><br>Duplicate
**Obbagy et al., 2019** (78)	Complementary feeding and micronutrient status: a systematic review	Wrong intervention (no staple food fortification)
**Okereke et al., 2016** (79)	The role of Vitamin D in the prevention of late-life depression	Wrong intervention (no staple food fortification)
**Ong et al., 2018** (80)	Association between fermented milk product intake and bone health in postmenopausal women: A systematic review	Wrong intervention (no staple food fortification)
**Panchal et al., 2022** (81)	Impact of Nutrition Interventions for Reduction of Anemia in Women of Reproductive Age in Low- and Middle-Income Countries: A Meta-Review	Wrong intervention (fortification not with vitamin D)

**Peña-Rosas et al., 2019** (82)	Fortification of rice with vitamins and minerals for addressing micronutrient malnutrition	Wrong intervention (fortification not with vitamin D)
**Pilz et al., 2018** (83)	Rationale and Plan for Vitamin D Food Fortification: A Review and Guidance Paper	Wrong study design (narrative review)
**Priebe et al., 2016** (84)	Effects of Ready-to-Eat-Cereals on Key Nutritional and Health Outcomes: A Systematic Review	Wrong intervention (fortification not with vitamin D)
**Pyo et al., 2022** (85)	Rice as a vehicle for micronutrient fortification: a systematic review of micronutrient retention, organoleptic properties, and consumer acceptability	Wrong intervention (fortification not with vitamin D)
**Rajwar et al., 2020** (86)	Effect of vitamin A, calcium and vitamin D fortification and supplementation on nutritional status of women: an overview of systematic reviews	Wrong study design (overview of reviews)
**Rehana et al., 2020** (87)	Effects of preventive nutrition interventions among adolescents on health and nutritional status in low- and middle-income countries	Wrong reporting (results of fortification and supplementation studies were analysed together)
**Reichrath et al., 2022** (88)	An Appraisal to Address Health Consequences of Vitamin D Deficiency With Food Fortification and Supplements: Time to Act!	Wrong study design (narrative review)
**Salam et al., 2020** (89)	Effects of preventive nutrition interventions among adolescents on health and nutritional status in low-and middle-income countries: A systematic review and meta-analysis	Wrong reporting (results of fortification and supplementation studies were analysed together)
**Salam et al., 2019** (90)	Effects of Preventive Nutrition Interventions among Adolescents on Health and Nutritional Status in Low- and Middle-Income Countries: A Systematic Review and Meta-Analysis	Wrong intervention (fortification not with vitamin D)
**Singh et al., 2018** (91)	Treatment of Vitamin D Deficiency and Comorbidities: A Review	Wrong study design (narrative review)
**Sundar et al., 2023** (92)	The role of Vitamin D as an adjunct for bone regeneration: A systematic review of literature	Wrong intervention (no staple food fortification)
**Tam et al., 2020** (93)	Micronutrient Supplementation and Fortification Interventions on Health and Development Outcomes among Children Under-Five in Low- and Middle-Income Countries: A Systematic Review and Meta-Analysis	Wrong intervention (fortification not with vitamin D)
**Tanna et al., 2021** (94)	Interventions to improve vitamin D status in at-risk ethnic groups during pregnancy and early childhood: a systematic review	Wrong intervention (no staple food fortification)

**Tomlinson et al., 2015** (95)	Effects of vitamin D supplementation on upper and lower body muscle strength levels in healthy individuals. A systematic review with meta-analysis	Wrong intervention (supplementation)
**Tomlinson et al., 2014** (96)	Effects of vitamin D supplementation on upper and lower body muscle strength levels in healthy individuals. A systematic review with meta-analysis	Wrong intervention (supplementation)
**Touvier et al., 2011** (97)	Meta-analyses of vitamin D intake, 25-hydroxyvitamin D status, vitamin D receptor polymorphisms, and colorectal cancer risk	Wrong intervention (no staple food fortification)
**Toyn et al., 2018** (98)	Effect of vitamin D2 supplementation on serum 25 hydroxy-vitamin D3 levels: A systematic review and meta-analysis	Wrong reporting (results of fortification and supplementation studies were analysed together)
**Urrutia et al., 2012** (99)	Vitamin D in pregnancy: Current concepts	Wrong intervention (supplementation)
**van den Heuvel et al., 2018** (100)	Dairy products and bone health: how strong is the scientific evidence?	Wrong study design (overview of reviews)
**Vieira et al., 2022** (101)	Formulation Strategies for Improving the Stability and Bioavailability of Vitamin D-Fortified Beverages: A Review	Wrong study design (narrative review)
**Vieth et al., 2017** (102)	Challenge and promise, the risks and benefits of vitamin d supplementation and fortification	Wrong study design (narrative review)
**von Websky et al., 2018** (103)	Impact of vitamin D on pregnancy-related disorders and on offspring outcome	Wrong study design (narrative review)
**Weaver et al., 2017** (104)	Nutrition and bone health	Wrong study design (narrative review)
**Wei et al., 2018** (105)	Associations of the risk of lung cancer with serum 25-hydroxyVitamin D level and dietary Vitamin D intake: A dose-response PRISMA meta-analysis	Wrong intervention (no staple food fortification)
**Whiting et al., 2016** (106)	Food fortification for bone health in adulthood: a scoping review	Wrong study design (scoping review)
**Wilson et al., 2017** (107)	Vitamin D deficiency as a public health issue: using vitamin D2 or vitamin D3 in future fortification strategies	Wrong study design (narrative review)
**Xu et al., 2021** (108)	Association between vitamin D/calcium intake and 25-hydroxyvitamin D and risk of ovarian cancer: a dose-response relationship meta-analysis	Wrong intervention (no staple food fortification)
**Yang et al., 2013** (109)	A review of vitamin D fortification: implications for nutrition programming in Southeast Asia	Wrong study design (narrative review)
**Yeh et al., 2017** (110)	Vitamin Fortification of Fluid Milk	Wrong study design (narrative review)

**Zhang et al., 2022** (111)	The effect of vitamin D on the lipid profile as a risk factor for coronary heart disease in postmenopausal women: a meta-analysis and systematic review of randomized controlled trials	Wrong intervention (supplementation)
**Zhang et al., 2020** (112)	Effects of vitamin D supplementation on prevention of type 2 diabetes in patients with prediabetes: A systematic review and meta-analysis	Wrong intervention (supplementation)
**Zhong et al., 2020** (113)	The influence of maternal vitamin D supplementation on infant vitamin D status: A systematic review and meta-analyses	Wrong intervention (supplementation)
**Zhou et al., 2009** (114)	Optimizing vitamin D status to reduce colorectal cancer risk: an evidentiary review	Wrong intervention (no staple food fortification)
**Zittermann et al., 2014** (115)	The role of vitamin D for cardiovascular disease and overall mortality	Wrong study design (narrative review)

References

1. Abrams SA, Atkinson SA. Calcium, magnesium, phosphorus and vitamin D fortification of complementary foods. Journal of Nutrition [Internet]. 2003;133(9):2994S-9S. Available from: https://ovidsp.ovid.com/ovidweb.cgi?T=JS&CSC=Y&NEWS=N&PAGE=fulltext&D=med5&AN=12949399

2. Al Khalifah R, Alsheikh R, Alsheikh R, Alhelali N, Naji A, Alnasser Y. The impact of vitamin D fortification of staple food for children: A systematic review and meta-analysis. Endocr Rev [Internet]. 2018;39(2). Available from: https://www.embase.com/search/results?subaction=viewrecord&id=L623114474&from=export

3. Andersen R, Brot C, Ovesen L. Towards a strategy for optimal vitamin D fortification (OPTIFORD). Nutrition Metabolism & Cardiovascular Diseases [Internet]. 2001;11(4 Suppl):74–7. Available from: https://ovidsp.ovid.com/ovidweb.cgi?T=JS&CSC=Y&NEWS=N&PAGE=fulltext&D=med4&AN=11894759

4. Balachandar R, Pullakhandam R, Kulkarni B, Sachdev HS. Relative Efficacy of Vitamin D2 and Vitamin D3 in Improving Vitamin D Status: Systematic Review and Meta-Analysis. Nutrients [Internet]. 2021;13(10):23. Available from: https://ovidsp.ovid.com/ovidweb.cgi?T=JS&CSC=Y&NEWS=N&PAGE=fulltext&D=med20&AN=34684328

5. Barbagallo M, Veronese N, Di Prazza A, Pollicino F, Carruba L, Carrubba AL, et al. Effect of Calcifediol on Physical Performance and Muscle Strength Parameters: A Systematic Review and Meta-Analysis. Nutrients [Internet]. 2022;14(9). Available from: https://www.embase.com/search/results?subaction=viewrecord&id=L2016565234&from=export

6. Beaudart C, Buckinx F, Rabenda V, Gillain S, Cavalier E, Slomian J, et al. The effects of vitamin d on skeletal muscle strength, muscle mass and muscle power: A meta-analysis of randomized controlled trials. Osteoporosis International [Internet]. 2014;25:S118-. Available from: https://www.embase.com/search/results?subaction=viewrecord&id=L71441215&from=export

7. Bendik I, Friedel A, Roos FF, Weber P, Eggersdorfer M. Vitamin D: a critical and essential micronutrient for human health. Front Physiol [Internet]. 2014;5:248. Available from: https://ovidsp.ovid.com/ovidweb.cgi?T=JS&CSC=Y&NEWS=N&PAGE=fulltext&D=pmnm3&AN=25071593

8. Bhutta Z, Prashad AJ, Wazny K, Keats E, Huynh A, A D, et al. Food fortification as a strategy for alleviating micronutrient deficiencies in low-and middle-income countries: A systematic review. Ann Glob Health [Internet]. 2015;81(1):188–9. Available from: https://www.embase.com/search/results?subaction=viewrecord&id=L72073865&from=export

9. Bischoff-Ferrari HA. Vitamin D Supplementation in Older Adults: Is the Hype Definitely Over? Deutsche Medizinische Wochenschrift [Internet]. 2019;144(15):1018–21. Available from: https://www.embase.com/search/results?subaction=viewrecord&id=L628694660&from=export

10. Bjelakovic G, Gluud LL, Nikolova D, Whitfield K, Wetterslev J, Simonetti RG, et al. Vitamin D supplementation for prevention of mortality in adults. Cochrane database of systematic reviews (Online) [Internet]. 2011;(7):CD007470-. Available from: https://www.embase.com/search/results?subaction=viewrecord&id=L362250812&from=export

11. Bolland M. Calcium and vitamin D: An update. Intern Med J [Internet]. 2014;44:9. Available from: https://www.embase.com/search/results?subaction=viewrecord&id=L614602281&from=export

12. Bonjour JP, Gueguen L, Palacios C, Shearer MJ, Weaver CM. Minerals and vitamins in bone health: the potential value of dietary enhancement. British Journal of Nutrition [Internet]. 2009;101(11):1581–96. Available from: https://ovidsp.ovid.com/ovidweb.cgi?T=JS&CSC=Y&NEWS=N&PAGE=fulltext&D=med7&AN=19335926

13. Brandi ML, Minisola S. Calcidiol [25(OH)D3]: from diagnostic marker to therapeutical agent. Curr Med Res Opin [Internet]. 2013;29(11):1565–72. Available from: https://ovidsp.ovid.com/ovidweb.cgi?T=JS&CSC=Y&NEWS=N&PAGE=fulltext&D=med10&AN=24020910

14. REICHRATH J, VOGT TH, HOLICK MF, FRIEDRICH M. ABSTRACTS OF THE JOINT INTERNATIONAL SYMPOSIA “VITAMIN D IN PREVENTION AND THERAPY” AND “BIOLOGIC EFFECTS OF LIGHT.” Anticancer Res. 2022 Apr 28;42(4):2193–222.

15. Caini S, Gnagnarella P, Stanganelli I, Bellerba F, Cocorocchio E, Queirolo P, et al. Vitamin d and the risk of non-melanoma skin cancer: A systematic literature review and meta-analysis on behalf of the italian melanoma intergroup. Cancers (Basel) [Internet]. 2021;13(19). Available from:

https://www.embase.com/search/results?subaction=viewrecord&id=L2013895374&from=export

16. Calvo MS, Whiting SJ, Barton CN. Vitamin D intake: a global perspective of current status. Journal of Nutrition [Internet]. 2005;135(2):310–6. Available from: https://ovidsp.ovid.com/ovidweb.cgi?T=JS&CSC=Y&NEWS=N&PAGE=fulltext&D=med6&AN=15671233

17. Calvo MS, Whiting SJ, Barton CN. Vitamin D fortification in the United States and Canada: current status and data needs. American Journal of Clinical Nutrition [Internet]. 2004;80(6 Suppl):1710S-6S. Available from: https://ovidsp.ovid.com/ovidweb.cgi?T=JS&CSC=Y&NEWS=N&PAGE=fulltext&D=med5&AN=15585792

18. Cashman KD, Ritz C. Individual participant data (IPD)-level meta-analysis of randomised controlled trials among dark-skinned populations to estimate the dietary requirement for vitamin D. Syst Rev. 2019 Dec 28;8(1):128.

19. Cashman KD, Kiely M. Tackling inadequate vitamin D intakes within the population: fortification of dairy products with vitamin D may not be enough. Endocrine [Internet]. 2016;51(1):38–46. Available from: https://www.embase.com/search/results?subaction=viewrecord&id=L605585062&from=export

20. Cashman KD, Kiely ME, Andersen R, Gronborg IM, Tetens I, Tripkovic L, et al. Individual participant data (IPD)-level meta-analysis of randomised controlled trials to estimate the vitamin D dietary requirements in dark-skinned individuals resident at high latitude. Eur J Nutr [Internet]. 2022;61(2):1015–34. Available from: https://ovidsp.ovid.com/ovidweb.cgi?T=JS&CSC=Y&NEWS=N&PAGE=fulltext&D=med21&AN=34705075

21. Chibuzor MT, Graham-Kalio D, Osaji JO, Meremikwu MM. Vitamin D, calcium or a combination of vitamin D and calcium for the treatment of nutritional rickets in children. Cochrane Database of Systematic Reviews [Internet]. 2020;2020(4). Available from: https://www.embase.com/search/results?subaction=viewrecord&id=L632455813&from=export

22. Chowdhury R, Kunutsor S, Vitezova A, Oliver-Williams C, Chowdhury S, Kiefte-De-Jong JC, et al. Vitamin D and risk of cause specific death: Systematic review and meta-analysis of observational cohort and randomised intervention studies. BMJ (Online) [Internet]. 2014;348. Available from: https://www.embase.com/search/results?subaction=viewrecord&id=L372801451&from=export

23. Chua JE, Gutierrez KM. The effect of vitamin D as adjunct to allergen immunotherapy (AIT): A systematic review and meta-analysis. Allergy: European Journal of Allergy and Clinical Immunology [Internet]. 2023;78:82–3. Available from: https://www.embase.com/search/results?subaction=viewrecord&id=L640977089&from=export

24. Curtis DM. Infant nutrient supplementation. Journal of Pediatrics [Internet]. 1990;117(2 Pt 2):S110-8. Available from:

https://ovidsp.ovid.com/ovidweb.cgi?T=JS&CSC=Y&NEWS=N&PAGE=fulltext&D=med3&AN=2199647

25. da Silva ABJ, Barros WMA, da Silva ML, Silva JML, Souza A, da Silva KG, et al. Impact of vitamin D on cognitive functions in healthy individuals: A systematic review in randomized controlled clinical trials. Front Psychol [Internet]. 2022;13:987203. Available from: https://ovidsp.ovid.com/ovidweb.cgi?T=JS&CSC=Y&NEWS=N&PAGE=fulltext&D=pmnm&AN=36524160

26. Darnton-Hill I. Public Health Aspects in the Prevention and Control of Vitamin Deficiencies. Curr Dev Nutr [Internet]. 2019;3(9):nzz075-. Available from: https://ovidsp.ovid.com/ovidweb.cgi?T=JS&CSC=Y&NEWS=N&PAGE=fulltext&D=pmnm4&AN=31598578

27. Darnton-Hill I, Darnton-Hill I, Nalubola R. Fortification strategies to meet micronutrient needs: successes and failures. Proceedings of the Nutrition Society [Internet]. 2002;61(2):231–41. Available from: https://ovidsp.ovid.com/ovidweb.cgi?T=JS&CSC=Y&NEWS=N&PAGE=fulltext&D=med4&AN=12133205

28. Das JK, Salam RA, Lassi ZS, Bhutta ZA, Kumar R, Moin A, et al. Food fortification with calcium and vitamin D: Impact on health outcomes. Cochrane Database of Systematic Reviews [Internet]. 2018;2018(8). Available from: https://www.embase.com/search/results?subaction=viewrecord&id=L623325242&from=export

29. Das JK, Salam RA, Mahmood SB, Moin A, Kumar R, Mukhtar K, et al. Food fortification with multiple micronutrients: impact on health outcomes in general population. Cochrane Database Syst Rev [Internet]. 2019;12:CD011400-. Available from: http://www.epistemonikos.org/documents/1eae3b0a62b5aa0d8d4c5ac42035f7d192e5274e

30. Datta M, Vitolins MZ. Food Fortification and Supplement Use-Are There Health Implications? Crit Rev Food Sci Nutr [Internet]. 2016;56(13):2149–59. Available from: https://ovidsp.ovid.com/ovidweb.cgi?T=JS&CSC=Y&NEWS=N&PAGE=fulltext&D=med13&AN=25036360

31. De-Regil LM, Palacios C, Ansary A, Kulier R, Peña-Rosas JP. Vitamin D supplementation for women during pregnancy. Cochrane database of systematic reviews (Online) [Internet]. 2012;2:CD008873-. Available from: https://www.embase.com/search/results?subaction=viewrecord&id=L364852570&from=export

32. Dewey K, Bazzano L, Davis T, Donovan S, Taveras E, Kleinman R, et al. Vitamin D from Supplements Consumed during Infancy and Toddlerhood and Bone Health: A Systematic Review [Internet]. USDA Nutrition Evidence Systematic Reviews. 2020. Available from: http://www.epistemonikos.org/documents/49c72fda3e4525d9780eb30ac2010ded982aac9d

33. Dötsch-Klerk M, Bruins MJ, Detzel P, Martikainen J, Nergiz-Unal R, Roodenburg AJC, et al. Modelling health and economic impact of nutrition interventions: a systematic review. Eur J Clin Nutr [Internet]. 2022; Available from: https://www.embase.com/search/results?subaction=viewrecord&id=L2019409247&from=export

34. Dunlop E, Kiely M, James AP, Singh T, Black LJ. Efficacy of vitamin D food fortification and biofortification in children and adults: a systematic review protocol. JBI Evid Synth. 2020 Dec;18(12):2694–703.

35. Eichler K, Wieser S, Rüthemann I, Brügger U. Effects of micronutrient fortified milk and cereal food for infants and children: a systematic review. BMC Public Health [Internet]. 2012;12(1):506. Available from: http://www.epistemonikos.org/documents/452320e74f314b8820104fc52f7d079cfed4cf28

36. El-Abbadi NH, Dao MC, Meydani SN. Yogurt: role in healthy and active aging. American Journal of Clinical Nutrition [Internet]. 2014;99(5 Suppl):1263S-70S. Available from: https://ovidsp.ovid.com/ovidweb.cgi?T=JS&CSC=Y&NEWS=N&PAGE=fulltext&D=med11&AN=24695886

37. Feehan O, Magee PJ, Pourshahidi LK, Armstrong DJ, McSorley EM. Vitamin D deficiency in nursing home residents: a systematic review. Nutr Rev [Internet]. 2022;11:11. Available from: https://ovidsp.ovid.com/ovidweb.cgi?T=JS&CSC=Y&NEWS=N&PAGE=fulltext&D=medp&AN=36367832

38. Fraile Navarro D, Lopez Garcia-Franco A, Nino de Guzman E, Rabassa M, Zamanillo Campos R, Pardo-Hernandez H, et al. Vitamin D recommendations in clinical guidelines: A systematic review, quality evaluation and analysis of potential predictors. Int J Clin Pract [Internet]. 2021;75(11):e14805-. Available from: https://ovidsp.ovid.com/ovidweb.cgi?T=JS&CSC=Y&NEWS=N&PAGE=fulltext&D=med20&AN=34486779

39. G R, Gupta A. Fortification of foods with vitamin D in India. Nutrients [Internet]. 2014;6(9):3601–23. Available from: https://ovidsp.ovid.com/ovidweb.cgi?T=JS&CSC=Y&NEWS=N&PAGE=fulltext&D=med11&AN=25221975

40. Grant WB, Boucher BJ. A Review of the Potential Benefits of Increasing Vitamin D Status in Mongolian Adults through Food Fortification and Vitamin D Supplementation. Nutrients [Internet]. 2019;11(10):14. Available from: https://ovidsp.ovid.com/ovidweb.cgi?T=JS&CSC=Y&NEWS=N&PAGE=fulltext&D=med16&AN=31615079

41. Grant WB, Cross HS, Garland CF, Gorham ED, Moan J, Peterlik M, et al. Estimated benefit of increased vitamin D status in reducing the economic burden of disease in western Europe. Prog Biophys Mol Biol [Internet]. 2009;99(2–3):104–13. Available from: https://ovidsp.ovid.com/ovidweb.cgi?T=JS&CSC=Y&NEWS=N&PAGE=fulltext&D=med7&AN=19268496

42. Grossmann RE, Tangpricha V. Evaluation of vehicle substances on vitamin D bioavailability: A systematic review. Mol Nutr Food Res [Internet]. 2010;54(8):1055–61. Available from: https://www.embase.com/search/results?subaction=viewrecord&id=L359405861&from=export

43. Guo J, Lovegrove JA, Givens DI. 25(OH)D3-enriched or fortified foods are more efficient at tackling inadequate vitamin D status than vitamin D3. Proceedings of the Nutrition Society [Internet]. 2018;77(3):282–91. Available from:

https://ovidsp.ovid.com/ovidweb.cgi?T=JS&CSC=Y&NEWS=N&PAGE=fulltext&D=med15&AN=29173203

44. Haimi M, Kremer R. Vitamin D deficiency/insufficiency from childhood to adulthood: Insights from a sunny country. World J Clin Pediatr [Internet]. 2017;6(1):1–9. Available from: https://ovidsp.ovid.com/ovidweb.cgi?T=JS&CSC=Y&NEWS=N&PAGE=fulltext&D=pmnm4&AN=28224090

45. Haridas S, Ramaswamy J, Natarajan T, Nedungadi P. Micronutrient interventions among vulnerable population over a decade: A systematic review on Indian perspective. Health Promot Perspect [Internet]. 2022;12(2):151–62. Available from: http://www.epistemonikos.org/documents/2657e0cb7a7cc75d6d865e5f6780097261a2679e

46. Hayes A, Cashman KD. Food-based solutions for vitamin D deficiency: putting policy into practice and the key role for research. Proceedings of the Nutrition Society [Internet]. 2017;76(1):54–63. Available from: https://ovidsp.ovid.com/ovidweb.cgi?T=JS&CSC=Y&NEWS=N&PAGE=fulltext&D=med14&AN=27776564

47. Hennessy A, Walton J, Flynn A. The impact of voluntary food fortification on micronutrient intakes and status in European countries: a review. Proceedings of the Nutrition Society [Internet]. 2013;72(4):433–40. Available from: https://ovidsp.ovid.com/ovidweb.cgi?T=JS&CSC=Y&NEWS=N&PAGE=fulltext&D=med10&AN=24020749

48. Hiligsmann M, Neuprez A, Buckinx F, Locquet M, Reginster JY. A scoping review of the public health impact of vitamin D-fortified dairy products for fracture prevention. Arch Osteoporos. 2017 Dec 21;12(1):57.

49. Holden JM, Lemar LE, Exler J. Vitamin D in foods: development of the US Department of Agriculture database. American Journal of Clinical Nutrition [Internet]. 2008;87(4):1092S-6S. Available from: https://ovidsp.ovid.com/ovidweb.cgi?T=JS&CSC=Y&NEWS=N&PAGE=fulltext&D=med7&AN=18400740

50. Hossein-Nezhad A, Holick MF. Vitamin D for health: A global perspective. Mayo Clin Proc [Internet]. 2013;88(7):720–55. Available from: https://www.embase.com/search/results?subaction=viewrecord&id=L52638926&from=export

51. Hung KC, Yang SH, Chang CY, Wang LK, Lin YT, Yu CH, et al. Is Circulating Vitamin D Status Associated with the Risk of Venous Thromboembolism? A Meta-Analysis of Observational Studies. Nutrients [Internet]. 2023;15(5). Available from: https://www.embase.com/search/results?subaction=viewrecord&id=L2022026133&from=export

52. Huotari A, Herzig KH. Vitamin D and living in northern latitudes--an endemic risk area for vitamin D deficiency. Int J Circumpolar Health [Internet]. 2008;67(2–3):164–78. Available from: https://ovidsp.ovid.com/ovidweb.cgi?T=JS&CSC=Y&NEWS=N&PAGE=fulltext&D=med7&AN=18767337

53. Itkonen ST, Andersen R, Bjork AK, Brugard Konde A, Eneroth H, Erkkola M, et al. Vitamin D status and current policies to achieve adequate vitamin D intake in the Nordic countries. Scand J Public Health [Internet]. 2021;49(6):616–27. Available from: https://ovidsp.ovid.com/ovidweb.cgi?T=JS&CSC=Y&NEWS=N&PAGE=fulltext&D=med19&AN=31916497

54. Itkonen ST, Erkkola M, Lamberg-Allardt CJE. Vitamin D Fortification of Fluid Milk Products and Their Contribution to Vitamin D Intake and Vitamin D Status in Observational Studies-A Review. Nutrients [Internet]. 2018;10(8):9. Available from: https://ovidsp.ovid.com/ovidweb.cgi?T=JS&CSC=Y&NEWS=N&PAGE=fulltext&D=med15&AN=30096919

55. Johnson MA, Kimlin MG. Vitamin D, aging, and the 2005 Dietary Guidelines for Americans. Nutr Rev [Internet]. 2006;64(9):410–21. Available from: https://ovidsp.ovid.com/ovidweb.cgi?T=JS&CSC=Y&NEWS=N&PAGE=fulltext&D=med6&AN=17002237

56. Khayyatzadeh SS, Bagherniya M, Abdollahi Z, Ferns GA, Ghayour-Mobarhan M. What is the best solution to manage vitamin D deficiency? IUBMB Life [Internet]. 2019;71(9):1190–1. Available from: https://ovidsp.ovid.com/ovidweb.cgi?T=JS&CSC=Y&NEWS=N&PAGE=fulltext&D=med16&AN=30932323

57. Kiely M, Cashman KD. Summary Outcomes of the ODIN Project on Food Fortification for Vitamin D Deficiency Prevention. International Journal of Environmental Research & Public Health [Electronic Resource] [Internet]. 2018;15(11):24. Available from: https://ovidsp.ovid.com/ovidweb.cgi?T=JS&CSC=Y&NEWS=N&PAGE=fulltext&D=med15&AN=30352957

58. Kuang X, Liu C, Guo X, Li K, Deng Q, Li D. The combination effect of vitamin K and vitamin D on human bone quality: a meta-analysis of randomized controlled trials. Food Funct [Internet]. 2020;11(4):3280–97. Available from: https://www.embase.com/search/results?subaction=viewrecord&id=L631354340&from=export

59. Lamberg-Allardt C, Brustad M, Meyer HE, Steingrimsdottir L. Vitamin D - a systematic literature review for the 5th edition of the Nordic Nutrition Recommendations. Food Nutr Res [Internet]. 2013;57. Available from: https://ovidsp.ovid.com/ovidweb.cgi?T=JS&CSC=Y&NEWS=N&PAGE=fulltext&D=pmnm3&AN=24106457

60. Mardiah W, Setiabudiawan B, Mediani HS. The Role of Vitamin D in Stunting Prevention: A Literature Review. Open Access Maced J Med Sci [Internet]. 2021;9(T6):85–91. Available from: https://www.embase.com/search/results?subaction=viewrecord&id=L2015447963&from=export

61. Marwaha RK, Dabas A. Bioavailability of nanoemulsion formulations vs conventional fat soluble preparations of cholecalciferol (D3) - An overview. J Clin Orthop Trauma [Internet]. 2019;10(6):1094–6. Available from: https://ovidsp.ovid.com/ovidweb.cgi?T=JS&CSC=Y&NEWS=N&PAGE=fulltext&D=pmnm4&AN=31708634

62. Mazur A, Koziorowska K, Dynarowicz K, Aebisher D, Bartusik-Aebisher D. Vitamin D and Vitamin D3 Supplementation during Photodynamic Therapy: A Review. Nutrients [Internet]. 2022;14(18):15. Available from: https://ovidsp.ovid.com/ovidweb.cgi?T=JS&CSC=Y&NEWS=N&PAGE=fulltext&D=med22&AN=36145180

63. McCourt AF, O'Sullivan AM. Using food fortification to improve vitamin D bioaccessibility and intakes. Proceedings of the Nutrition Society [Internet]. 2022;81(1):99–107. Available from: https://ovidsp.ovid.com/ovidweb.cgi?T=JS&CSC=Y&NEWS=N&PAGE=fulltext&D=med21&AN=35197143

64. Méndez-Sánchez L, Clark P, Winzenberg TM, Tugwell P, Correa-Burrows P, Costello R. Calcium and vitamin D for increasing bone mineral density in premenopausal women. Cochrane Database of Systematic Reviews [Internet]. 2023;2023(1). Available from: https://www.embase.com/search/results?subaction=viewrecord&id=L640144709&from=export

65. Michael W, Couture AD, Swedlund M, Hampton A, Eglash A, Schrager S. An Evidence-Based Review of Vitamin D for Common and High-Mortality Conditions. Journal of the American Board of Family Medicine [Internet]. 2022;35(6):1217–29. Available from: https://www.embase.com/search/results?subaction=viewrecord&id=L2022340788&from=export

66. Mirhosseini N, Rainsbury J, Kimball SM. Vitamin D Supplementation, Serum 25(OH)D Concentrations and Cardiovascular Disease Risk Factors: A Systematic Review and Meta-Analysis. Front Cardiovasc Med [Internet]. 2018;5. Available from: https://www.embase.com/search/results?subaction=viewrecord&id=L630821186&from=export

67. Morilla-Herrera JC, Martín-Santos FJ, Caro-Bautista J, Saucedo-Figueredo C, García-Mayor S, Morales-Asencio JM. Effectiveness of Food-Based Fortification in Older People. A Systematic Review and Meta-Analysis. J Nutr Health Aging [Internet]. 2016;20(2):178–84. Available from: http://www.epistemonikos.org/documents/7d1e892583df1958729ceb3ace092038cfbfbccc

68. Mosekilde L. Vitamin D and the elderly. Clin Endocrinol (Oxf) [Internet]. 2005;62(3):265–81. Available from: https://ovidsp.ovid.com/ovidweb.cgi?T=JS&CSC=Y&NEWS=N&PAGE=fulltext&D=med6&AN=15730407

69. Mutgi AB, Kaushik G, Mohamed I, Khuder SA, Salameh J. Vitamin D and breast cancer incidence: A meta-analysis. Cancer Res [Internet]. 2009;69(2). Available from: https://www.embase.com/search/results?subaction=viewrecord&id=L70108228&from=export

70. Moulas AN, Vaiou M. Vitamin D fortification of foods and prospective health outcomes. J Biotechnol [Internet]. 2018;285:91–101. Available from: https://ovidsp.ovid.com/ovidweb.cgi?T=JS&CSC=Y&NEWS=N&PAGE=fulltext&D=med15&AN=30176270

71. Mousa A, Misso M, Teede H, Scragg R, De Courten B. Effect of Vitamin D supplementation on inflammation: Protocol for a systematic review. BMJ Open [Internet]. 2016;6(4). Available from:

https://www.embase.com/search/results?subaction=viewrecord&id=L610190234&from=export

72. Musazadeh V, Keramati M, Ghalichi F, Kavyani Z, Ghoreishi Z, Alras KA, et al. Vitamin D protects against depression: Evidence from an umbrella meta-analysis on interventional and observational meta-analyses. Pharmacol Res [Internet]. 2023;187. Available from: https://www.embase.com/search/results?subaction=viewrecord&id=L2021864766&from=export

73. Nakamura K, Masayuki I. Efficacy of optimization of vitamin D in preventing osteoporosis and osteoporotic fractures: A systematic review. Environ Health Prev Med [Internet]. 2006;11(4):155–70. Available from: https://www.embase.com/search/results?subaction=viewrecord&id=L44186435&from=export

74. Niedermaier T, Gredner T, Kuznia S, Schottker B, Mons U, Lakerveld J, et al. Vitamin D food fortification in European countries: the underused potential to prevent cancer deaths. Eur J Epidemiol [Internet]. 2022;37(4):309–20. Available from: https://ovidsp.ovid.com/ovidweb.cgi?T=JS&CSC=Y&NEWS=N&PAGE=fulltext&D=med22&AN=35524028

75. Nikooyeh B, Zahedirad M, Kalayi A, Shariatzadeh N, Hollis BW, Neyestani TR. Improvement of vitamin D status through consumption of either fortified food products or supplement pills increased hemoglobin concentration in adult subjects: Analysis of pooled data from two randomized clinical trials. Nutr Health [Internet]. 2022;2601060221085351-. Available from: https://www.embase.com/search/results?subaction=viewrecord&id=L637410471&from=export

76. Nowson CA. Prevention of fractures in older people with calcium and vitamin D. Nutrients [Internet]. 2010;2(9):975–84. Available from: https://ovidsp.ovid.com/ovidweb.cgi?T=JS&CSC=Y&NEWS=N&PAGE=fulltext&D=med8&AN=22254066

77. Obbagy JE, English LK, Psota TL, Nadaud P, Johns K, Wong YP, et al. Types and Amounts of Complementary Foods and Beverages and Micronutrient Status: A Systematic Review [Internet]. USDA Nutrition Evidence Systematic Reviews. 2019. Available from: http://www.epistemonikos.org/documents/d4cee2ff393d273c294e239a6a69749f14b7cd3a

78. Obbagy JE, English LK, Psota TL, Wong YP, Butte NF, Dewey KG, et al. Complementary feeding and micronutrient status: a systematic review. American Journal of Clinical Nutrition [Internet]. 2019;109(Suppl_7):852S-871S. Available from: https://ovidsp.ovid.com/ovidweb.cgi?T=JS&CSC=Y&NEWS=N&PAGE=fulltext&D=med16&AN=30982869

79. Okereke OI, Singh A. The role of Vitamin D in the prevention of late-life depression. J Affect Disord [Internet]. 2016;198:1–14. Available from: https://www.embase.com/search/results?subaction=viewrecord&id=L609078545&from=export

80. Ong A, Weiler H, Morin S, Kang K. Association between fermented milk product intake and bone health in postmenopausal women: A systematic review. Journal of Bone and Mineral Research [Internet]. 2018;33:279. Available from:

https://www.embase.com/search/results?subaction=viewrecord&id=L631814178&from=export

81. Panchal PD, Ravalia A, Rana R, Puthussery S, Vaze G, Mavlankar D, et al. Impact of Nutrition Interventions for Reduction of Anemia in Women of Reproductive Age in Low- and Middle-Income Countries: A Meta-Review. Curr Dev Nutr [Internet]. 2022;6(12):nzac134-. Available from: http://www.epistemonikos.org/documents/5364e3eb3d710f5626ad34623061295b7eccaf8b

82. Peña-Rosas JP, Mithra P, Unnikrishnan B, Kumar N, De-Regil LM, Nair NS, et al. Fortification of rice with vitamins and minerals for addressing micronutrient malnutrition. Cochrane Database Syst Rev [Internet]. 2019;2019(10). Available from: http://www.epistemonikos.org/documents/cccd63deaab650206318a96fba101f2d2b898152

83. Pilz S, Marz W, Cashman KD, Kiely ME, Whiting SJ, Holick MF, et al. Rationale and Plan for Vitamin D Food Fortification: A Review and Guidance Paper. Front Endocrinol (Lausanne) [Internet]. 2018;9:373. Available from: https://ovidsp.ovid.com/ovidweb.cgi?T=JS&CSC=Y&NEWS=N&PAGE=fulltext&D=pmnm4&AN=30065699

84. Priebe MG, McMonagle JR. Effects of Ready-to-Eat-Cereals on Key Nutritional and Health Outcomes: A Systematic Review. PLoS ONE [Electronic Resource] [Internet]. 2016;11(10):e0164931-. Available from: http://www.epistemonikos.org/documents/d9ca2ede0881813348730c390417aeab4b72fced

85. Pyo E, Tsang BL, Parker ME. Rice as a vehicle for micronutrient fortification: a systematic review of micronutrient retention, organoleptic properties, and consumer acceptability. Nutr Rev [Internet]. 2022; Available from: http://www.epistemonikos.org/documents/2b5f1b9022d7519b8ab16c61003c6d0b8f46f996

86. Rajwar E, Parsekar SS, Venkatesh BT, Sharma Z. Effect of vitamin A, calcium and vitamin D fortification and supplementation on nutritional status of women: an overview of systematic reviews. Syst Rev [Internet]. 2020;9(1):248. Available from: https://ovidsp.ovid.com/ovidweb.cgi?T=JS&CSC=Y&NEWS=N&PAGE=fulltext&D=med18&AN=33109248

87. Rehana AS, Jai K Das, Omar I, Wardah A, Sana SS, Zulfiqar AB. Effects of preventive nutrition interventions among adolescents on health and nutritional status in low- and middle-income countries. Campbell Collaboration [Internet]. 2020; Available from: http://www.epistemonikos.org/documents/719d12f8dcaafa7e0b3e5d6869c66eb31ecf58ad

88. Reichrath J, März W, de Gruijl FR, Vieth R, Grant WB, Slominski AT, et al. An Appraisal to Address Health Consequences of Vitamin D Deficiency With Food Fortification and Supplements: Time to Act! Anticancer Res [Internet]. 2022;42(10):5009–15. Available from: https://www.embase.com/search/results?subaction=viewrecord&id=L2020563248&from=export

89. Salam RA, Das JK, Ahmed W, Irfan O, Bhutta ZA, Sheikh SS. Effects of preventive nutrition interventions among adolescents on health and nutritional status in low-and middle-income countries: A systematic review and meta-analysis. Nutrients [Internet]. 2020;12(1). Available from:

https://www.embase.com/search/results?subaction=viewrecord&id=L2003420861&from=export

90. Salam RA, Das JK, Ahmed W, Irfan O, Sheikh SS, Bhutta ZA. Effects of Preventive Nutrition Interventions among Adolescents on Health and Nutritional Status in Low- and Middle-Income Countries: A Systematic Review and Meta-Analysis. Nutrients [Internet]. 2019;12(1):23. Available from: https://ovidsp.ovid.com/ovidweb.cgi?T=JS&CSC=Y&NEWS=N&PAGE=fulltext&D=med16&AN=31878019

91. Singh P. Treatment of Vitamin D Deficiency and Comorbidities: A Review. Journal of the Association of Physicians of India [Internet]. 2018;66(1):75–82. Available from: https://ovidsp.ovid.com/ovidweb.cgi?T=JS&CSC=Y&NEWS=N&PAGE=fulltext&D=med15&AN=30341848

92. Sundar R, Bhagavandas Rai A, Naveen Kumar J, Devang Divakar D. The role of Vitamin D as an adjunct for bone regeneration: A systematic review of literature. Saudi Dental Journal [Internet]. 2023; Available from: https://www.embase.com/search/results?subaction=viewrecord&id=L2023294054&from=export

93. Tam E, Keats EC, Rind F, Das JK, Bhutta AZA. Micronutrient Supplementation and Fortification Interventions on Health and Development Outcomes among Children Under-Five in Low- and Middle-Income Countries: A Systematic Review and Meta-Analysis. Nutrients [Internet]. 2020;12(2). Available from: http://www.epistemonikos.org/documents/05ee01c92f90637471e0666805d21888e51699df

94. Tanna NK, Alexander EC, Lee C, Lakhanpaul M, Popat RM, Almeida-Meza P, et al. Interventions to improve vitamin D status in at-risk ethnic groups during pregnancy and early childhood: a systematic review. Public Health Nutr [Internet]. 2021;24(11):3498–519. Available from: https://www.embase.com/search/results?subaction=viewrecord&id=L634323251&from=export

95. Tomlinson PB, Joseph C, Angioi M. Effects of vitamin D supplementation on upper and lower body muscle strength levels in healthy individuals. A systematic review with meta-analysis. J Sci Med Sport [Internet]. 2015;18(5):575–80. Available from: https://www.embase.com/search/results?subaction=viewrecord&id=L605539964&from=export

96. Tomlinson PB, Joseph C, Angioi M. Effects of vitamin D supplementation on upper and lower body muscle strength levels in healthy individuals. A systematic review with meta-analysis. J Sci Med Sport [Internet]. 2014; Available from: https://www.embase.com/search/results?subaction=viewrecord&id=L53303444&from=export

97. Touvier M, Chan DSM, Lau R, Aune D, Vieira R, Greenwood DC, et al. Meta-analyses of vitamin D intake, 25-hydroxyvitamin D status, vitamin D receptor polymorphisms, and colorectal cancer risk. Cancer Epidemiology Biomarkers and Prevention [Internet]. 2011;20(5):1003–16. Available from: https://www.embase.com/search/results?subaction=viewrecord&id=L361726691&from=export

98. Toyn C, Darling AL, Hart K, Tripkovic L, Smith CP, Mathers JC, et al. Effect of vitamin D2 supplementation on serum 25 hydroxy-vitamin D3 levels: A systematic review and meta-analysis. Proceedings of the Nutrition Society [Internet]. 2018;77(OCE1). Available from: https://www.embase.com/search/results?subaction=viewrecord&id=L621003837&from=export

99. Urrutia RP, Thorp JM. Vitamin D in pregnancy: Current concepts. Curr Opin Obstet Gynecol [Internet]. 2012;24(2):57–64. Available from: https://www.embase.com/search/results?subaction=viewrecord&id=L51856100&from=export

100. van den Heuvel E, Steijns J. Dairy products and bone health: how strong is the scientific evidence? Nutr Res Rev [Internet]. 2018;31(2):164–78. Available from: https://ovidsp.ovid.com/ovidweb.cgi?T=JS&CSC=Y&NEWS=N&PAGE=fulltext&D=med15&AN=29560832

101. Vieira EF, Souza S. Formulation Strategies for Improving the Stability and Bioavailability of Vitamin D-Fortified Beverages: A Review. Foods [Internet]. 2022;11(6):16. Available from: https://ovidsp.ovid.com/ovidweb.cgi?T=JS&CSC=Y&NEWS=N&PAGE=fulltext&D=pmnm7&AN=35327269

102. Vieth R. CHALLENGE AND PROMISE, THE RISKS AND BENEFITS OF VITAMIN D SUPPLEMENTATION AND FORTIFICATION. Anticancer Res [Internet]. 2017;37(6):3346. Available from: https://www.embase.com/search/results?subaction=viewrecord&id=L640666096&from=export

103. von Websky K, Hasan AA, Reichetzeder C, Tsuprykov O, Hocher B. Impact of vitamin D on pregnancy-related disorders and on offspring outcome. Journal of Steroid Biochemistry and Molecular Biology [Internet]. 2018;180:51–64. Available from: https://www.embase.com/search/results?subaction=viewrecord&id=L619422667&from=export

104. Weaver CM. Nutrition and bone health. Oral Dis [Internet]. 2017;23(4):412–5. Available from: https://ovidsp.ovid.com/ovidweb.cgi?T=JS&CSC=Y&NEWS=N&PAGE=fulltext&D=med14&AN=27250737

105. Wei H, Jing H, Wei Q, Wei G, Heng Z. Associations of the risk of lung cancer with serum 25-hydroxyVitamin D level and dietary Vitamin D intake: A dose-response PRISMA meta-analysis. Medicine (United States) [Internet]. 2018;97(37). Available from: https://www.embase.com/search/results?subaction=viewrecord&id=L627405877&from=export

106. Whiting SJ, Kohrt WM, Warren MP, Kraenzlin MI, Bonjour JP. Food fortification for bone health in adulthood: a scoping review. Eur J Clin Nutr [Internet]. 2016;70(10):1099–105. Available from: https://ovidsp.ovid.com/ovidweb.cgi?T=JS&CSC=Y&NEWS=N&PAGE=fulltext&D=med13&AN=27026430

107. Wilson LR, Tripkovic L, Hart KH, Lanham-New SA. Vitamin D deficiency as a public health issue: using vitamin D2 or vitamin D3 in future fortification strategies. Proceedings of the Nutrition Society [Internet]. 2017;76(3):392–9. Available from:

https://ovidsp.ovid.com/ovidweb.cgi?T=JS&CSC=Y&NEWS=N&PAGE=fulltext&D=med14&AN=28347378

108. Xu J, Chen K, Zhao F, Huang D, Zhang H, Fu Z, et al. Association between vitamin D/calcium intake and 25-hydroxyvitamin D and risk of ovarian cancer: a dose-response relationship meta-analysis. Eur J Clin Nutr [Internet]. 2021;75(3):417–29. Available from: https://www.embase.com/search/results?subaction=viewrecord&id=L2005903477&from=export

109. Yang Z, Laillou A, Smith G, Schofield D, Moench-Pfanner R. A review of vitamin D fortification: implications for nutrition programming in Southeast Asia. Food Nutr Bull [Internet]. 2013;34(2 Suppl):S81-9. Available from: https://ovidsp.ovid.com/ovidweb.cgi?T=JS&CSC=Y&NEWS=N&PAGE=fulltext&D=med10&AN=24049999

110. Yeh EB, Barbano DM, Drake M. Vitamin Fortification of Fluid Milk. J Food Sci [Internet]. 2017;82(4):856–64. Available from: https://ovidsp.ovid.com/ovidweb.cgi?T=JS&CSC=Y&NEWS=N&PAGE=fulltext&D=med14&AN=28253423

111. Zhang W, Yi J, Liu D, Wang Y, Jamilian P, Gaman MA, et al. The effect of vitamin D on the lipid profile as a risk factor for coronary heart disease in postmenopausal women: a meta-analysis and systematic review of randomized controlled trials. Exp Gerontol [Internet]. 2022;161. Available from: https://www.embase.com/search/results?subaction=viewrecord&id=L2016655489&from=export

112. Zhang Y, Tan H, Tang J, Li J, Chong W, Hai Y, et al. Effects of vitamin D supplementation on prevention of type 2 diabetes in patients with prediabetes: A systematic review and meta-analysis. Diabetes Care [Internet]. 2020;43(7):1650–8. Available from: https://www.embase.com/search/results?subaction=viewrecord&id=L2004950254&from=export

113. Zhong X, Xiong Y, Wei D, Wang S, Xiao Z, Liu M, et al. The influence of maternal vitamin D supplementation on infant vitamin D status: A systematic review and meta-analyses. Complement Ther Med [Internet]. 2020;52. Available from: https://www.embase.com/search/results?subaction=viewrecord&id=L2006884002&from=export

114. Zhou G, Stoitzfus J, Swan BA. Optimizing vitamin D status to reduce colorectal cancer risk: an evidentiary review. Clin J Oncol Nurs [Internet]. 2009;13(4):E3–17. Available from: https://www.embase.com/search/results?subaction=viewrecord&id=L355899643&from=export

115. Zittermann A, Prokop S. The role of vitamin D for cardiovascular disease and overall mortality [Internet]. Vol. 810. 2014. p. 106–19. Available from: https://www.embase.com/search/results?subaction=viewrecord&id=L615954191&from=export
