# Supplementary material for: Fortification of Staple Foods for Household Use with Vitamin D: An Overview of Systematic Reviews
Source: Nutrients. 2023 Aug 26;15(17):3742. doi: 10.3390/nu15173742 (PMC10489979; doi:10.3390/nu15173742)
Supplement: Supplementary file 1 [file nutrients-15-03742-s001.zip › Supplementary_File_S5_Primary_studies_included_in_the_systematic_reviews.pdf]

	Aguiar 2017 (#42)	Al Khalifah 2020 (#61)	Black 2012 (#273)	Brandão-Lima 2019 (#319)	Brett 2018 (#323)	Brooker 2022 (#329)	Cashman 2021 (#395)	Cranney 2007 (#511)	Cranney 2008 (#512)	Das 2013 (#560)	Dunlop 2021 (#664)	Emadzadeh 2022 (#689)	Emadzadeh 2020 (#690)	Emadzadeh 2020 (#691)	Fonseca Santos 2022 (#750)	Gasparri 2019 (#801)	Lam 2016 (#1263)	Niedermaier 2021 (#1683)	Nikooyeh 2018 (#1694)	Nikooyeh 2022 (#1692)	Nikooyeh 2022 (#1696)	O'Donnell 2008 (#1723)	O'Mahony 2011 (#1732)	Soto-Mendez 2019 (#2133)	Souza 2022 (#2138)	Tangestani 2020 (#2216)	Whiting 2015 (#2419)	Number of inclusion in systematic reviews
Adolphi et al. (2009)										x																		1
Akkermans et al. (2017)		x				x																						2
Al-Daghri et al. (2019)																				x								1
Battiprolu et al. (2006)		x																										1
Benjeddou et al. (2019)		x																		x								2
Biancuzzo et al. (2010)			x								x							x			x		x					5
Bonjour et al. (1997)										x																		1
Bonjour et al. (2009)										x					x		x									x		4
Bonjour et al. (2011)															x		x											2
Bonjour et al. (2012)															x						x					x		3
Bonjour et al. (2013)											x	x			x	x	x				x					x	x	8
Bonjour et al. (2015)											x	x			x	x					x					x		6
Bonjour et al. (2018)												x									x							2
Brett et al. (2016)		x		x	x		x				x	x								x								7
Brett et al. (2018)		x		x							x	x	x															5
Chee et al. (2003)			x					x		x												x	x					5
Costan, Vulpoi, and Mocanu (2014)																	x								x	x		3
Daly et al. (2006b)			x					x														x	x			x		5
Daly et al. (2008)													x															1
Daly et al. (2009)																					x							1
Daly et al. (2006a)												x																1
de Jong et al. (1999)			x					x			x											x						4
Du et al. (2004)		x			x					x	x	x	x															6
Economos et al. (2014)		x			x						x									x								4
Fisk et al. (2012)											x	x																2
Gaffney-Stomberg et al. (2014)											x																	1
Gaffney-Stomberg et al. (2019)													x															1
Ganmaa et al. (2017)																				x								1
Ganmaa et al. (2008)										x										x								2
Gasparri et al. (2019)																		x										1
Graham et al. (2009)		x								x																		2
Green et al. (2010)			x							x	x	x									x							5
Grønborg et al. (2019)											x	x	x												x			4
Grønborg et al. (2020)												x									x				x			3
Hayes et al. (2016)							x																					1
Hennigar et al. (2015)													x															1
Heravifard et al. (2013)														x														1
Hettiarachchi et al. (2010)		x			x															x								3
Ho et al. (2005)										x																		1
Houghton et al. (2011)		x				x																						2
Hower et al. (2013)		x		x	x		x				x									x								6
Itkonen et al. (2016)											x	x						x							x			4
Jaaskelainen et al. (2017)																		x										1
Jafari et al. (2016)											x	x	x	x		x			x		x					x		8
Jakobsen & Knuthsen et al. (2014)																									x			1
Johnson et al. (2005)							x	x				x	x								x	x	x					7

Kanellakis et al. (2012)											x	x															2
Keane et al. (1998)			x					x			x							x				x						5
Keane et al. (1992)												x																1
Khadgawat et al. (2013)		x									x	x						x		x								5
Kruger et al. (2006)										x																		1
Kruger et al. (2010)			x							x	x																	3
Kruger et al. (2012)											x																	1
Kruger et al. (2018)											x										x							2
Kruger et al. (2019)											x							x										2
Kukuljan et al. (2009a)													x															1
Kukuljan et al. (2009b)			x																		x							2
Kukuljan et al. (2011)												x														x		2
Kuriyan et al. (2016)		x																										1
Lau et al (2001)								x		x												x						3
Lehtonen-Veromaa et al. (2008)										x																		1
Li and Xing et al. (2016)											x					x								x				3
Lovell et al. (2018)						x																						1
Lu et al. (2019)												x																1
Madsen et al. (2013)		x			x		x											x							x			5
Manios et al. (2007)												x																1
Manios et al. (2009)			x							x		x														x		4
Manios et al. (2011)												x																1
Manios et al. (2017)											x										x							2
McKenna et al. (1995)			x					x										x				x						4
Mocanu & Vieth et al. (2013)																	x								x			2
Mocanu et al. (2009)																	x								x	x		3
Mohammadi-Sartang et al. (2018)											x					x												2
Moreira-Lucas et al. (2016)												x	x															2
Moreira-Lucas et al. (2017)													x	x														2
Moschonis and Manios et al. (2006)										x		x	x													x		4
Moschonis et al. (2010)												x														x		2
Moschonis et al. (2011)																										x		1
Mostafai et al. (2018);												x	x	x		x												4
Natri et al. (2006)			x					x		x											x	x	x		x			7
Neyestani et al. (2012)														x														1
Neyestani et al. (2014)		x			x							x	x							x						x		6
Neyestani et al. (2015)												x														x		2
Nikooyeh et al. (2011)			x								x		x	x		x			x		x		x					8
Nikooyeh et al. (2016)											x	x	x	x				x	x		x				x			8
Nikooyeh et al. (2019)																					x							1
Nissen et al. (2014)																									x			1
Öhlund et al. (2017)		x		x			x													x								4
Palacios et al. (2005)								x																		x		2
Panunzio et al. (2003)								x																				1
Powers et al. (2016)		x																										1
Recker et al. (1985)										x																		1
Rich-Edwards et al. (2011)		x		x	x					x	x	x								x								7
Rosenblum et al. (2012)											x		x	x														3
Salehi, et al. (2018)											x	x		x					x									4
Sandmann et al. (2015)	x																											1
Shab-Bidar et al. (2011a)			x								x		x	x		x			x		x							7
Shab-Bidar et al. (2011b)														x														1
Shab-Bidar et al. (2015)													x			x												2
Sun et al. (2011)		x																										1
Suzuki et al. (2014)																										x		1

Tangpricha et al. (2003)			x					x			x										x	x	x					6
Tenta et al. (2011)										x		x									x					x		4
Toxqui et al. (2013a)																					x							1
Toxqui et al. (2013b)											x													x				2
Toxqui et al. (2014)							x																					1
Trautvetter et al. (2014)							x																					1
Tripkovic et al. (2017)							x				x	x									x							4
Urbain et al. (2011)																							x					1
Wagner et al. (2008)							x				x										x		x					4
Wang et al. (2017)		x																										1
Woo et al. (2007)			x																									1
Zhang et al. (2016)																										x		1
Zhu et al. (2005)										x		x														x		3
Zhu et al. (2008)										x																		1
