# Supplementary figures and images for: Fortification of Staple Foods for Household Use with Vitamin D: An Overview of Systematic Reviews
Source: Nutrients. 2023 Aug 26;15(17):3742. doi: 10.3390/nu15173742 (PMC10489979; doi:10.3390/nu15173742)

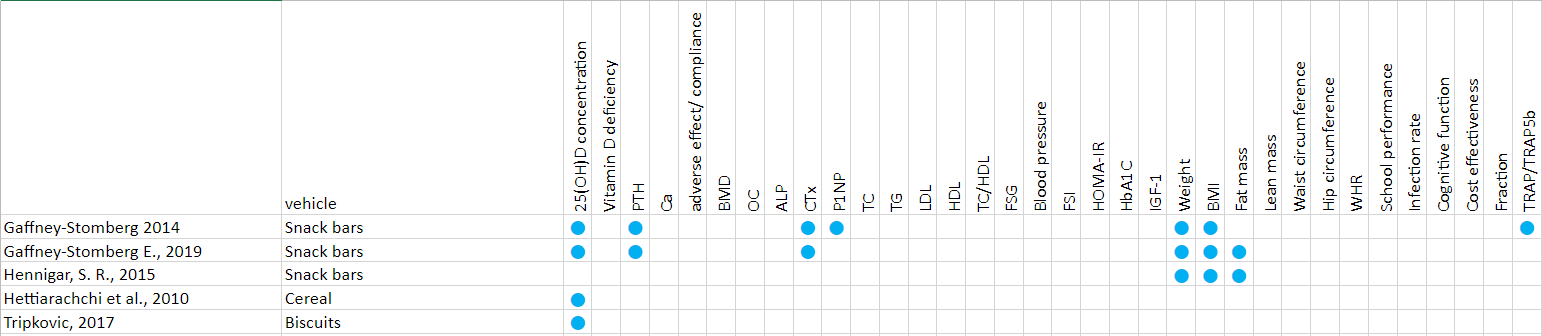

Supplement: Supplementary file 1 [file nutrients-15-03742-s001.zip › Supplementary_File_S10_Outcomes_investigated_in_studies_on_vitamin_D_fortification_of_cereal_biscuits_and_snack_bars.png]

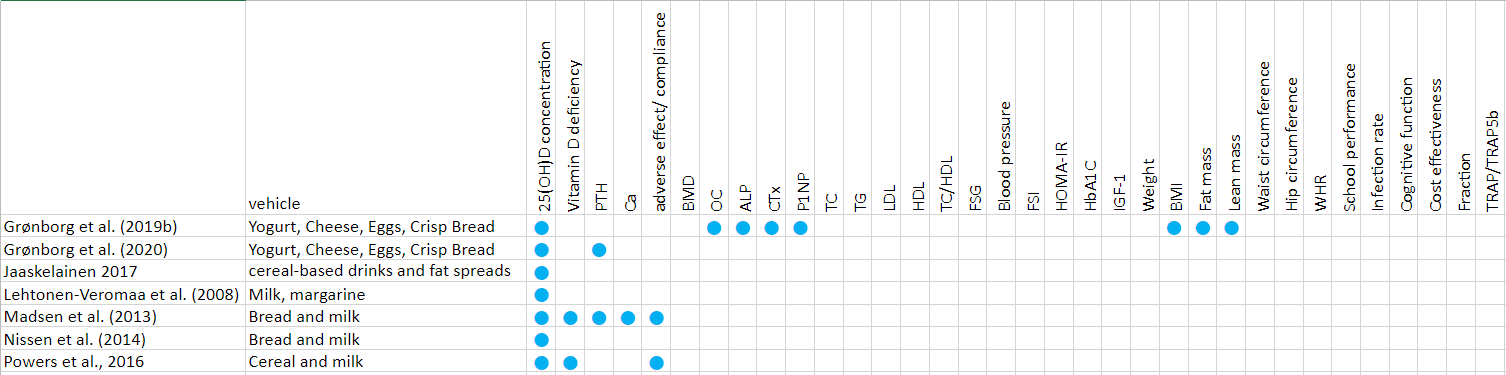

Supplement: Supplementary file 1 [file nutrients-15-03742-s001.zip › Supplementary_File_S11_Outcomes_investigated_in_studies_on_vitamin_D_fortification_of_multiple_food_items.png]

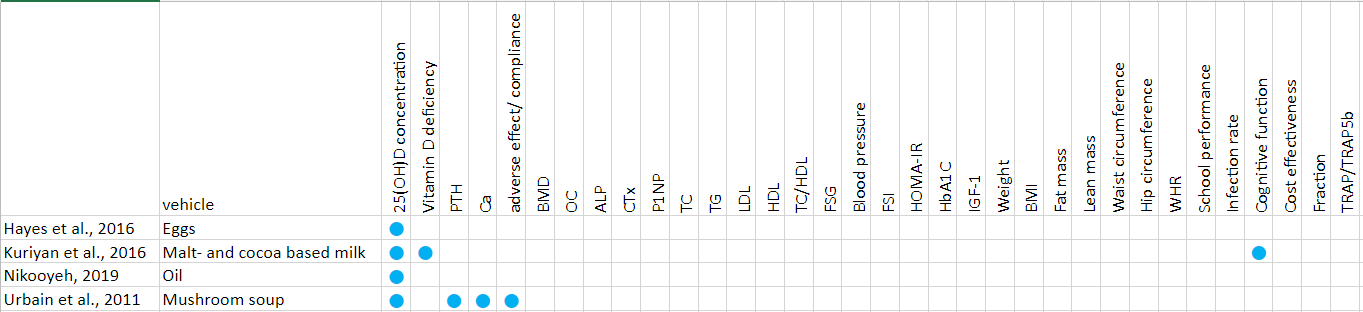

Supplement: Supplementary file 1 [file nutrients-15-03742-s001.zip › Supplementary_File_S12_Outcomes_investigated_in_studies_on_vitamin_D_fortification_of_oils_and_biofortification_of_eggs_and_mushrooms.png]

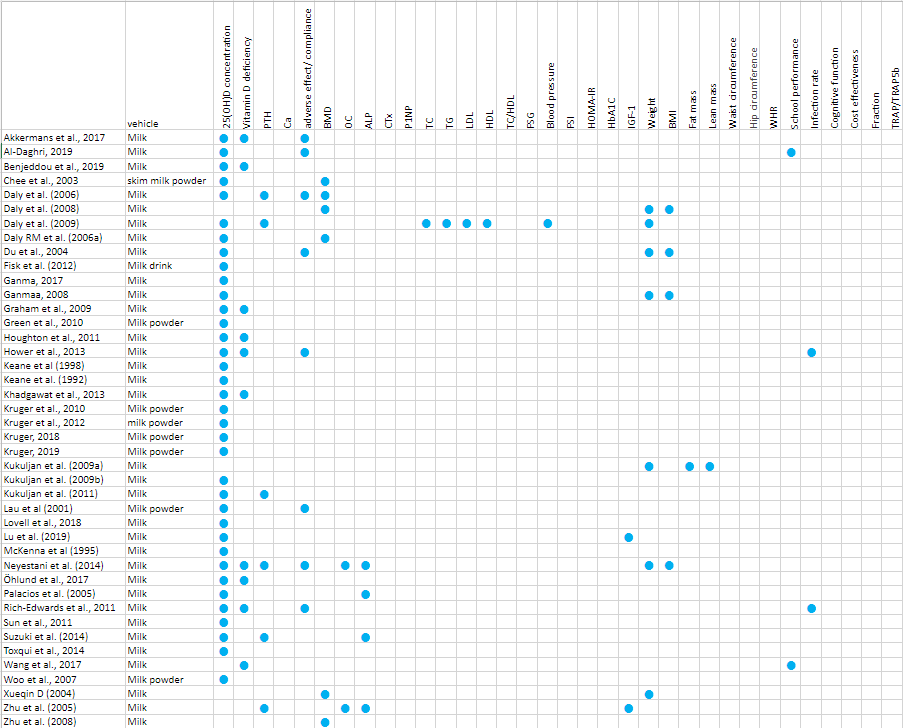

Supplement: Supplementary file 1 [file nutrients-15-03742-s001.zip › Supplementary_File_S6_Outcomes_investigated_in_studies_on_vitamin_D_fortification_of_milk_and_milk_powder.png]

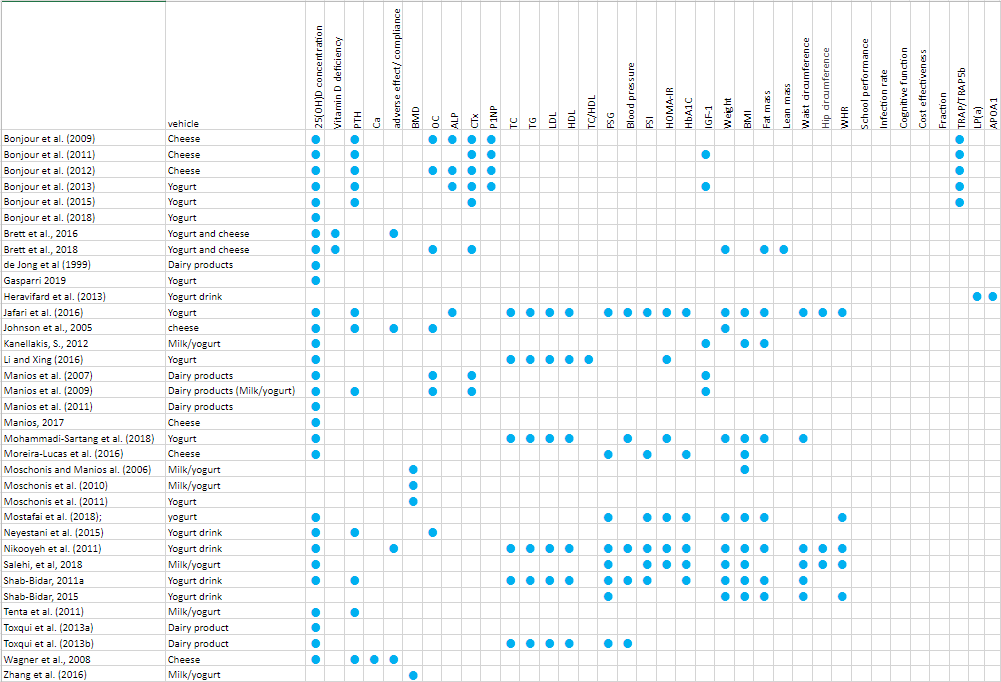

Supplement: Supplementary file 1 [file nutrients-15-03742-s001.zip › Supplementary_File_S7_Outcomes_investigated_in_studies_on_vitamin_D_fortification_of_dairy_product.png]

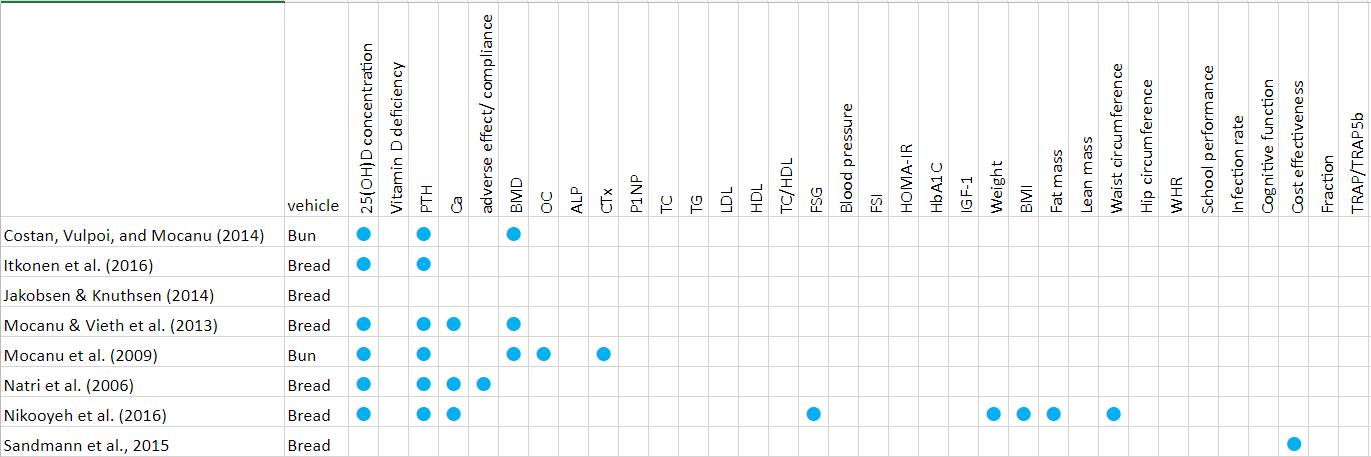

Supplement: Supplementary file 1 [file nutrients-15-03742-s001.zip › Supplementary_File_S8_Outcomes_investigated_in_studies_on_vitamin_D_fortification_of_bun_and_bread.png]

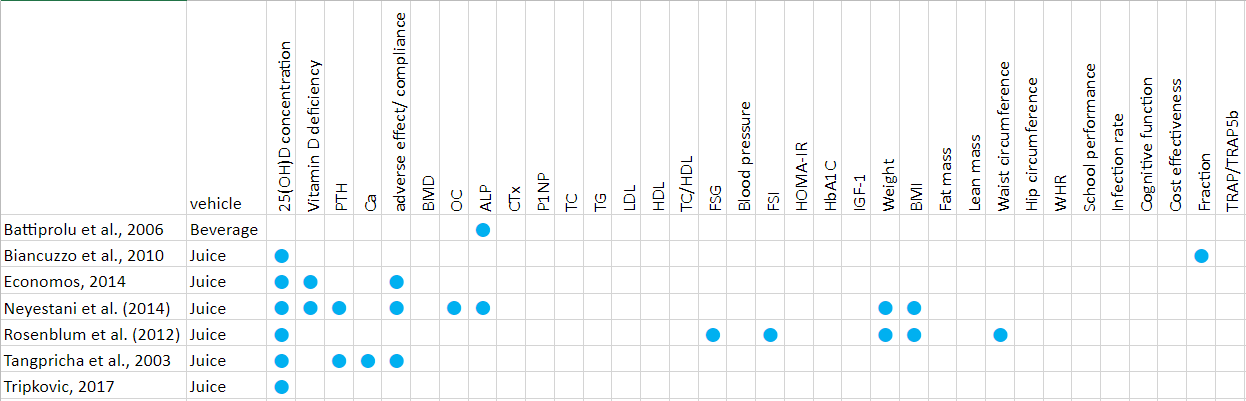

Supplement: Supplementary file 1 [file nutrients-15-03742-s001.zip › Supplementary_File_S9_Outcomes_investigated_in_studies_on_vitamin_D_fortification_of_juice_and_beverages.png]
